# Targeting androgen receptor and the variants by an orally bioavailable Proteolysis Targeting Chimeras compound in castration resistant prostate cancer

**DOI:** 10.1016/j.ebiom.2023.104500

**Published:** 2023-03-07

**Authors:** Chiu-Lien Hung, Hao-Hsuan Liu, Chih-Wei Fu, Hsun-Hao Yeh, Tsan-Lin Hu, Zong-Keng Kuo, Yu-Chin Lin, Mei-Ru Jhang, Chrong-Shiong Hwang, Hung-Chih Hsu, Hsing-Jien Kung, Ling-Yu Wang

**Affiliations:** a Department of Preclinical Drug Discovery Technology, Biomedical Technology and Devices Research Labs, Industrial Technology Research Institute, Hsinchu 31040, Taiwan; b Department of Biochemistry and Molecular Biology, Chang Gung University, Taoyuan 33302, Taiwan; c Division of Hematology-Oncology, Chang Gung Memorial Hospital at Linkou, Taoyuan 33305, Taiwan; d College of Medicine, Chang Gung University, Taoyuan 33305, Taiwan; e Institute of Molecular and Genomic Medicine, National Health Research Institutes, Zhunan, Miaoli County 35053, Taiwan; f Research Center of Cancer Translational Medicine, Taipei Medical University, Taipei 11031, Taiwan; g Ph.D. Program for Cancer Biology and Drug Discovery, College of Medical Science and Technology, Taipei Medical University, Taipei 11031, Taiwan

**Keywords:** PROTAC, AR, AR-V7, Castration resistant prostate cancer, Enzalutamide resistance

## Abstract

**Background:**

Despite the advent of improved therapeutic options for advanced prostate cancer, the durability of clinical benefits is limited due to inevitable development of resistance. By constitutively sustaining androgen receptor (AR) signaling, expression of ligand-binding domain truncated AR variants (AR-V(ΔLBD)) accounts for the major mechanism underlying the resistance to anti-androgen drugs. Strategies to target AR and its LBD truncated variants are needed to prevent the emergence or overcome drug resistance.

**Methods:**

We utilize Proteolysis Targeting Chimeras (PROTAC) technology to achieve induced degradation of both full-length AR (AR-FL) and AR-V(ΔLBD) proteins. In the ITRI-PROTAC design, an AR N-terminal domain (NTD) binding moiety is appended to von-Hippel-Lindau (VHL) or Cereblon (CRBN) E3 ligase binding ligand with linker.

**Findings:**

*In vitro* studies demonstrate that ITRI-PROTAC compounds mechanistically degrade AR-FL and AR-V(ΔLBD) proteins via ubiquitin-proteasome system, leading to impaired AR transactivation on target gene expression, and inhibited cell proliferation accompanied by apoptosis activation. The compounds also significantly inhibit enzalutamide-resistant growth of castration resistant prostate cancer (CRPC) cells. In castration-, enzalutamide-resistant CWR22Rv1 xenograft model without hormone ablation, ITRI-90 displays a pharmacokinetic profile with decent oral bioavailability and strong antitumor efficacy.

**Interpretation:**

AR NTD that governs the transcriptional activities of all active variants has been considered attractive therapeutic target to block AR signaling in prostate cancer cells. We demonstrated that utilizing PROTAC for induced AR protein degradation via NTD represents an efficient alternative therapeutic strategy for CRPC to overcome anti-androgen resistance.

**Funding:**

The funding detail can be found in the Acknowledgements section.

**Research in context**

**Evidence before this study**

PROTAC is an emerging therapeutic modality for cancer treatment. Several PROTAC compounds have been reported to target androgen receptor (AR) with demonstrated efficacies toward AR protein degradation and anti-proliferation of prostate tumors in animal studies. Significantly, the ongoing phase 2 clinical study of Arvinas ARV-110 has shown encouraging results of antitumor activity and benefit for patients with metastatic castration-resistant prostate cancer. Most reported AR PROTAC degrade only full-length AR protein. MTX-23 that is derived from a binder of AR DNA-binding domain (DBD), is reported to possess degradation ability towards both full-length and a truncated AR variant, AR-V7. Many preclinical and clinical evidence showed that truncated AR variants, which lack the hormone binding domain for regulation and remain constitutively active, accounts for the major mechanism underlying castration and second-generation anti-androgen drug resistance. Targeting the truncated AR variants has thus been considered an attractive approach to overcome therapy resistance. ESSA Pharma has recently reported AR degraders targeting the N-terminal domain (NTD), capable of inducing truncated AR degradation.

**Added value of this study**

We present PROTAC compounds targeting the AR NTD, which commonly exists in full length and all active truncated AR variants. These compounds effectively degrade both forms of AR including at least two species of the clinical relevant, truncated variants in a castration-resistant cell model. In animal studies, we further demonstrate ITRI-90 with a decent oral availability and a strong antitumor efficacy in a therapy-resistant prostate cancer model. Without hormone ablation, single use of ITRI-PROTAC is sufficient to greatly impair tumor growth. Given that compensating hormone synthesis in tumor niche occurs after standard hormone therapy and restrains the efficacies of androgen antagonist drugs, the ability of eliminating all active AR proteins regardless of high hormone environment is an efficient strategy to block AR signaling.

**Implications of all the available evidence**

AR NTD that governs the activities of all AR variants, has been considered challenging for conventional drug development. This preclinical study shows that PROTAC technology represents an ideal alternative approach to inhibit AR signaling via NTD targeting and subsequent degradation of all active AR forms. The ITRI-PROTAC is anticipated to provide advantages for the intervention of therapy-resistant prostate cancers.

## Introduction

As of 2020, prostate cancer is the second most common malignancy and the 5th leading cause of cancer death in men worldwide.^1^ Although many therapeutic options for advanced prostate cancer are currently available, the durability of clinical benefit is limited by the inevitable acquisition of resistance, making the disease management challenging.^2^ As a hormone regulated malignancy, prostate cancer depends on AR signaling for disease development. Although androgen deprivation therapy (ADT) is effective in alleviating tumor burden during early stages, virtually all disease presentations rapidly develop to a castration-resistant state (CRPC).^3^ While second generation anti-androgen drugs such as enzalutamide and abiraterone have proven clinical benefits for CRPC treatment, primary or acquisition of resistance to these drugs is commonly documented in patients with subsequent lethal disease progression. It is known that the therapy-resistant cancer cells remain highly dependent on sustained AR signaling resulting from mechanisms such as AR amplification, mutations, splice variants expression, or activation by alternative co-activators.^4^ Given that cross drug resistance can rise from commonly altered cellular mechanisms, resistance to apalutamide and darolutamide, two recently approved potent AR antagonists that are mechanistically similar to enzalutamide,^5^, ^6^, ^7^, ^8^ is anticipated to remain as the major issue in advanced prostate cancer management. Developing alternative therapeutic approaches is therefore an urgent need.

Substantial pre-clinical and clinical evidence shows that expression of constitutively active AR variants (AR-V) that lack the ligand-binding domain (LBD) associates with anti-androgen resistance.^2^^,^^4^^,^^9^, ^10^, ^11^ Among them, AR-V7 is the most predominately detected variant in patient specimens. Following anti-androgen treatment, the frequency of detectable AR-V7 in patient samples is dramatically increased.^12^^,^^13^ The AR-V7 positivity and expression level is associated with resistance to enzalutamide and abiraterone, as well as higher risk of biochemical relapse and poor overall and progression-free survival.^2^^,^^12^, ^13^, ^14^, ^15^, ^16^, ^17^, ^18^ Importantly, the increase of AR-V7 upon ADT was observed *in vitro* and shown to regulate a distinct transcriptional program contributing to CRPC and stem cell potential.^19^, ^20^, ^21^, ^22^ Silencing AR-V7 effectively inhibits CRPC cell growth,^23^, ^24^, ^25^ highlighting this variant as a crucial driver of the therapeutic resistance. In addition to AR-V7, several other variants lacking LBD are detected in clinical CRPC samples.^26^^,^^27^ Those such as AR-V3 and AR-V9 are further demonstrated to co-express with AR-V7 in metastatic CRPC tumors predictive to be abiraterone resistance.^28^^,^^29^ Given the significance of these LBD-truncated AR-Vs in CRPC progression, targeting the truncated variants has become an emerging strategy for CRPC treatment.^15^

Proteolysis targeting chimeras (PROTAC) is a rapidly developing technology that has become a promising therapeutic modality for cancer treatment, and a number of PROTACs are being evaluated in clinical trials.^30^ Also known as bivalent chemical protein degraders, PROTACs comprise two moieties connecting a linker, a protein of interest (POI)-binding moiety and an E3 ubiquitin ligase-binding moiety. By forming a POI-PROTAC-E3 ligase ternary complex, the POI is targeted for degradation via ubiquitin-proteasome system. To date, several PROTAC degraders have been reported with the design of targeting full-length AR. Arvinas's ARV-110,^31^^,^^32^ ARCC-4,^33^ TD-802,^34^ and a series of ARD PROTACs,^35^, ^36^, ^37^, ^38^ are designed by appending modified AR antagonists that target AR LBD to E3 ligase ligands. These compounds either by employing cereblon (CRBN)- (ARV-110, TD-802, ARD-2128) or von Hippel-Lindau (VHL)- (ARCC4, ARD-266, ARD-69, ARD-61) mediated degradation, effectively inhibit tumor proliferation in both androgen-dependent as well as CRPC models. Lee et al. recently reported MTX-23 which uses a DNA-binding domain (DBD) binder as the warhead in the PROTAC design. MTX-23 is capable of degrading both AR-FL and AR-V7, and shows potent inhibition of CRPC tumor growth with combined use of enzalutamide.^39^ Given the promising results achieved in these studies, we were inspired to take an alternative strategy targeting AR via its N-terminal domain (NTD). Herein, we present an orally available PROTAC compound that degrades both full-length and LBD-truncated AR with a potent antitumor activity as a monotherapy in a CRPC xenograft model.

## Methods

### Chemistry

#### General experiment and information

Starting materials, reagents and solvents were purchased from commercial suppliers (Sigma–Aldrich, Acros, TCI, Alfa, Combi-Blocks, Matrix and Fischer) and were used as received without further purification. 1H spectra were obtained on Varian AS500 500 NMR-spectrometer in the indicated solvents. Chemical shifts are expressed in ppm (δ units) relative to TMS signal as internal standard. Flash column chromatography was performed on column packed with Merck silica gel 60 (0.063–0.200 μm). Preparative HPLC was carried out on a Jasco with a UV-975 detector (Inertsil ODS-3 column 30 × 250 mm, 5 μm reverse phase column, eluting CH_3_CN/H_2_O with 0.1% TFA or without TFA, flow rate 42 mL/min, UV254 nm). Mass spectra with electronic impact (MS) were recorded from Micromass Quattro triple quadrupole mass spectrometer (Waters, USA). Solvents were reagent grade and, when necessary, they were purified and dried by standard methods. Concentration of the reaction solutions involved the use of rotary evaporator at reduced pressure.

#### Scheme of compound synthesis

**Figure:**
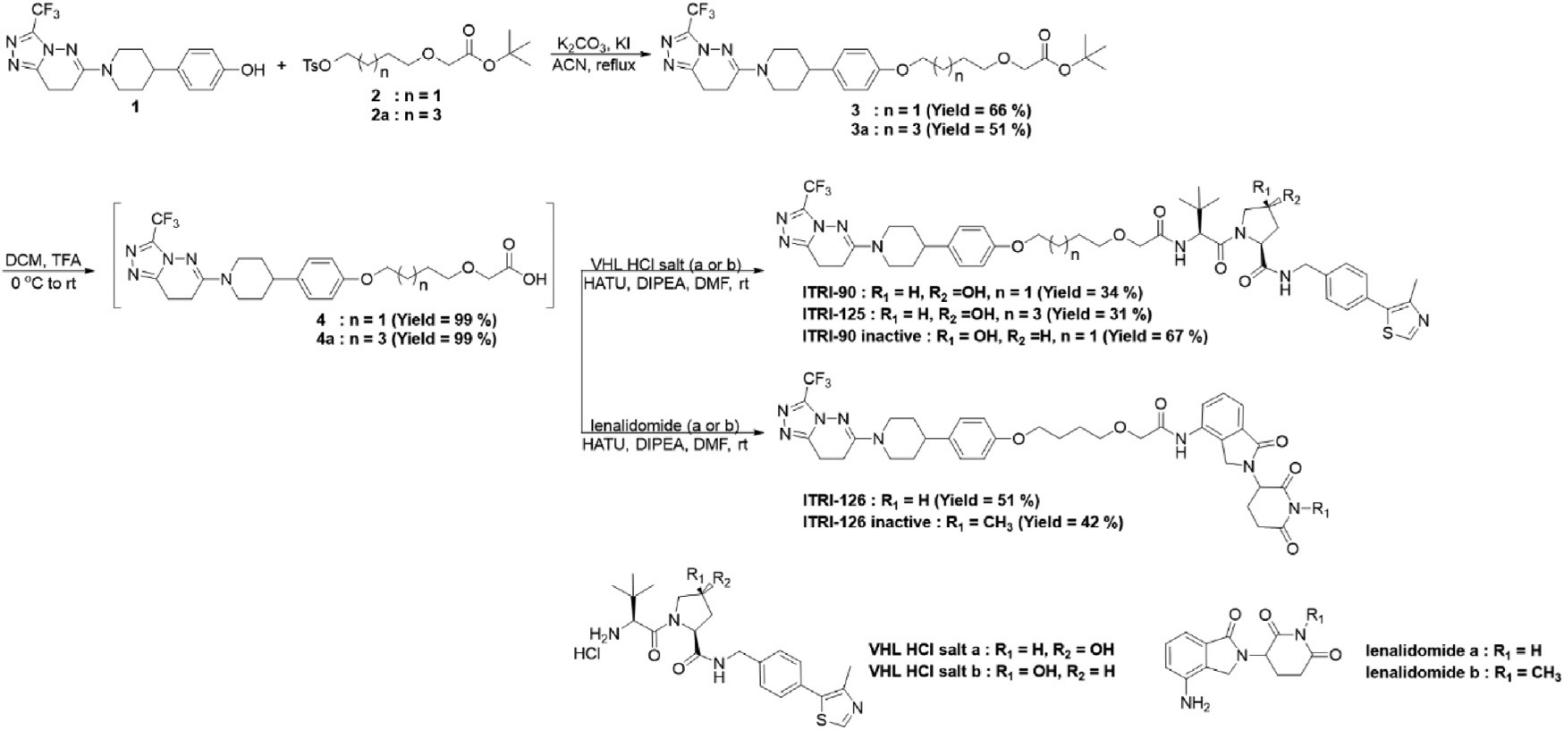

The structures of all final compounds were verified by ^1^H-NMR, ^13^C-NMR, and high-resolution mass spectrometry (Figs. S1–S7). Purity of all final compounds was determined by analytical HPLC and listed in the compound description below. All final compounds reached >95% purity (Figs. S8 and S9).

#### General procedure for synthesis of compounds 3 and 3a

A mixture of 1 (1.0 eq), 2 or 2a (2.0 eq), K_2_CO_3_ (3.0 eq) and KI (0.5 eq) in ACN (0.1 M) was stirred at reflux for overnight. The mixture was filtered with DCM, concentrated and purified by flash chromatography to get 3 or 3a. The structures of compounds 3 and 3a were verified by ^1^H-NMR (Fig. S1).

#### General procedure for synthesis of compounds 4 and 4a

A solution of 3 or 3a (1.0 eq) in DCM/TFA (1/1) was stirred at room temperature for 2 h. Then the reaction solution was concentrated to dryness to give 4 or 4a without further purification.

#### General procedure for synthesis of ITRI-90 and ITRI-125

A mixture of 4 or 4a (1.0 eq) and VHL HCl salt (1.1 eq) in DMF (0.1 M) was added DIPEA (3.0 eq) and HATU (1.1 eq), the mixture was stirred at room temperature for 5 h. The mixture was diluted with H_2_O and extracted with EtOAc. The combined organic phase was washed with brine, dried over Na_2_SO_4_, concentrated and purified by reverse phase preparative HPLC to get ITRI-90 or ITRI-125.

#### General procedure for synthesis of ITRI-126

A mixture of 4 (1.0 eq) and lenalidomide (1.1 eq) in DMF (0.1 M) was added DIPEA (3.0 eq) and HATU (1.1 eq), the mixture was stirred at room temperature for 5 h. The mixture was diluted with H_2_O and extracted with EtOAc. The combined organic phase was washed with brine, dried over Na_2_SO_4_, concentrated and purified by reverse phase preparative HPLC to get ITRI-126.

#### tert-butyl 2-(4-(4-(1-(3-(trifluoromethyl)-7,8-dihydro-[1,2,4]triazolo[4,3-b]pyridazin-6-yl)piperidin-4-yl)phenoxy)butoxy)acetate (compound 3)

Yield = 66%. ^1^H-NMR (500 MHz, CDCl3): δ 7.10 (d, J = 8.0 Hz, 2H), 6.85 (d, J = 8.5 Hz, 2H), 4.31 (s, 2H), 3.99–3.96 (m, 4H), 3.58 (t, J = 6.5 Hz, 2H), 3.21 (t, J = 8.0 Hz, 2H), 3.00 (t, J = 12.5 Hz, 2H), 2.80–2.72 (m, 3H), 1.95–1.85 (m, 4H), 1.82–1.76 (m, 2H), 1.72–1.62 (m, 2H), 1.47 (s, 9H).

#### tert-butyl 2-((6-(4-(1-(3-(trifluoromethyl)-7,8-dihydro-[1,2,4]triazolo[4,3-b]pyridazin-6-yl)piperidin-4-yl)phenoxy)hexyl)oxy)acetate (compound 3a)

Yield = 51%. ^1^H-NMR (500 MHz, CDCl3): δ 7.11 (d, J = 9.0 Hz, 2H), 6.85 (d, J = 9.0 Hz, 2H), 4.32 (s, 2H), 3.95–3.93 (m, 4H), 3.52 (t, J = 6.5 Hz, 2H), 3.23 (t, J = 7.5 Hz, 2H), 3.01 (t, J = 12.5 Hz, 2H), 2.82–2.73 (m, 3H), 1.97–1.80 (m, 4H), 1.79–1.76 (m, 2H), 1.73–1.61 (m, 4H), 1.48 (s, 9H), 1.47–1.42 (m, 2H).

#### (2S,4S)-1-((S)-3,3-dimethyl-2-(2-(4-(4-(1-(3-(trifluoromethyl)-7,8-dihydro-[1,2,4]triazolo[4,3-b]pyridazin-6-yl)piperidin-4-yl)phenoxy)butoxy)acetamido)butanoyl)-4-hydroxy-N-(4-(4-methylthiazol-5-yl)benzyl)pyrrolidine-2-carboxamide (ITRI-90)

Yield = 34%. ^1^H-NMR (500 MHz, CDCl3): δ 8.66 (s, 1H), 7.36–7.32 (m, 5H), 7.18 (d, J = 9.0 Hz, 1H), 7.10 (d, J = 8.0 Hz, 2H), 6.83 (d, J = 8.0 Hz, 2H), 4.72 (t, J = 7.5, 8.5 Hz, 1H), 4.57–4.53 (m, 2H), 4.49 (d, J = 9.0 Hz, 1H), 4.34 (dd, J = 5.5, 15.3 Hz, 2H), 4.09 (d, J = 11.0 Hz, 1H), 3.97–3.89 (m, 4H), 3.62–3.56 (m, 3H), 3.21 (t, J = 8.0, 8.0 Hz, 2H), 3.02–2.95 (m, 2H), 2.79 (t, J = 7.0, 8.5 Hz, 2H), 2.50 (s, 3H), 2.14–2.09 (m, 1H), 2.00–1.92 (m, 3H), 1.86–1.78 (m, 7H), 1.71–1.63 (m, 3H), 1.25 (s, 1H), 0.94 (s, 9H). ^13^C-NMR (125 MHz, MeOH-d_4_), δ(ppm): 174.39, 172.04, 171.74, 161.59, 158.99, 152.80, 148.99, 147.84, 140.23, 138.78, 133.41, 131.48, 130.51, 130.35, 128.94, 128.67, 115.59, 72.61, 71.06, 70.75, 68.69, 60.88, 58.15, 58.09, 43.82, 42.87, 38.92, 37.22, 34.28, 27.39, 27.20, 26.92, 21.10, 17.94, 15.85. ESI-MS m/z:calculated for C45H57F3N9O6S 908.4103, found [M + H]^+^ = 908.4099.; Purity = 95.4%. LCMS [M + H]^+^ = 908.3.

#### (2S,4S)-1-((S)-3,3-dimethyl-2-(2-(6-(4-(1-(3-(trifluoromethyl)-7,8-dihydro-[1,2,4]triazolo[4,3-b]pyridazin-6-yl)piperidin-4-yl)phenoxy)hexyloxy)acetamido)butanoyl)-4-hydroxy-N-(4-(4-methylthiazol-5-yl)benzyl)pyrrolidine-2-carboxamide (ITRI-125)

Yield = 31%. ^1^H-NMR (500 MHz, MeOH-d_4_): δ 8.83 (s, 1H), 7.44 (s, 2H), 7.40 (d, J = 6.5 Hz, 2H), 7.11 (d, J = 7.5 Hz, 2H), 6.80 (d, J = 7.5 Hz, 2H), 4.68 (s, 1H), 4.56 (d, J = 13.5 Hz, 2H), 4.51 (s, 1H), 4.00–3.92 (m, 4H), 3.87 (d, J = 10.5 Hz, 1H), 3.80 (d, J = 11.0 Hz, 1H), 3.56 (s, 2H), 3.19 (s, 2H), 3.03 (t, J = 11.5 Hz, 2H), 2.95 (s, 2H), 2.77 (t, J = 11.0 Hz, 1H), 2.45 (s, 3H), 2.25–2.20 (m, 1H), 2.11–2.07 (m, 1H), 1.87 (d, J = 12.5 Hz, 2H), 1.76 (s, 2H), 1.68 (d, J = 8.0 Hz, 4H), 1.51 (s, 4H), 0.96 (s, 9H). ^13^C-NMR (125 MHz, MeOH-d_4_), δ(ppm): 174.31, 172.10, 171.71, 161.60, 159.06, 152.82, 149.00, 147.84, 140.21, 138.72, 133.41, 131.48, 130.52, 130.36, 128.95, 128.66, 115.59, 72.86, 71.05, 70.73, 68.84, 60.82, 58.14, 58.05, 57.98, 43.71, 42.87, 38.91, 37.22, 34.27, 30.62, 30.38, 27.06, 27.00, 26.92, 21.10, 17.94, 15.86. ESI-MS m/z: calculated for C47H61F3N9O6S 936.4385, found [M + H]^+^ = 936.4412.; Purity = 97.2%.

#### N-(2-(2,6-dioxopiperidin-3-yl)-1-oxoisoindolin-4-yl)-2-(4-(4-(1-(3-(trifluoromethyl)-7,8-dihydro-[1,2,4]triazolo[4,3-b]pyridazin-6-yl)piperidin-4-yl)phenoxy)butoxy)acetamide (ITRI-126)

Yield = 51%. ^1^H-NMR (500 MHz, CDCl3): δ 8.30 (s, 1H), 8.07 (s, 1H), 7.74 (dd, J = 2.5, 7.5 Hz, 2H), 7.49 (t, J = 8.0, 8.0 Hz, 1H), 7.10 (d, J = 8.0 Hz, 2H), 6.84 (d, J = 8.5 Hz, 2H), 5.18 (dd, J = 5.0, 13.25 Hz, 1H), 4.46 (d, J = 8.0 Hz, 2H), 4.11 (s, 2H), 4.01 (t, J = 6.0, 5.5 Hz, 2H), 3.59 (t, J = 6.0, 5.5 Hz, 2H), 3.21 (t, J = 8.0, 7.5 Hz, 2H), 2.99 (t, J = 13.0, 12.0 Hz, 2H), 2.84–2.70 (m, 4H), 2.32 (ddd, J = 5.5, 12.5, 26.0 Hz, 1H), 2.19–2.16 (m, 1H), 1.93–1.87 (m, 5H), 1.70–1.62 (m, 7H). LCMS [M + H]^+^ = 737.0. ^13^C-NMR (125 MHz, CDCl3), δ(ppm): 171.03, 169.51, 168.76, 167.76, 158.76, 157.43, 145.12, 137.19, 133.74, 132.77, 131.70, 129.25, 127.65, 125.68, 121.37, 114.51, 71.58, 70.05, 67.44, 51.81, 46.18, 46.03, 41.50, 32.91, 31.44, 26.22, 26.01, 23.27, 20.60, 17.54. ESI-MS m/z: calculated for C36H40F3N8O6 737.3014, found [M + H]^+^ = 737.3017.; Purity = 95.9%. LCMS [M + H]^+^ = 737.0.

#### (2S,4S)-1-((S)-3,3-dimethyl-2-(2-(4-(4-(1-(3-(trifluoromethyl)-7,8-dihydro-[1,2,4]triazolo[4,3-b]pyridazin-6-yl)piperidin-4-yl)phenoxy)butoxy)acetamido)butanoyl)-4-hydroxy-N-(4-(4-methylthiazol-5-yl)benzyl)pyrrolidine-2-carboxamide (ITRI-90 inactive)

Yield = 67%. ^1^H-NMR (500 MHz, CDCl3): δ 8.83 (s, 1H), 7.52 (t, J = 6.0 Hz, 1H), 7.34 (s, 4H), 7.10–7.07 (m, 3H), 6.80 (d, J = 8.5 Hz, 2H), 4.69 (d, J = 9.0 Hz, 1H), 4.62 (dd, J = 6.5, 14.5 Hz, 2H), 4.51–4.45 (m, 2H), 4.29 (dd, J = 5.0, 15.0 Hz, 2H), 3.97–3.88 (m, 5H), 3.80 (d, J = 11.0 Hz, 1H), 3.55 (t, J = 6.0 Hz, 2H), 3.22 (t, J = 8.0 Hz, 2H), 2.98 (t, J = 12.5 Hz, 2H), 2.78 (t, J = 8.0 Hz, 2H), 2.75–2.70 (m, 1H), 2.50 (s, 3H), 2.32 (d, J = 14.5 Hz, 1H), 2.19–2.14 (m, 1H), 1.91 (d, J = 13.5 Hz, 2H), 1.84–1.76 (m, 4H), 1.69–1.61 (m, 2H), 0.91 (s, 9H). ^13^C-NMR (125 MHz, CDCl3), δ(ppm): 172.62, 171.78, 169.88, 158.91, 157.62, 151.30, 147.10, 137.91, 136.93, 132.46, 130.27, 129.60, 128.31, 127.59, 119.23, 117.08, 114.53, 71.45, 71.06, 69.96, 67.44, 59.97, 58.60, 56.35, 46.12, 43.48, 41.58, 35.14, 35.12, 33.00, 26.29, 25.91, 20.46, 17.40, 15.27. ESI-MS m/z: calculated for C45H57F3N9O6S 908.4116, found [M + H]^+^ = 908.4099; Purity = 99.2%.

#### N-(2-(1-methyl-2,6-dioxopiperidin-3-yl)-1-oxoisoindolin-4-yl)-2-(4-(4-(1-(3-(trifluoromethyl)-7,8-dihydro-[1,2,4]triazolo[4,3-b]pyridazin-6-yl)piperidin-4-yl)phenoxy)butoxy)acetamide (ITRI-126 inactive)

Yield = 42%. ^1^H-NMR (500 MHz, CDCl3): δ 8.31 (s, 1H), 7.70 (dd, J = 2.0, 7.5 Hz, 2H), 7.45 (t, J = 7.5 Hz, 1H), 7.07 (d, J = 8.5 Hz, 2H), 6.80 (d, J = 8.0 Hz, 2H), 5.12 (dd, J = 5.5, 13.5 Hz, 1H), 4.38 (s, 2H), 4.08 (s, 2H), 3.98 (t, J = 5.5 Hz, 2H), 3.67 (t, J = 6.0 Hz, 2H), 3.18 (t, J = 8.0 Hz, 2H), 3.12 (s, 3H), 2.99–2.86 (m, 3H), 2.81–2.69 (m, 4H), 2.28–2.22 (m, 4H), 2.13–2.10 (m, 1H), 1.97–1.83 (m, 5H), 1.66–1.61 (m, 2H). ^13^C-NMR (125 MHz, CDCl3), δ(ppm): 171.12, 170.01, 168.81, 167.70, 158.78, 157.40, 137.15, 133.75, 132.90, 131.64, 129.20, 127.61, 125.60, 121.36, 119.30, 117.15, 114.47, 71.55, 70.03, 67.42, 52.44, 46.33, 46.00, 41.47, 32.87, 31.91, 27.08, 26.19, 25.97, 22.60, 20.52, 17.45. ESI-MS m/z: calculated for C37H42F3N8O6 751.3171, found [M + H]^+^ = 751.3174; Purity = 100%.

### Affinity selection mass spectrometer binding assay

Affinity selection mass spectrometer (ASMS)^40^ binding assay was employed for the screen and relative binding affinity assessment of AR NTD binding compounds. Briefly, GST tagged AR-NTD protein (221–320) (Abcam ab157902) at final concentration of 100 nM was incubated with 100 nM of individual candidate compounds in PBS for 1 h at 37 °C. The protein-compound mixture was filtered through 10K MWCO pore sized Nanosep centrifugal column (PALL Life science) with thorough wash steps to separate protein-bound and unbound compounds. The protein-bound compounds were subsequently extracted and analyzed by ABSCIEX 4000 QTRAP LC-ESI MS/MS (AB Sciex, Framingham, MA) coupled to an Agilent 1200 HPLC system. A Waters S XBridge RP18, 3.5 μm, 4.6 × 20 mm column was used as the stationary phase (Waters Corporation, Milford, MA, USA). Gradient elution was carried out using water with 0.1% formic acid (solvent A) and acetonitrile with 0.1% formic acid (solvent B) as a mobile phase. Positive ion polarity scanning was used in electrospray (ESI) mass-triggered fraction collection to monitor the target mass. Quantification of protein-bound compounds was determined against calibration curves established with drug standards. For relative binding affinity assessment, same procedure and condition was carried out for each compound without target protein incubated in the reaction as background controls. Relative binding affinity was calculated as percentage of input compound quantity after background value subtraction.

### *In vitro* stability in plasma

The *in vitro* stability of the PROTAC compounds was performed in BALB/C (BALB/cAnNCrlBltw) mice plasma. The reactions were initiated by adding 10 μM PROTAC compounds to 0.5 mL of pre-warmed plasma solution. The assays were performed in Thermomixer at 37 °C. During the incubation, 20 μL samples were taken at 0, 30, 60, 120, and 240 min time points, and added to 120 μL of acetonitrile to deproteinize the plasma. After 10 min of centrifugation at 14,000 rpm, the supernatants were collected for subsequent HPLC-UV analysis (Waters 1525 Binary and 2707 Autosampler).

### Metabolic stability in liver microsomes

The mouse CD-1 pooled liver microsome (Corning Product ID: 452701, Lot: 0010003) was used to test compound stability. The reaction was carried out in 100 mM, pH 7.4 phosphate buffer containing microsome (0.8 mg/mL), NADP^+^ (1.3 mM), glucose-6-phosphate (3.3 mM), glucose-6-phosphate dehydrogenase (0.4 U/mL), magnesium chloride (3.3 mM) and the test article (1 μM) with 1% (v/v) final concentration of DMSO. The reaction was incubated at 37 °C with shaking. At 0, 10, 20, and 30 min time points, 20 μL of the reaction mixture was taken and quenched immediately with 120 μL acetonitrile. The quenched sample were centrifuged, and the supernatant was then analyzed by LC-MS/MS (ABI4000Q-TRAP). 1 μM warfarin and 1 μM verapamil with same reaction conditions was used as negative and positive control, respectively.

### Cell culture

Cell lines LNCaP (LNCaP-FGC, RRID:CVCL_1379), CWR22Rv1 (22Rv1, RRID:CVCL_1045), VCaP (RRID:CVCL_2235), DU145 (RRID:CVCL_0105), PC3 (RRID:CVCL_0035) and HEK293T (293T, RRID:CVCL_0063) were purchased from ATCC, PNT2 was purchased from Sigma–Aldrich (Cat# 95012613, RRID:CVCL_2164). All cell lines were cultured under conditions as recommended. C4-2B and C4-2B/Enz^R^ cells were generous gifts from Dr. Allen C. Gao at UC Davis. Parental C4-2B cells were cultured in the RPMI-1640 medium supplemented with 10% FBS, while C4-2B/Enz^R^ cells were maintained in the RPMI-1640 complete medium containing 10 μM enzalutamide. All cell lines were recently validated with STR analysis, and confirmed to be mycoplasma-free by Mycoplasma PCR Detection Kit (GeneCopoeia, Rockville, MD USA). Enzalutamide (Enz) was purchased from Selleck Chemicals (Houston, TX, USA).

### Plasmids

The Flag-AR(ΔLBD) plasmid (pLenti4/TO/Flag-TC-AR) was cloned from cDNA obtained from CWR22Rv1 as described in the previous study.^41^ The Flag-AR plasmid (pLenti4/TO/Flag-AR) was cloned similarly by inserting full-length AR cDNA sequence into the modified form of lentiviral expression vector pLenti4/TO/V5-DEST (Thermo Fisher Scientific, Waltham, MA, USA) in frame with sequence encoding the Flag epitope.^42^

### Immunoblotting and ubiquitination of AR

To obtain total lysates, cells were lysed in RIPA buffer (50 mM Tris–HCl pH 8.0, 150 mM NaCl, 1% NP-40, 0.5% Na-deoxycholate, 0.5% SDS plus protease inhibitors). The lysates were resolved by SDS-polyacrylamide gel electrophoresis, and the protein expression was analyzed by western blotting using primary antibodies against AR (Millipore Cat# 06-680, RRID:AB_310214), AR-V7 (RevMAb Biosciences Cat# 31-1109-00, RRID:AB_2716436), GAPDH (Cell Signaling Technology Cat# 2118, RRID:AB_561053) and Actin (Sigma–Aldrich Cat# A5441, RRID:AB_476744). pLenti4/TO/Flag-AR or pLenti4/TO/Flag-TC-AR plasmid was co-transfected with HA-ubiquitin plasmid into 293T cells. 24 h after transfection, the cells were treated with 10 μM of the PROTAC compounds for 16 h, followed by 4-h treatment of 5 μM MG132. The cells were lysed with lysis buffer (50 mM Tris–HCl pH7.5, 150 mM NaCl, 0. 5% Triton X-100, 10% glycerol, 1 mM EDTA plus protease inhibitors) containing 1 ng/mL of N-ethylmaleimide, followed by immunoprecipitation with anti-Flag M2 (Sigma–Aldrich Cat# F3165, RRID:AB_259529) antibody. The ubiquitinated AR was then detected by western blotting using anti-HA (Sigma–Aldrich Cat# H3663, RRID:AB_262051) antibody.

### Luciferase assay

LNCaP and CWR22Rv1 cells were seeded in 24-well plates and cultured in RPMI1640 medium containing 10% charcoal dextran-treated-FBS for hormone deprivation 1 day prior to transfection. Cells were co-transfected with *KLK3* promoter luciferase plasmid as described previously,^43^ and pRL-SV40 Renilla luciferase plasmid. On the following day, the cells were pre-treated with 1 nM dihydrotestosterone (DHT) for 2 h and subsequently treated with ITRI-PROTAC compounds for another 16 h. 48 h post-transfection, the cells were lysed and luciferase activity was detected using Dual-Luciferase Assay Kit (Promega, Madison, WI, USA). All samples were tested in triplicate, and the luciferase relative light units (RLUs) were normalized against the Renilla values acquired from each sample.

### Quantitative RT-PCR

TRIzol™ Reagent (Thermo Fisher Scientific) was used to isolate total RNA, and iScript™ cDNA Synthesis Kit (Bio-Rad, Hercules, CA, USA) was used for cDNA synthesis. The cDNA expression level of each gene was then quantified by the Bio-Rad CFX Real-Time PCR detection system using iTaq Universal SYBR Green Supermix (Bio-Rad) and primers listed in Table S1. All samples were tested in triplicate, and the expression levels were normalized against *RPL13A* and *GAPDH* cDNA levels using Bio-Rad CFX software.

### Cell viability, proliferation and caspase activity

Cells were seeded in triplicate in 96-well plates 1 day prior to drug treatment for all assays. For viability test and IC_50_ assessment, the alamarBlue™ Cell Viability Reagent (Thermo Fisher Scientific) was added to cells 7 days after PROTAC treatment, followed by fluorescence detection as the manufacturer instructed. The IC_50_ was calculated by dose-inhibition regression. For proliferation monitoring, 2, 4, 6 and 8 days after PROTAC treatment (day 0), viable cells were detected by MTT Cell Proliferation Kit I (Sigma–Aldrich) according to the manufacturer's instructions. Cellular caspase activity was measured by Caspase-Glo 3/7 Assay System (Promega) 1 or 2 days after the drug treatment.

### *In vivo* experiments in mice

#### Ethics statement

All procedures were performed according to the “Guide for the Care and Use of Laboratory Animals” issued by the National Institutes of Health (NIH publication no. 85–23, revised 1996). The experimental protocols were prepared and approved by the IACUC of Industrial Technology Research Institute before study (IACUC approval number: ITRI-IACUC-2020-001 and ITRI-IACUC-2020-014).

#### Pharmacokinetic analysis

Six to eight-week-old male BALB/c (BALB/cAnNCrlBltw) mice weighing between 22 and 30 g were purchased from BioLASCO Taiwan Co., Ilan, Taiwan. *In vivo* PK analysis of the PROTAC compounds was performed with 2 mg/kg for intravenous injection (IV, n = 2), 10 mg/kg for intraperitoneal injection (IP, n = 3), and 10 or 30 mg/kg for oral (PO, n = 3) dosing route. Blood samples from the mice were collected using Microvette® CB 300 EDTA blood collection tubes by a lancet at 0.167 h (IV only), 0.5, 1, 2, 4, 7 and 24 h time points. The plasma was then subjected to LC-MS analysis to quantitate the drug concentration against calibration curves that were established with drug standards prepared in mice plasma.

### *In vivo* efficacy studies

Immunodifficient male C.B-17 SCID mice (NOD.CB17-*Prkdc*^*scid*^/NcrCrlBltw) at 4–6 weeks of age were purchased from BioLASCO Taiwan Co., Ilan, Taiwan. All mice were housed in conventional cages in the AAALAC Full Accreditation (2011) in Industrial Technology Research Institute and were allowed to acclimatize and recover from shipping-related stress for one week prior to the study. The health of the mice was monitored by daily observations. Animals were kept in rooms at temperature of 22–26 °C with 40–70% humidity, positive pressure, 60% air recirculation, ventilation rate 15–20 changes per hour, and a controlled light–dark cycle (12–12 h). The order and location of each animal cage was randomly assigned to minimize potential confounders. CWR22Rv1 cells (5 × 10^6^) were suspended in 100 μL of PBS with 30% Matrigel and subcutaneously implanted into the right flank of male C.B-17 SCID mice. Tumors were measured with calipers, and tumor size was calculated as follows: tumor volume (V) = (L × S^2^)/2 (L, longest diameter, mm; S, shortest diameter, mm). When mean tumor volume reached 150–200 mm^3^, the mice were randomly assigned into indicated groups (n = 5–6/group) and the day of initiating treatments was assigned as day 0. In the study using intraperitoneal (IP) injection, the mice received vehicle control (NMP: Cremophor: PBS = 1:2:7 (v:v:v)), ITRI-90 (10 mg/kg, BID) or ITRI-126 (10 mg/kg, BID) for the indicated days. In the study using oral (PO) gavage, the mice received vehicle control or ITRI-90 (100 mg/kg, BID) for 21 days. Tumor size and body weight of the animals were monitored and recorded two to three times per week. Mice were sacrificed when one of the tumor reached over 1000 mm^3^ in size. Tumors were collected and immediately subjected to liquid nitrogen freezing and homogenization. The homogenized tumor samples were stored at −80 °C for subsequent western blotting analysis. The drug antitumor efficacy was presented as percentages of TGI of each tested animal, calculated as follows: [1 − (final tumor volume − initial tumor volume of the treated group)/average of (final tumor volume − initial tumor volume of the vehicle group)] × 100. Body weight of each mouse was also compared to that on the first day of treatment (day 0) and expressed as a percentage of day 0 value. All data points were included for calculation and the values are presented as mean ± SEM. Statistical analysis of the differences between two drug and vehicle treated groups was determined by two-tailed Student's t test. For negative control, same procedures as described above were followed. PC3 cells (5 × 10^6^) mixing with 50% matrigel were subcutaneously implanted into both flanks of 8 male C.B-17 SCID mice, followed by random assignment for oral administration of vehicle control or ITRI-90 drug (100 mg/kg, BID) treatment (n = 4/group).

### Statistical analysis

All *in vitro* experiments were performed at least three times in triplicate. Statistical analysis of the differences between two groups was determined by two-tailed Student's t test. *p* values less than 0.05 were considered to be statistical significant.

### Role of the funding source

The funders had no role in the study design, data collection and analysis, decision to publish, or preparation of the manuscript.

## Results

### Strategy of PROTAC compounds development

**Fig. 1:**
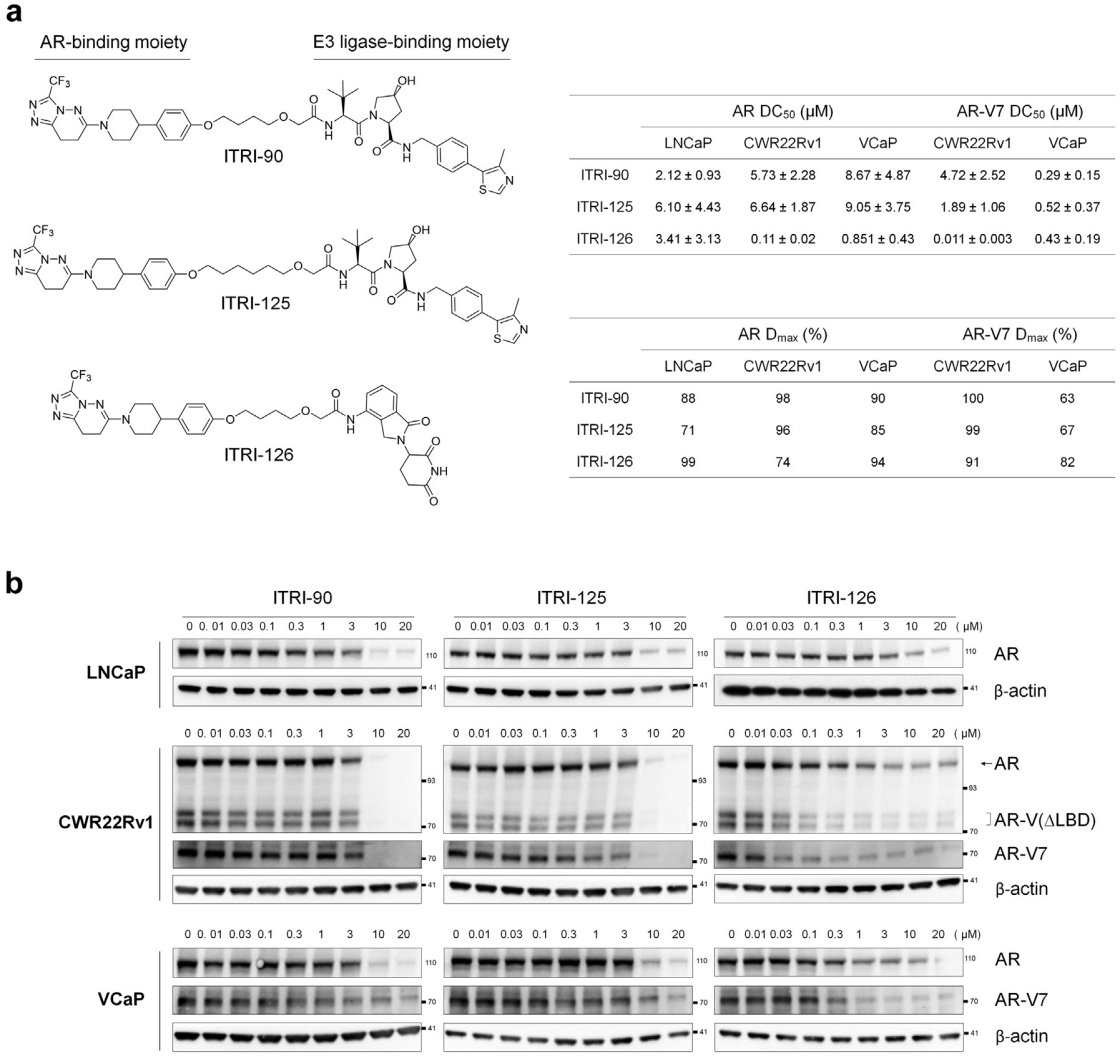
PROTAC-based AR degraders ITRI-90, ITRI-125 and ITRI-126 reduce AR and AR-V7 proteins in prostate cancer cell lines. (a) Chemical structure of the PROTAC AR degraders. (b) Representative western blots of AR protein levels in LNCaP, CWR22Rv1 and VCaP cells treated with the PROTAC degraders for 24 h. AR was detected by N-terminal antibody which also detects C-terminal truncated AR-V(ΔLBD) in CWR22Rv1 cells. AR-V7 specific antibody was used for AR-V7 detection. 4%–12% gradient gel was used to separate different species of AR-V(ΔLBD) near 75 kDa in CWR22Rv1 cell lysates. DC_50_ indicating the concentration required to achieve 50% degradation, was calculated based on normalized full-length AR and AR-V7 band intensity. D_max_ is the maximum degradation achieved at 20 μM compound treatment.

Potential AR NTD-binding ligands were screened by affinity selection mass spectrometer (ASMS) using AR NTD (221–320) purified protein. A series of EPI compounds, Niclosamide, and AZD3514 were selected as candidates for structure modification and the subsequent warhead screen. The EPI compounds are AR NTD binders proven to inhibit AR signaling by interfering AR-coactivator interaction.^44^, ^45^, ^46^ Niclosamide is identified to induce AR-V7 degradation through a proteasome dependent pathway.^47^ AZD3514 on the other hand, is an orally available AR inhibitor known to inhibit androgen-dependent and -independent signaling by directly binding with AR.^48^^,^^49^ These encouraging results prompted us to develop the PROTAC platform based on the core structures of these compounds, in the hope to improve the degradation potency and the specificity toward AR NTD domain. A collection of compounds derived from the core structures of these candidates, categorized as ARN binders, NCS binders and AZD binders were subjected to ASMS screen. Compounds with the strongest relative binding affinity in each category were further selected for PROTAC synthesis. The structures and relative binding affinity of these AR NTD binders are summarized in Table S1. Various lengths of linker moiety and VHL binding ligand as the E3 ligase binding moiety^50^ were used for the subsequent PROTAC synthesis. The relative binding affinity of these PROTACs with AR NTD were also determined by ASMS assay. Of note, the binding affinity dropped after assembling the warheads to PROTAC compounds in all categories. Meanwhile, the potencies and stabilities of the PROTACs were assessed by *in vitro* assays including AR degradation and cell viability test in CWR22Rv1 cells, as well as plasma stability and liver microsomal stability assays. Interestingly, although ARN-5- and NCS-4-derived PROTACs showed strong relative binding affinity with AR NTD, they failed to reach desired potency of viability inhibition or acceptable *in vitro* stability. AZD-1-derived ITRI-90 and ITRI-125 on the other hand, displayed profiles fulfilling the *in vitro* potency and stability criteria, and were thus used for further characterization (Table S1, Fig. 1a).

### ITRI-PROTAC compounds effectively degrade AR

Given the promising *in vitro* profiles displayed in ITRI-90 and ITRI-125, ITRI-126, which utilizes the same AR binding moiety but CRBN ligand pomalidomide as E3 ligase-binding moiety was synthesized^51^ (Fig. 1a). The relative binding affinity of ITRI-126 with AR NTD assessed by the ASMS binding assay is comparable to the VHL-based compounds (20% recovery of input, Table S1). Three prostate cancer cell lines expressing either AR-FL with a T878A mutation (LNCaP), or expressing both AR-FL and multiple LBD-truncated AR-Vs including AR-V7 and AR-V9 that represent validated CRPC *in vitro* models (CWR22Rv1 and VCaP cells),^17^^,^^52^ were used to determine the potency of ITRI-90, ITRI-125 and ITRI-126. Antibody recognizing AR N-terminus was used to detect both AR-FL and AR-V(ΔLBD). Remarkably, after 24 h treatment, ITRI-PROTAC effectively reduced AR-FL and AR-V(ΔLBD) proteins in these cell lines in a dose-dependent manner (Fig. 1b). By contrast, an inactive VHL isomer-derived ITRI-90 and a N-methylated CRBN-derived ITRI-126 that serve as negative controls, did not inhibit the AR protein expression (Fig. S10a and b), suggesting an E3-ligase recruitment dependent mechanism. In CWR22Rv1 cells, the two AR-V(ΔLBD) species at approximately 75–80 kDa of molecular weight, presumably AR-V7 and AR-V9, showed a consistent decreasing pattern, suggesting that ITRI-PROTAC strategically represents an effective modality capable of targeting most clinically relevant AR-Vs that harbor exon 1–3.^53^ Down-regulation of AR-V7 in CWR22Rv1 and VCaP cells was further confirmed by a specific AR-V7 antibody (Fig. 1b). Moreover, in AR/AR-V-negative-DU145 cells that transiently express ectopic ARΔLBD, ITRI-PROTAC treatment also strongly inhibited the protein expression, confirming the specificity of induced AR degradation independent of LBD domain (Fig. S10c). The DC_50_ of these PROTACs for AR degradation is similar across three cell lines within low micromolar range, with the D_max_ reaching over 60% and up to 100%. Of note, all three compounds showed higher potency for AR-V7 degradation over AR-FL in CWR22Rv1 and VCaP cells, possibly reflecting a difference of protein stability regulation between full-length and alternatively spliced AR. Conversely, ITRI-126 achieved the lowest DC_50_ for both AR-FL and AR-V7, suggesting that in agreement with the consensus of PROTAC development, CRBN ligand-based compound with lower molecular weight in nature, may significantly increase the potency compared to VHL ligand-based compounds.

**Fig. 2:**
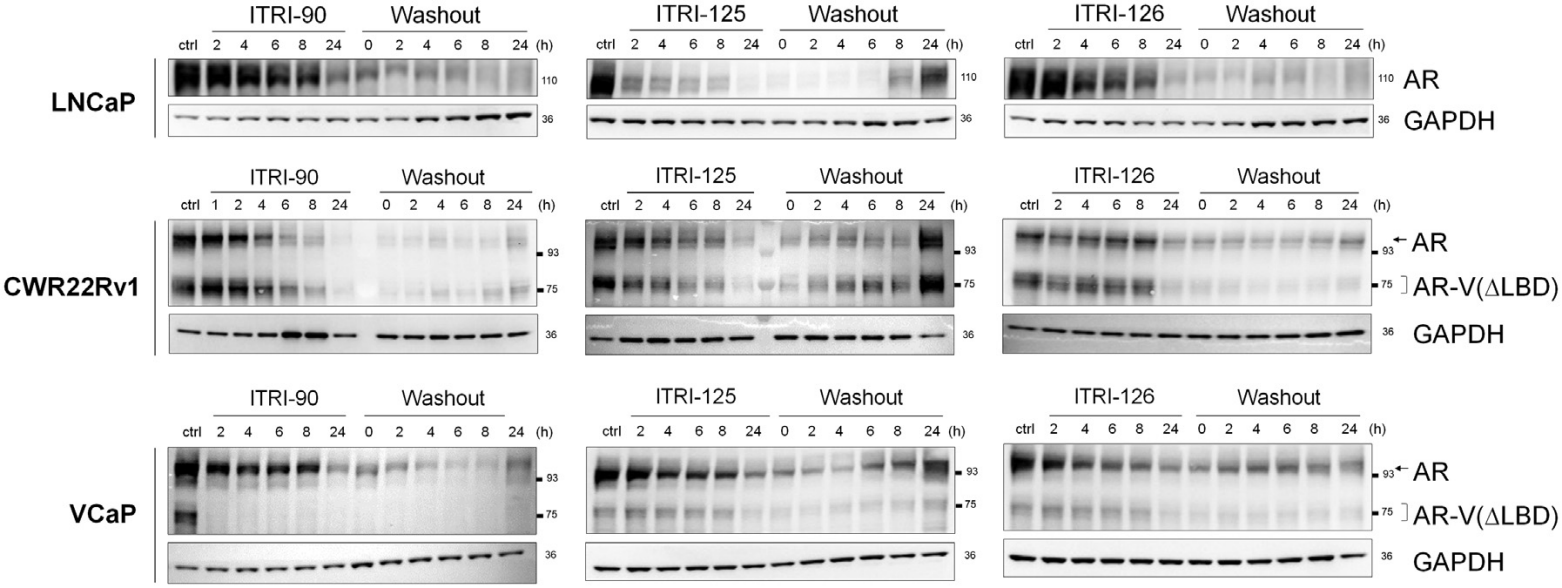
Kinetics of AR degradation upon PROTAC treatment and washout in LNCaP, CWR22Rv1 and VCaP cells. Representative western blots of AR and AR-V(ΔLBD) proteins at the indicated time points upon 10 μM of compound treatment and washout. In washout experiment, 24 h after compound treatment, the cells were rinsed with PBS three times and cultured in fresh medium for the indicated time period before harvesting.

To understand the temporal kinetics and duration of AR degradation, cells treated with the ITRI compounds were analyzed at different timepoint post-drug removal. As shown in Fig. 2, these compounds displayed different kinetics of degradation and sustainability after drug washout. While all compounds achieved AR and AR-V(ΔLBD) degradation by 24 h treatment, ITRI-90 and ITRI-126 treatment appeared to sustain low protein levels up to 24 h after drug washout. By contrast, ITRI-125 lost the degrading effects on both full-length and truncated AR within 8 h post drug removal in all cell lines with the protein expression bouncing back to near full levels 24 h after washout.

### Proteasome-dependent degradation of AR by ITRI-PROTAC compounds

**Fig. 3:**
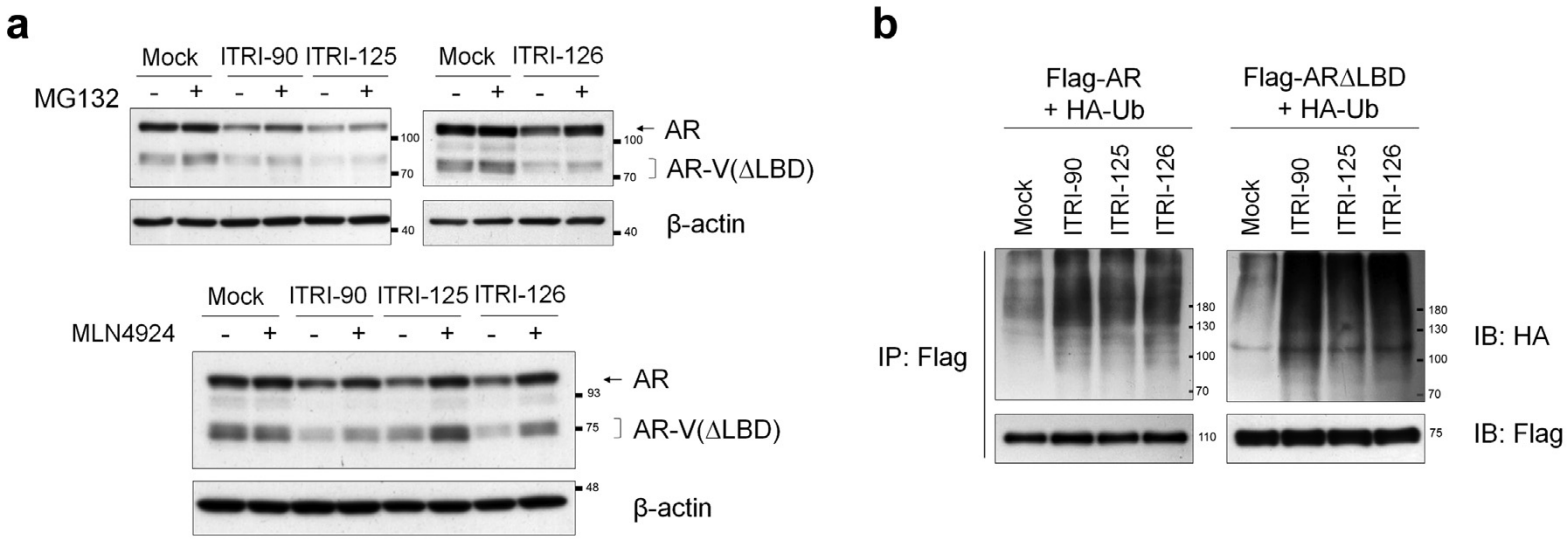
The ITRI compounds degrade AR protein levels via ubiquitin-proteasome dependent system. (a) Western blotting of the AR and AR-V(ΔLBD) protein levels upon 10 μM PROTAC compounds with or without MG132 and MLN4924 treatment in CWR22Rv1 cells. Cells were incubated with ITRI-90 or ITRI-125 for 6 h followed by 2 h of 5 μM MG132 treatment. For ITRI-126, cells were incubated with the compound for 10 h, followed by 2 h of MG132 treatment. Cells were treated with all three compounds for 6 h, followed by 6 h of 500 nM MLN4924 treatment. (b) Ubiquitination of AR and ARΔLBD proteins upon PROTAC treatment. 293T cells transfected with Flag-AR or Flag-ARΔLBD and HA-Ub were incubated with 10 μM PROTAC compounds and MG132. AR proteins were immunoprecipitated by anti-Flag antibody, and ubiquitination of the AR proteins were detected by western blotting with anti-HA antibody.

Following the evidence showing the dependence of E3-ligase recruitment for AR down-regulation (Fig. S10a and b), we next sought to verify the ubiquitin-proteasome-dependent target degradation for the actions of ITRI compounds. Proteasome inhibitor MG132 and NEDD-8 inhibitor MLN4924, which inactivates Cullin-ring E3 ubiquitin ligases were used. Given that both MG132 and MLN4924 are reported to inhibit AR either by suppressing its transactivation or gene expression,^54^^,^^55^ to limit potential interference on the PROTAC-mediated AR degradation, cells were incubated with MG132 and MLN4924 for limited time after ITRI compounds treatment. Fig. 3a clearly showed that the ITRI compounds-mediated degradation of AR and AR-V(ΔLBD) was recovered by MG132 and MLN4924 treatment. To further detect AR ubiquitination upon PROTAC treatment, 293T cells were transiently transfected with AR or ARΔLBD along with HA-ubiquitin, followed by immunoprecipitation of the AR and subsequent detection of ubiquitinated AR proteins using western blotting. The results indeed showed that both the full-length and LBD-truncated AR proteins were strongly ubiquitinated in ITRI compounds treated cells (Fig. 3b). These results together indicate a degradation mechanism consistent with the PROTAC molecular design.

### ITRI-PROTAC decreases AR signaling and inhibits cell viability

**Fig. 4:**
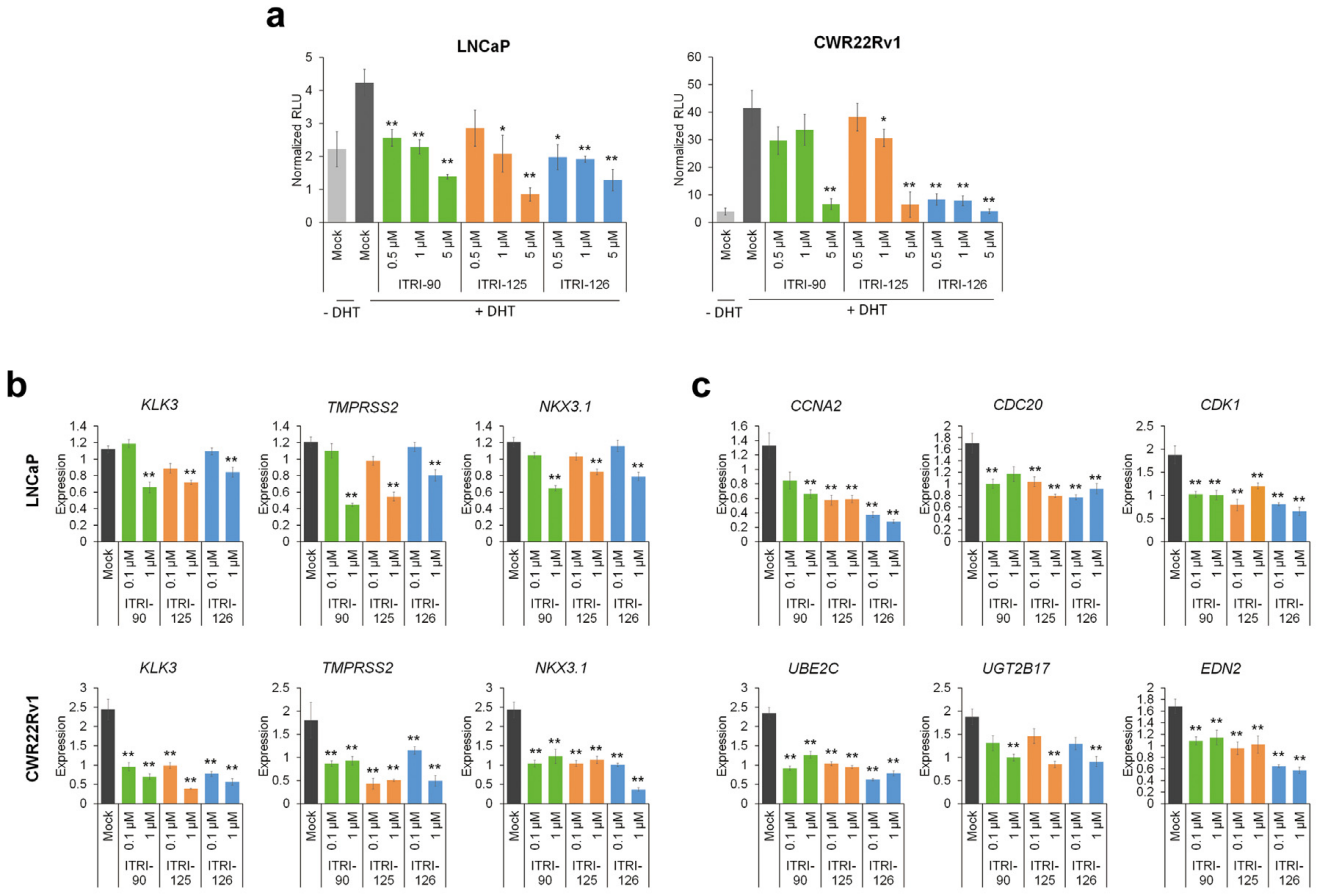
The PROTAC compounds inhibit AR transcriptional activity and target gene expression. (a) *KLK3* promoter-luciferase reporter assay was carried out in LNCaP and CWR22Rv1 cells upon DHT stimulation in the presence of the PROTAC compounds. AR (b) and AR-V7 (c) target gene expression in LNCaP and CWR22Rv1 cells treated with the PROTAC compounds for 24 h were analyzed by qRT-PCR. *GAPDH* and *RPL13A* was used as reference genes for gene expression normalization. All data with error bars is presented as mean ± standard deviation. Asterisks indicate statistical significance between the PROTAC treated groups and mock (∗*p* < 0.05; ∗∗*p* < 0.01, two-tailed Student's t test).

The consequence of AR degradation was further assessed by its transactivation activity on the enhancer/promoter of prostate-specific antigen (PSA) gene *KLK3*. In both LNCaP and CWR22Rv1 cells, DHT treatment after hormone depletion induced the *KLK3* promoter activity, whereas the degraders significantly impaired such activation in a dose-dependent manner (Fig. 4a). The expression of AR target genes such as *KLK3*, *NKX3.1* and *TMPRSS2* was consistently decreased 24 h after ITRI compounds treatment (Fig. 4b). Genes such as *CCNA2*, *CDC20*, *CDK1*, *UBE2C*, *UGT2B17* and *EDN2*, known to be specifically up-regulated by AR-V7, were also significantly reduced in CWR22Rv1 cells upon the drug treatment (Fig. 4c). These results confirmed the effect of induced AR degradation on both AR and AR-V7 signaling inhibition. We noticed that the AR target expression was less efficiently inhibited by the ITRI degraders compared to enzalutamide at low concentration, but reached comparable extent at higher concentration, whereas the AR-V7 targets were specifically inhibited by the degraders but not enzalutamide (Fig. S11). These results confirmed the distinct mechanisms of AR inhibition mediated via NTD-targeted protein degradation or direct blockade of ligand binding.

**Fig. 5:**
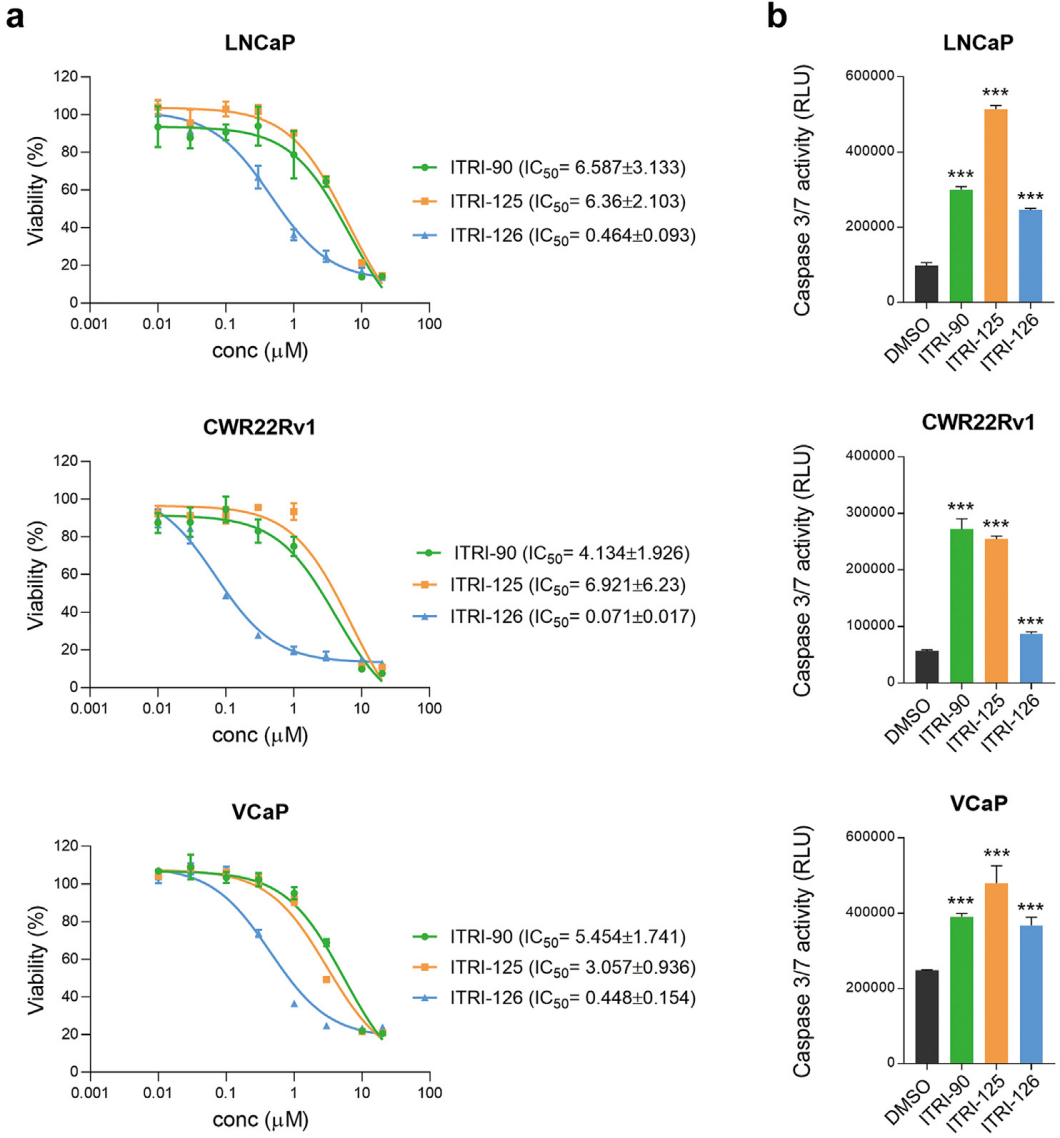
The AR degraders promote apoptosis and inhibit cancer cell growth. (a) Cell viability was assessed in cells cultured in normal hormone condition with PROTAC compounds treated for 7 days. Viability was calculated against the value of mock treatment (0 μM). (b) Caspase 3/7 activity in LNCaP, CWR22Rv1 and VCaP cells treated with 10 μM PROTAC compounds for 24 h was detected by a luminescence-based assay, indicated by relative light unit (RLU). Error bars is presented as mean ± standard deviation. Asterisks indicate statistical significance between the PROTAC treated groups and mock (∗∗∗*p* < 0.001, two-tailed Student's t test).

Alamar blue cell viability assay revealed that ITRI-PROTACs specifically and effectively inhibited cancer cell proliferation. After 7 days of treatment, the compounds strongly inhibited cell growth by over 90%, with the IC_50_ of ITRI-90 and ITRI-125 ranging from 3 to 6 μM, while that of ITRI-126 ranges from 0.07 to 0.4 μM in all three cell lines (Fig. 5a). At 10 μM, all AR degraders rapidly activated caspase 3/7 within 24 h of treatment (Fig. 5b), indicating induced apoptosis in the cells. Given the lower IC_50_ achieved by ITRI-126, we noted that lower concentrations of ITRI-126 induced strong caspase activity two days after drug treatment (Fig. S12), implying that low dose of ITRI-126 with longer exposure significantly induced cytotoxicity *in vitro*, while high dose may overwhelm the cells with non-specific effects. Importantly, in immortalized normal prostate epithelial PNT2 cells which expresses very low level of AR, the PROTAC compounds showed no effect on the cell viability (Fig. S13), highlighting a specific antitumor effect of these compounds.

**Fig. 6:**
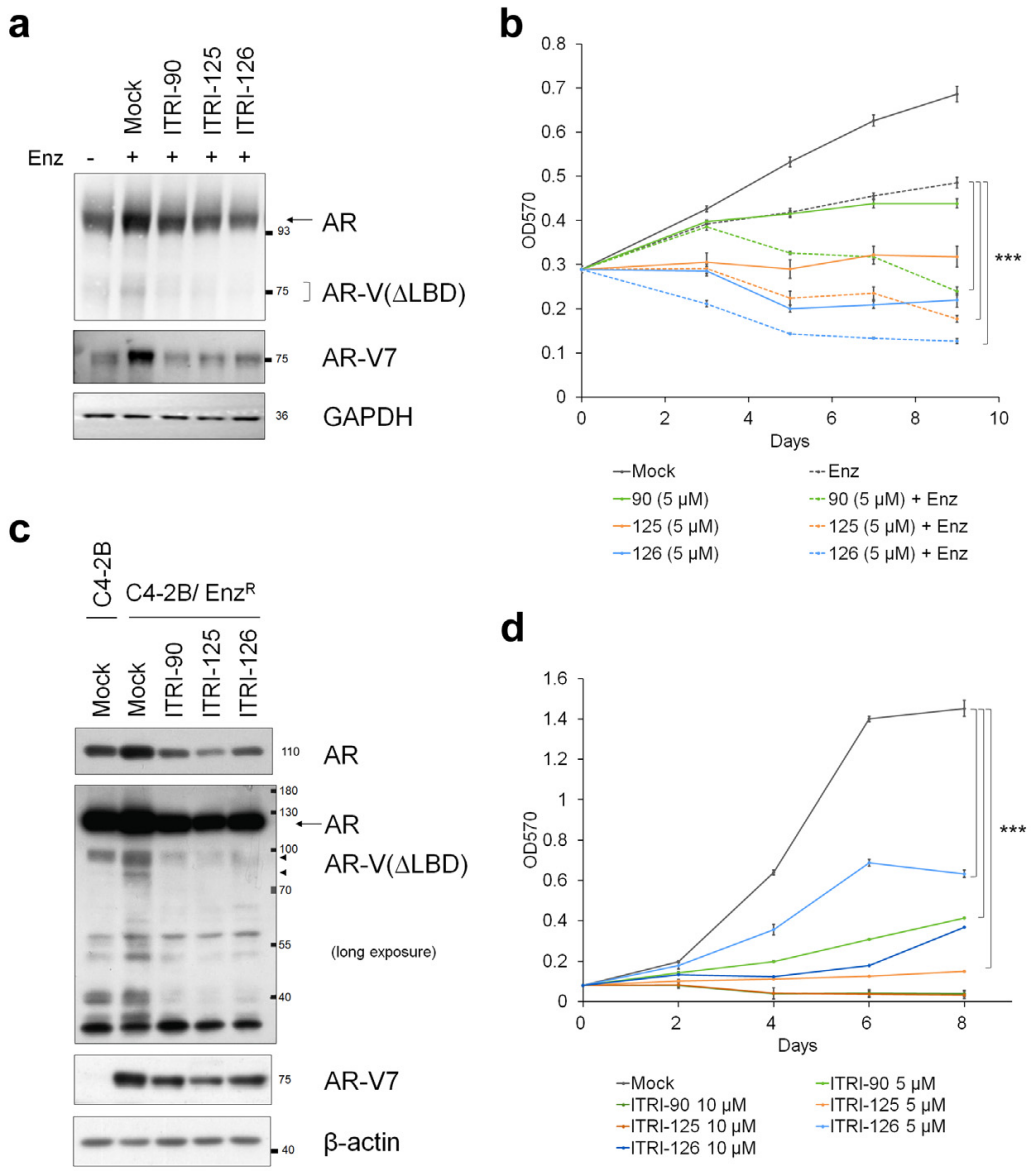
AR degraders effectively diminish enzalutamide-induced AR-V expression and inhibit enzalutamide-resistant cell growth. (a) VCaP cells were treated with 20 μM enzalutamide (Enz) for 2 days, followed by one-day treatment of mock or 10 μM PROTAC. Full-length AR and AR-V(ΔLBD) was detected by N-terminal AR antibody (top) or AR-V7 specific antibody (middle). (b) VCaP cells were treated with or without 20 μM enzalutamide for 1 day and subsequently treated with mock or 10 μM AR PROTAC. Cell proliferation was detected by MTT every 2 days after PROTAC treatment, up to 8 days. Asterisks indicate statistical significance between enzalutamide alone and the combined treatment groups (∗∗∗*p* < 0.001). (c) AR protein levels in C4-2B and C4-2B/Enz^R^ cells treated with mock or 10 μM PROTAC compounds for 1 day were detected by anti-N-terminal-AR and anti-AR-V7 antibodies. Arrowheads indicate possible C-terminal truncated AR-Vs up-regulated in C4-2B/Enz^R^ cells. (d) C4-2B/Enz^R^ cell growth upon PROTAC compounds treatment. The drugs were added to cells on day 0. Cell growth was monitored by MTT every 2 days up to 8 days. Asterisks indicate statistical significance between 5 μM drug treatment groups and mock (∗∗∗*p* < 0.001, two-tailed Student's t test).

Upon androgen deprivation or enzalutamide treatment, AR and AR-V7 expression is increased in VCaP cells and xenografts, while AR-V7 in particular, has been reported to drive cell proliferation under ADT condition.^19^^,^^20^ As we consistently observed the up-regulation of AR and AR-V7 in VCaP cells upon enzalutamide treatment, the AR degraders significantly reversed this induction (Fig. 6a). Furthermore, MTT assay showed that while enzalutamide treatment modestly inhibits VCaP proliferation, combined treatment of enzalutamide and ITRI-PROTACs drastically impaired the cell growth, suggesting that the AR-V7-dependent growth of enzalutamide treated CRPC cells can be effectively inhibited by ITRI AR degraders (Fig. 6b). Having demonstrated the efficacy of ITRI AR degraders on xenograft-derived enzalutamide resistant CRPC cells, we were also interested in an enzalutamide-resistant CRPC cell model derived *in vitro* by enzalutamide exposure. Long-term drug exposure of C4-2B cells resulted in C4-2B/Enz^R^ cells which displayed enzalutamide resistance as well as elevated AR-FL and AR-V7 (Fig. 6c).^7^^,^^8^ Of note, while AR-V7 specific antibody detected the overexpression of AR-V7, more than one species of C-terminus truncated AR with molecular weights ranging in 75–110 kDa were also detected by the N-terminal antibody, suggesting that multiple LBD-truncated AR-Vs are expressed in the C4-2B/Enz^R^ cells. Importantly, ITRI AR degraders were able to efficiently reduce both AR-FL and the truncated AR-Vs within one day of treatment (Fig. 6c). The proliferation of C4-2B/Enz^R^ cells was also significantly inhibited by the AR degraders (Fig. 6d). Together, we showed that the ITRI-PROTACs effectively degraded enzalutamide-induced AR-FL and truncated AR-V(ΔLBD) proteins in both short-term treated and long-term acquired resistant cells, thereby significantly impairing the enzalutamide-resistant cell growth.

### Antitumor activity of ITRI-PROTACs in enzalutamide-resistant CRPC xenograft model

Liver microsomal stability was first examined for the degraders against mouse liver microsomes by HPLC. All three compounds well tolerated the microsomes with the concentration remaining over 78% after 30 min reaction. The good microsomal stability was further echoed by *in vivo* pharmacokinetics (PK) profiles. Via intravenous administration, all compounds showed low plasma clearance rate (6.62 mL/min/kg for ITRI-90; 5.68 mL/min/kg for ITRI-125; 24.72 mL/min/kg for ITRI-126), while ITRI-90 and ITRI-125 achieved a better actual body exposure compared to ITRI-126 with the AUC/Dose being 2502.4 ± 49.2, 2839.9 ± 36.7 and 441.0 ± 37.8 h·ng/mL for 90, 125, and 126, respectively. Given that ITRI-125 showed shorter sustainability towards AR degradation compared to ITRI-90 and ITRI-126 *in vitro* (Fig. 2), we proceeded with ITRI-90 and ITRI-126 for further PK assessment of intraperitoneal (IP) and oral (PO) routing as well as assessment of antitumor efficacies.

**Fig. 7:**
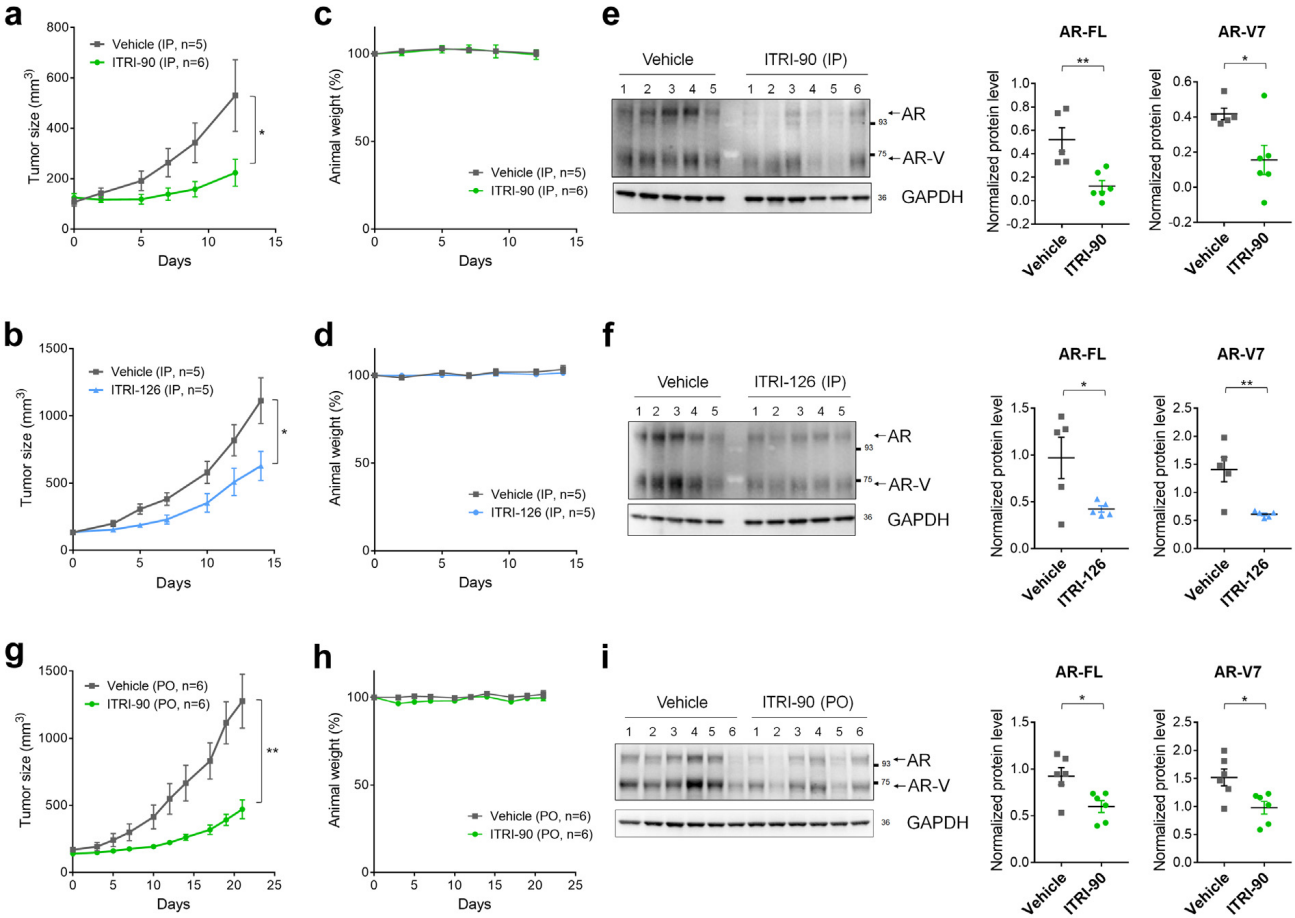
Efficacy of ITRI-90 and ITRI-126 in tumor regression of the CWR22Rv1 xenograft model. CWR22Rv1 tumor xenografts implanted in SCID mice were treated with ITRI-90 or ITRI-126 via IP injection (a–f) using 10 mg/kg once daily dosing, or with ITRI-90 orally (PO) using 100 mg/kg twice daily dosing (g–i). Animal number used in each group is as indicated. (a, b, g) Scatter plots of the tumor size; (c, d, h) corresponding animal weight monitored along the course of analyses. (e, f, i) Western blotting of AR and AR-V protein in the tumors collected at the final time points. The protein levels quantitated and normalized against GAPDH levels were plotted as indicated. Asterisks indicate statistical significance between the PROTAC treated groups and mock (∗*p* < 0.05; ∗∗*p* < 0.01, two-tailed Student's t test).

Using 10 mg/kg single IP administration, ITRI-90 and ITRI-126 showed C_max_ values of 3945 and 706 ng/mL, and AUC_last_ of 25510.9 and 2341.8 h·ng/mL, respectively (Table 1). Although the AUC/Dose of ITRI-126 is significantly lower than that of ITRI-90, given its superior *in vitro* potency, the total drug exposure for ITRI-126 via IP administration was estimated to be sufficient to exceed the *in vitro* IC_50_ dosage. The PK profiles of both compounds thus presented promising potentials for further antitumor assessment. CWR22Rv1 xenograft model, which exhibits primary resistance to both ADT and second-generation anti-androgen agents such as enzalutamide, and shows sensitivity only towards chemotherapy (Fig. S14), was used for the following animal studies without hormone manipulation. IP administration of ITRI-90 and ITRI-126 with 10 mg/kg dosage twice daily significantly impaired the tumor growth (Fig. 7a and b) without obvious toxicities as monitored by the animal body weight (Fig. 7c and d). Compared to vehicle-treated mice, ITRI-90 treated group reached an average tumor growth inhibition (TGI) of 76.64 ± 11.1% on day 12, and that of 49.8 ± 11.1% for ITRI-126 on day 14. Consistently, both treatments significantly decreased AR-FL and AR-V protein levels in tumors detected at the final time point (Fig. 7e and f).

**Table 1:** Pharmacokinetics profiles of ITRI-90 and ITRI-126 administrated via intraperitoneal injection.

	ITRI-90 (IP, 10 mg/kg)	ITRI-126 (IP, 10 mg/kg)
T_max_ (h)	1.5 ± 0.7	1.5 ± 0.5
C_max_ (ng/mL)	3945.0 ± 1138.4	706.0 ± 222.0
AUC_last_ (h⋅ng/mL)	25510.9 ± 13641.8	2341.8 ± 263.2
AUC/Dose (h⋅kg⋅ng/mL/mg)	2510.1 ± 1364.2	234.2 ± 26.3

AUC_last_, area-under-the-curve between 0 and 24 h; AUC/Dose, area-under-the-curve between 0 and 24 h per dosing; C_max_, maximum drug concentration; T_max_, the time takes to reach C_max_.

We next assessed the performance of compounds following oral administration. At 30 mg/kg single daily dosing, ITRI-90 showed C_max_ value of 7056 ng/mL and AUC_last_ value of 18,004 h⋅ng/mL. The estimated drug exposure well exceeded the *in vitro* IC_50_, indicating a potential promising oral bioavailability for ITRI-90. However, given the solubility limitation, ITRI-126 at its maximum dosage of 10 mg/kg on the other hand, exhibited a poorer oral PK profile with 95.7 ng/mL C_max_ value and 203.7 h·ng/mL AUC_last_ value, and a significantly lower oral bioavailability (4.6%) (Table 2). These data suggested that while both ITRI-90 and ITRI-126 showed good PK and antitumor efficacy via IP route, ITRI-90 has superior performance over ITRI-126 for PO routing. We next tested the anti-proliferation effect of orally administrated ITRI-90 using 100 mg/kg and twice daily dosing on the CWR22RV1 xenograft model without hormone ablation. Remarkably, significant tumor suppression with an average TGI of 71.73 ± 6.42% on day 21 was observed without obvious toxicity (Fig. 7g and h). Consistently, the protein levels of AR-FL and AR-Vs in the tumors were also reduced in ITRI-90 treated group (Fig. 7i). The antitumor specificity of ITRI-90 via AR targeting was further validated by its ineffectiveness towards AR-negative PC3 prostate cancer model (Fig. S15). These data demonstrated that ITRI-90 is an orally available degrader specifically targeting AR-FL and LBD-truncated AR-Vs, and that treatment of ITRI-90 alone shows promising antitumor efficacy in enzalutamide-resistant CRPC xenograft model.

**Table 2:** Pharmacokinetics profiles of ITRI-90 and ITRI-126 administrated via oral routing.

	ITRI-90 (PO, 30 mg/kg)	ITRI-126 (PO, 10 mg/kg)
T_max_ (h)	1.67 ± 0.58	0.7 ± 0.2
C_max_ (ng/mL)	7056.67 ± 2594.81	95.7 ± 47.1
AUC_last_ (h⋅ng/mL)	18004.47 ± 7899.87	203.7 ± 85.9
AUC/Dose (h⋅kg⋅ng/mL/mg)	600.13 ± 263.33	20.4 ± 8.6
BA (%)	23	4.6 ± 1.9

AUC_last_, area-under-the-curve between 0 and 24 h; AUC/Dose, area-under-the-curve between 0 and 24 h per dosing; BA, bioavailability; C_max_, maximum drug concentration; T_max_, the time takes to reach C_max_.

## Discussion

AR NTD containing the transactivation domain is the hub for interaction between AR and many proteins including basic transcriptional machinery and co-activators, and governs the transcriptional activities of all AR species. Sadar et al.^56^ reported the first development of AR NTD small molecule inhibitor that blocks both AR-FL and AR-V transcriptional activity. Since then, several inhibitors targeting AR NTD for CRPC such as EPI-001, sintokamides, and ralaniten have been developed.^44^^,^^45^^,^^57^ Although the first-in-class clinical trial for ralaniten-acetate (EPI-506) was terminated due to poor oral bioavailability,^46^ NTD inhibitor-mediated blockage of protein–protein interactions between AR and co-activators and subsequent inhibition of the AR signaling axis was demonstrated and the strategy remains attractive. Here, we offer an alternative strategy to target NTD. Given that AR NTD lacks stable three-dimensional structure which is considered to be a challenging target by conventional small molecule design, with the mechanism of POI-E3 ligase complex formation mediated by PROTAC, it is feasible for the proteins to be catalytically induced for degradation via a binding moiety less demanding for affinity and binding region requirement.

We presented PROTAC compounds targeting the AR NTD for degradation of both full length and LBD-truncated AR. ITRI-90, ITRI-125 and ITRI-126 which utilizes VHL and CRBN binding ligand, respectively, are valid PROTAC degraders that displayed strong degradation potencies on AR-FL and AR-V(ΔLBD) proteins via a ubiquitin-proteasome system. The degradation of AR attenuated DHT-induced AR transactivation, suppressed gene expression including AR-V7 specific targets, and resulted in efficient growth inhibition specific to cancer cells. The ability of degrade both AR-FL and AR-Vs in cells harboring AR mutation, alternative splicing, and amplification, as represented by the three cell lines used in this study, suggests that targeting the common N-terminal part of these variants for degradation is an efficient strategy to inhibit AR signaling-dependent CRPC cell growth. It should also be noted that targeting AR through DBD-binder-induced degradation such as the case of MTX-23,^39^ could potentially provide similar advantages like NTD degraders.

The three PROTAC compounds presented here share an identical AR binding moiety, and differ only in their linker length or the E3 ligase binding ligand. Interestingly, these compounds displayed different kinetics and potency for AR degradation *in vitro*, as well as different PK profiles in animal. ITRI-90 and ITRI-126, sharing the same linker, appeared to display longer duration of intracellular efficacy in AR degradation compared to ITRI-125, which contains two more atoms in the linker. Different from a traditional occupancy-based mechanism, PROTAC functions by catalytically recruiting enzyme cascade for POI degradation, hence has the ability to undergo multiple rounds of actions. The duration of PROTAC efficacy in cells may indicate the intracellular drug stability before clearance, thereby affecting target engagement. Strategies to improve intracellular PROTAC stability are inadequately addressed in this field thus far. We speculate that while linker length is crucial for degradation activity,^58^ a longer linker may be more vulnerable to cleavage in target cells. Given that persistent target degradation up to 96 h after drug washout has been reported for PROTAC compounds,^59^, ^60^, ^61^ we assume that ITRI-90 and ITRI-126 with a prolonged intracellular efficacy may have greater benefit for therapeutic purpose. On the other hand, while harboring CRBN binding ligand improves the *in vitro* potencies of protein degradation (DC_50_) and growth inhibition (IC_50_) for ITRI-126, presumably due to its lower molecular weight, the overall *in vivo* PK profile of this compound is inferior to ITRI-90 that utilizes VHL binding ligand. Although ITRI-90 showed higher DC_50_ compared to other reported PROTAC agents that are in nanomolar range, the therapeutic regimen for ITRI-90 required to suppress tumor growth in the xenograft model is in a reasonable range.

Despite widely recognized advantages of PROTAC degraders as a therapeutic modality, one aspect that remains challenging is the achievement of oral availability.^62^ We showed that ITRI-90 achieved an overall good PK profile via oral administration and strong antitumor efficacy as a monotherapy with 100 mg/kg twice daily dosing without any sign of toxicity. Optimizing the formulation for ITRI-90 oral bioavailability is anticipated to further improve its *in vivo* potencies. Moreover, unlike AR antagonist agents whose efficacy can be attenuated by high androgen levels, ITRI-90 by degrading the AR proteins, is sufficient to inhibit tumor growth even in high androgen environment, which is present in CRPC tumors resulting from intracrine steroidogenesis.^63^ Combined use of the ITRI-PROTAC and ADT or AR antagonist agents may provide further antitumor efficacy.

In summary, this preclinical study presented orally available ITRI-90 PROTAC that targets the AR N-terminal transactivation domain for induced degradation of all active, NTD-containing AR proteins including the clinical relevant AR-V7. ITRI-PROTAC compounds effectively impaired the tumor growth in a castration- and enzalutamide-resistant xenograft animal model. Given that sustained AR signaling attributed to the truncated AR variants or AR overexpression is the major mechanism underlying the therapy resistance in advanced prostate cancer, we anticipate that simultaneously degrading both AR-FL and constitutively active AR-Vs is an efficient alternative approach to intervene CRPC and overcome anti-androgen drug resistance.

## Contributors

C.-L.H. and L.-Y.W. designed the research, performed data analysis and interpretation; C.-L.H., H.-J.K. and L.-Y.W. wrote the paper; C.-L.H., H.-H.L., and C.-W.F. performed chemical design and synthesis; H.-H.Y. performed qPCR, ubiquitination, caspase and MTT analysis; L.-Y.W. performed luciferase assay; T.-L.H. and Y.-C.L. performed pharmacokinetics studies; Z.-K.K., and M.-R.J. performed animal studies and Western blot analysis; C.-L.H., C.-S.H. and L.-Y.W. acquired funding; C.-S.H. and H.-C.H. provided critical consultations. C.-L.H. and L.-Y.W. verified the underlying data. All authors read and approved the final manuscript.

## Data sharing statement

All data supporting the findings of this study are available from the corresponding author upon reasonable request.

## Declaration of interests

All authors have declared that no competing interest exists.

## Appendix A Supplementary data

Supplemental Western Blots

Supplemental Figures 1–15 and Tables 1, 2

Caption for Supplementary Material does not contain scientific information for the reseach article itself, and is considered to be dispensable.

Reagent Validation

## References

[1] Sung H., Ferlay J., Siegel R.L. (2021). Global Cancer Statistics 2020: GLOBOCAN estimates of incidence and mortality worldwide for 36 cancers in 185 countries. CA Cancer J Clin.

[2] Nakazawa M., Paller C., Kyprianou N. (2017). Mechanisms of therapeutic resistance in prostate cancer. Curr Oncol Rep.

[3] Carlin B.I., Andriole G.L. (2000). The natural history, skeletal complications, and management of bone metastases in patients with prostate carcinoma. Cancer.

[4] Crona D.J., Milowsky M.I., Whang Y.E. (2015). Androgen receptor targeting drugs in castration-resistant prostate cancer and mechanisms of resistance. Clin Pharmacol Ther.

[5] Moilanen A.M., Riikonen R., Oksala R. (2015). Discovery of ODM-201, a new-generation androgen receptor inhibitor targeting resistance mechanisms to androgen signaling-directed prostate cancer therapies. Sci Rep.

[6] Clegg N.J., Wongvipat J., Joseph J.D. (2012). ARN-509: a novel antiandrogen for prostate cancer treatment. Cancer Res.

[7] Liu C., Yang J.C., Armstrong C.M. (2019). AKR1C3 promotes AR-V7 protein stabilization and confers resistance to AR-targeted therapies in advanced prostate cancer. Mol Cancer Ther.

[8] Zhao J., Ning S., Lou W. (2020). Cross-resistance among next-generation antiandrogen drugs through the AKR1C3/AR-V7 axis in advanced prostate cancer. Mol Cancer Ther.

[9] Schmidt K.T., Huitema A.D.R., Chau C.H., Figg W.D. (2021). Resistance to second-generation androgen receptor antagonists in prostate cancer. Nat Rev Urol.

[10] Prekovic S., van den Broeck T., Linder S. (2018). Molecular underpinnings of enzalutamide resistance. Endocr Relat Cancer.

[11] Buttigliero C., Tucci M., Bertaglia V. (2015). Understanding and overcoming the mechanisms of primary and acquired resistance to abiraterone and enzalutamide in castration resistant prostate cancer. Cancer Treat Rev.

[12] Antonarakis E.S., Lu C., Luber B. (2017). Clinical significance of androgen receptor splice variant-7 mRNA detection in circulating tumor cells of men with metastatic castration-resistant prostate cancer treated with first- and second-line abiraterone and enzalutamide. J Clin Oncol.

[13] Sharp A., Coleman I., Yuan W. (2019). Androgen receptor splice variant-7 expression emerges with castration resistance in prostate cancer. J Clin Invest.

[14] Hu R., Dunn T.A., Wei S. (2009). Ligand-independent androgen receptor variants derived from splicing of cryptic exons signify hormone-refractory prostate cancer. Cancer Res.

[15] Antonarakis E.S., Armstrong A.J., Dehm S.M., Luo J. (2016). Androgen receptor variant-driven prostate cancer: clinical implications and therapeutic targeting. Prostate Cancer Prostatic Dis.

[16] Maughan B.L., Antonarakis E.S. (2015). Clinical relevance of androgen receptor splice variants in castration-resistant prostate cancer. Curr Treat Options Oncol.

[17] Wadosky K.M., Koochekpour S. (2017). Androgen receptor splice variants and prostate cancer: from bench to bedside. Oncotarget.

[18] Antonarakis E.S., Lu C., Wang H. (2014). AR-V7 and resistance to enzalutamide and abiraterone in prostate cancer. N Engl J Med.

[19] Liu L.L., Xie N., Sun S., Plymate S., Mostaghel E., Dong X. (2014). Mechanisms of the androgen receptor splicing in prostate cancer cells. Oncogene.

[20] Yu Z., Chen S., Sowalsky A.G. (2014). Rapid induction of androgen receptor splice variants by androgen deprivation in prostate cancer. Clin Cancer Res.

[21] Hu R., Lu C., Mostaghel E.A. (2012). Distinct transcriptional programs mediated by the ligand-dependent full-length androgen receptor and its splice variants in castration-resistant prostate cancer. Cancer Res.

[22] Sharma N.L., Massie C.E., Ramos-Montoya A. (2013). The androgen receptor induces a distinct transcriptional program in castration-resistant prostate cancer in man. Cancer Cell.

[23] Guo Z., Yang X., Sun F. (2009). A novel androgen receptor splice variant is up-regulated during prostate cancer progression and promotes androgen depletion-resistant growth. Cancer Res.

[24] Li Y., Chan S.C., Brand L.J., Hwang T.H., Silverstein K.A., Dehm S.M. (2013). Androgen receptor splice variants mediate enzalutamide resistance in castration-resistant prostate cancer cell lines. Cancer Res.

[25] Chen Z., Wu D., Thomas-Ahner J.M. (2018). Diverse AR-V7 cistromes in castration-resistant prostate cancer are governed by HoxB13. Proc Natl Acad Sci U S A.

[26] Robinson D., Van Allen E.M., Wu Y.M. (2015). Integrative clinical genomics of advanced prostate cancer. Cell.

[27] Cancer Genome Atlas Research Network (2015). The molecular taxonomy of primary prostate cancer. Cell.

[28] Kohli M., Ho Y., Hillman D.W. (2017). Androgen receptor variant AR-V9 is coexpressed with AR-V7 in prostate cancer metastases and predicts abiraterone resistance. Clin Cancer Res.

[29] Kallio H.M.L., Hieta R., Latonen L. (2018). Constitutively active androgen receptor splice variants AR-V3, AR-V7 and AR-V9 are co-expressed in castration-resistant prostate cancer metastases. Br J Cancer.

[30] Qi S.M., Dong J., Xu Z.Y., Cheng X.D., Zhang W.D., Qin J.J. (2021). PROTAC: an effective targeted protein degradation strategy for cancer therapy. Front Pharmacol.

[31] Mullard A. (2019). First targeted protein degrader hits the clinic. Nat Rev Drug Discov.

[32] Snyder L.B., Neklesa T.K., Chen X. (2021). Abstract 43: discovery of ARV-110, a first in class androgen receptor degrading PROTAC for the treatment of men with metastatic castration resistant prostate cancer. Cancer Res.

[33] Salami J., Alabi S., Willard R.R. (2018). Androgen receptor degradation by the proteolysis-targeting chimera ARCC-4 outperforms enzalutamide in cellular models of prostate cancer drug resistance. Commun Biol.

[34] Takwale A.D., Jo S.H., Jeon Y.U. (2020). Design and characterization of cereblon-mediated androgen receptor proteolysis-targeting chimeras. Eur J Med Chem.

[35] Han X., Wang C., Qin C. (2019). Discovery of ARD-69 as a highly potent proteolysis targeting chimera (PROTAC) degrader of androgen receptor (AR) for the treatment of prostate cancer. J Med Chem.

[36] Han X., Zhao L., Xiang W. (2019). Discovery of highly potent and efficient PROTAC degraders of androgen receptor (AR) by employing weak binding affinity VHL E3 ligase ligands. J Med Chem.

[37] Kregel S., Wang C., Han X. (2020). Androgen receptor degraders overcome common resistance mechanisms developed during prostate cancer treatment. Neoplasia.

[38] Han X., Zhao L., Xiang W. (2021). Strategies toward discovery of potent and orally bioavailable proteolysis targeting chimera degraders of androgen receptor for the treatment of prostate cancer. J Med Chem.

[39] Lee G.T., Nagaya N., Desantis J. (2021). Effects of MTX-23, a novel PROTAC of androgen receptor splice variant-7 and androgen receptor, on CRPC resistant to second-line antiandrogen therapy. Mol Cancer Ther.

[40] Annis D.A., Nickbarg E., Yang X., Ziebell M.R., Whitehurst C.E. (2007). Affinity selection-mass spectrometry screening techniques for small molecule drug discovery. Curr Opin Chem Biol.

[41] Tsai H.C., Boucher D.L., Martinez A., Tepper C.G., Kung H.J. (2012). Modeling truncated AR expression in a natural androgen responsive environment and identification of RHOB as a direct transcriptional target. PLoS One.

[42] Boucher D.L. (2008).

[43] Shi X.B., Ma A.H., Xia L., Kung H.J., de Vere White R.W. (2002). Functional analysis of 44 mutant androgen receptors from human prostate cancer. Cancer Res.

[44] Andersen R.J., Mawji N.R., Wang J. (2010). Regression of castrate-recurrent prostate cancer by a small-molecule inhibitor of the amino-terminus domain of the androgen receptor. Cancer Cell.

[45] Leung J.K., Tam T., Wang J., Sadar M.D. (2021). Isolation and characterization of castration-resistant prostate cancer LNCaP95 clones. Hum Cell.

[46] Maurice-Dror C., Le Moigne R., Vaishampayan U. (2022). A phase 1 study to assess the safety, pharmacokinetics, and anti-tumor activity of the androgen receptor n-terminal domain inhibitor epi-506 in patients with metastatic castration-resistant prostate cancer. Invest New Drugs.

[47] Liu C., Lou W., Zhu Y. (2014). Niclosamide inhibits androgen receptor variants expression and overcomes enzalutamide resistance in castration-resistant prostate cancer. Clin Cancer Res.

[48] Bradbury R.H., Acton D.G., Broadbent N.L. (2013). Discovery of AZD3514, a small-molecule androgen receptor downregulator for treatment of advanced prostate cancer. Bioorg Med Chem Lett.

[49] Loddick S.A., Ross S.J., Thomason A.G. (2013). AZD3514: a small molecule that modulates androgen receptor signaling and function in vitro and in vivo. Mol Cancer Ther.

[50] Buckley D.L., Van Molle I., Gareiss P.C. (2012). Targeting the von Hippel-Lindau E3 ubiquitin ligase using small molecules to disrupt the VHL/HIF-1alpha interaction. J Am Chem Soc.

[51] Bartlett J.B., Dredge K., Dalgleish A.G. (2004). The evolution of thalidomide and its IMiD derivatives as anticancer agents. Nat Rev Cancer.

[52] Marcias G., Erdmann E., Lapouge G. (2010). Identification of novel truncated androgen receptor (AR) mutants including unreported pre-mRNA splicing variants in the 22Rv1 hormone-refractory prostate cancer (PCa) cell line. Hum Mutat.

[53] Wach S., Taubert H., Cronauer M. (2020). Role of androgen receptor splice variants, their clinical relevance and treatment options. World J Urol.

[54] Lin H.K., Altuwaijri S., Lin W.J., Kan P.Y., Collins L.L., Chang C. (2002). Proteasome activity is required for androgen receptor transcriptional activity via regulation of androgen receptor nuclear translocation and interaction with coregulators in prostate cancer cells. J Biol Chem.

[55] Zhou X., Han S., Wilder-Romans K. (2020). Neddylation inactivation represses androgen receptor transcription and inhibits growth, survival and invasion of prostate cancer cells. Neoplasia.

[56] Sadar M.D., Williams D.E., Mawji N.R. (2008). Sintokamides A to E, chlorinated peptides from the sponge Dysidea sp. that inhibit transactivation of the N-terminus of the androgen receptor in prostate cancer cells. Org Lett.

[57] Banuelos C.A., Tavakoli I., Tien A.H. (2016). Sintokamide A is a novel antagonist of androgen receptor that uniquely binds activation function-1 in its amino-terminal domain. J Biol Chem.

[58] Cyrus K., Wehenkel M., Choi E.Y. (2011). Impact of linker length on the activity of PROTACs. Mol Biosyst.

[59] Smith B.E., Wang S.L., Jaime-Figueroa S. (2019). Differential PROTAC substrate specificity dictated by orientation of recruited E3 ligase. Nat Commun.

[60] Burslem G.M., Smith B.E., Lai A.C. (2018). The advantages of targeted protein degradation over inhibition: an RTK case study. Cell Chem Biol.

[61] Steinebach C., Ng Y.L.D., Sosic I. (2020). Systematic exploration of different E3 ubiquitin ligases: an approach towards potent and selective CDK6 degraders. Chem Sci.

[62] Pike A., Williamson B., Harlfinger S., Martin S., McGinnity D.F. (2020). Optimising proteolysis-targeting chimeras (PROTACs) for oral drug delivery: a drug metabolism and pharmacokinetics perspective. Drug Discov Today.

[63] Montgomery R.B., Mostaghel E.A., Vessella R. (2008). Maintenance of intratumoral androgens in metastatic prostate cancer: a mechanism for castration-resistant tumor growth. Cancer Res.

