# Supplementary figures and images for: Targeting androgen receptor and the variants by an orally bioavailable Proteolysis Targeting Chimeras compound in castration resistant prostate cancer
Source: eBioMedicine. 2023 Mar 7;90:104500. doi: 10.1016/j.ebiom.2023.104500 (PMC10011747; doi:10.1016/j.ebiom.2023.104500)

Figure 1B

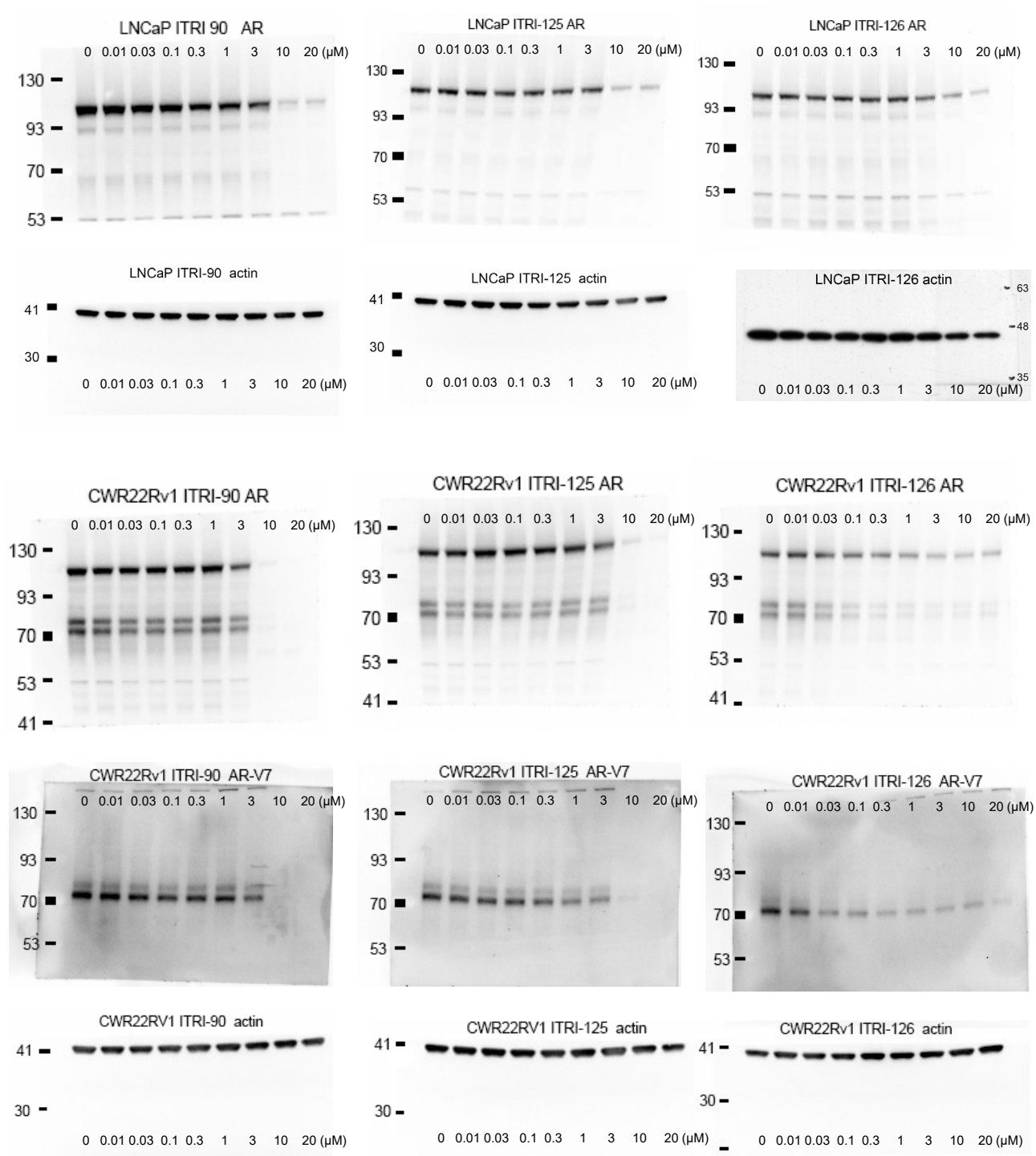

### Figure 1B

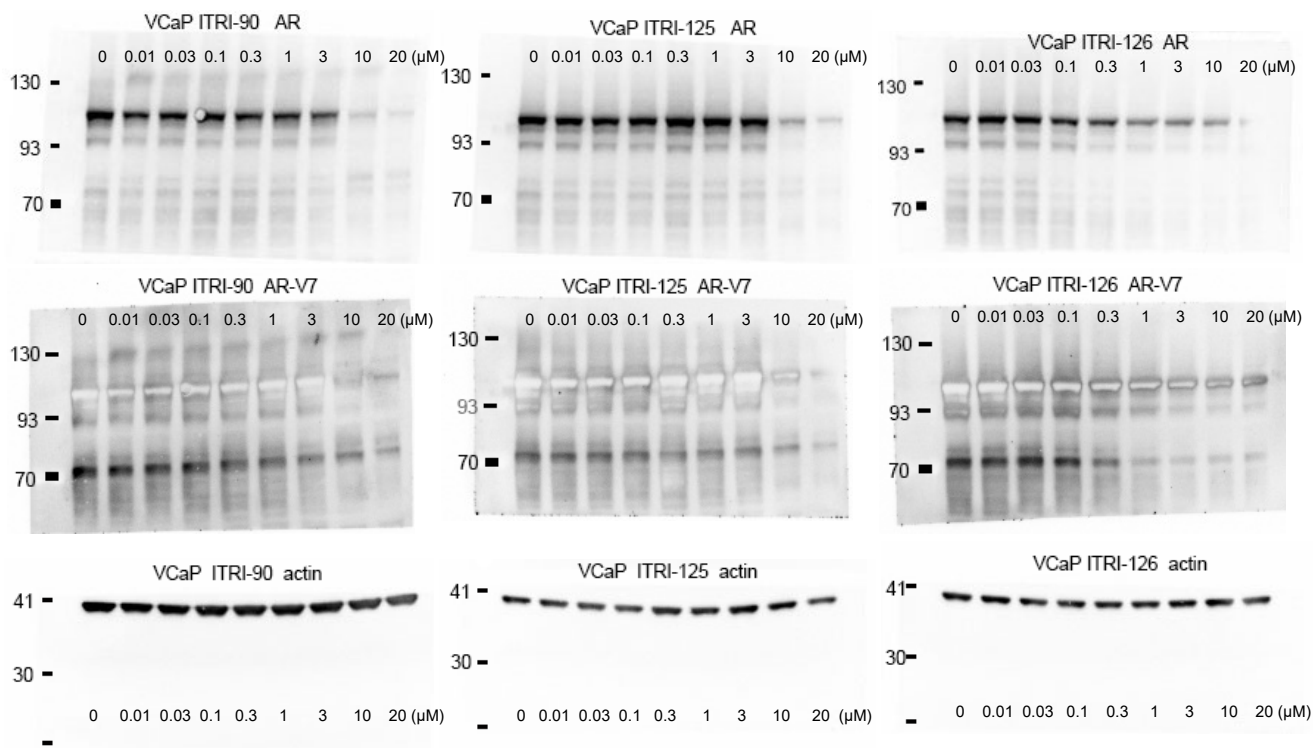

Figure 2

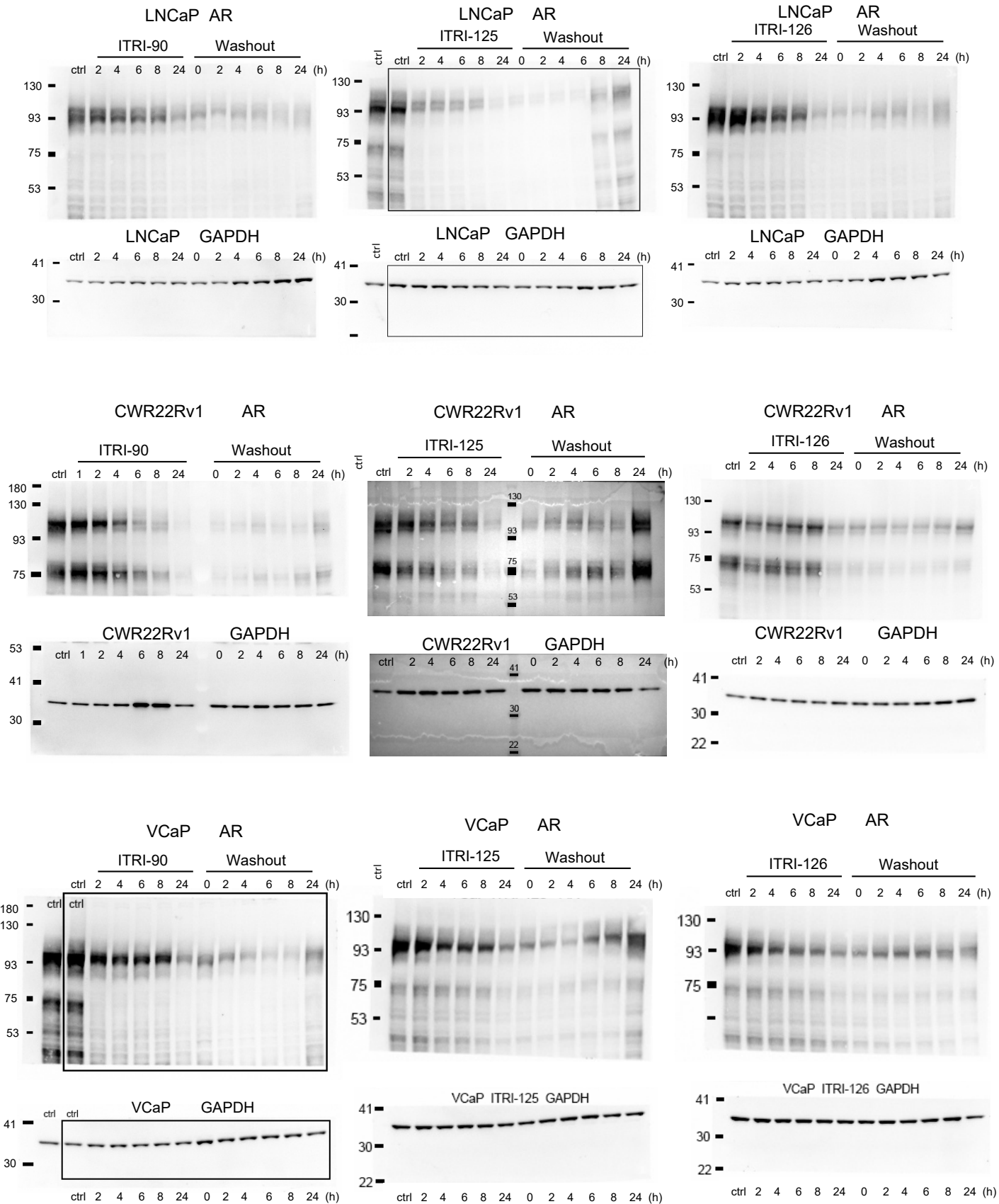

Figure 3

**a**

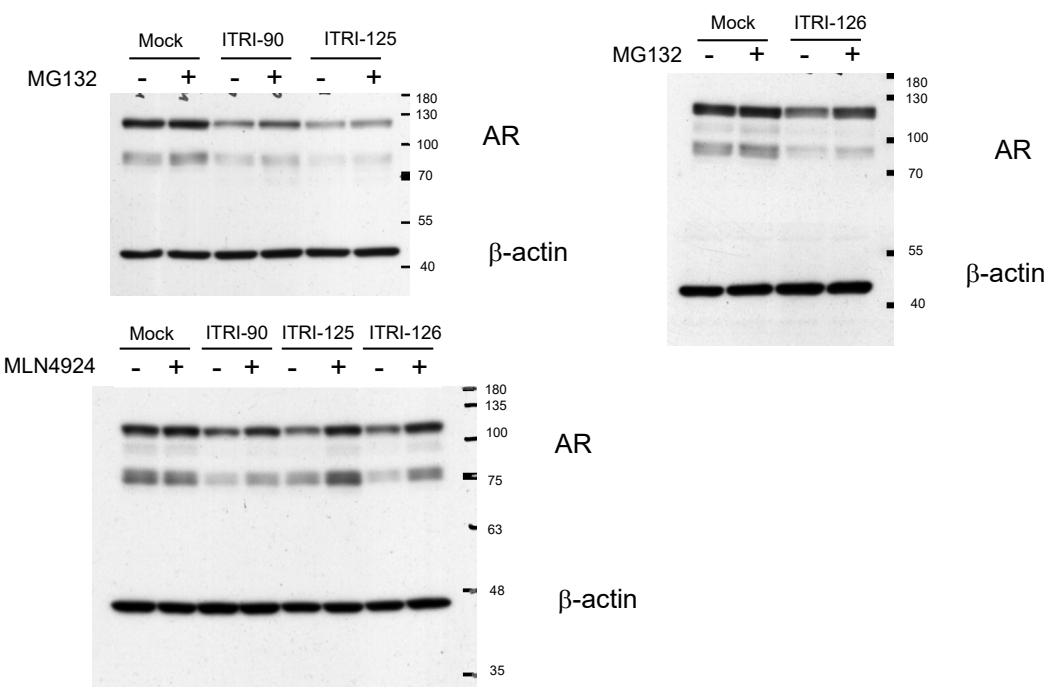

**b**

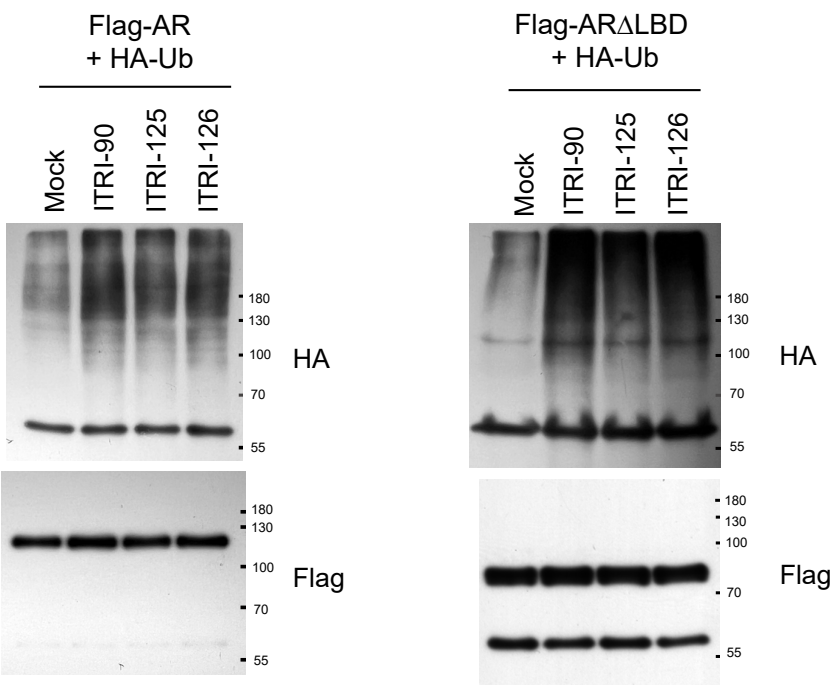

Figure 6

a

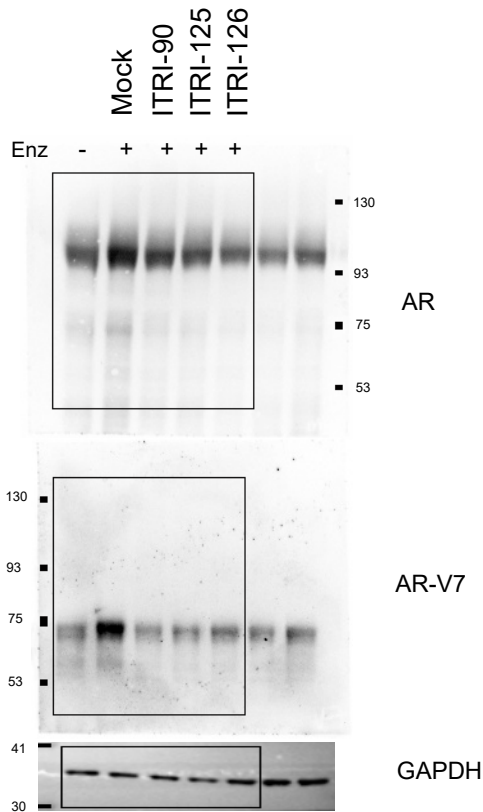

c

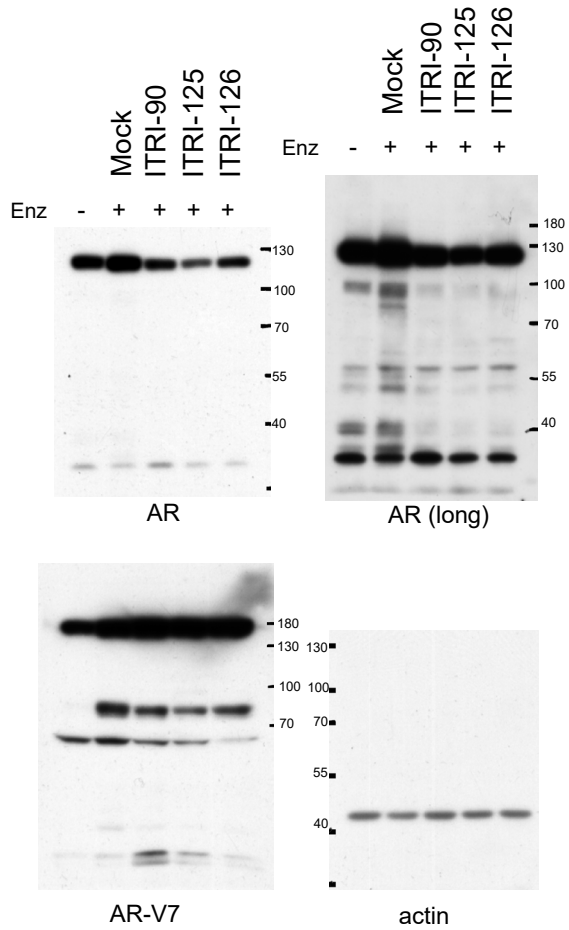

Figure 7

c

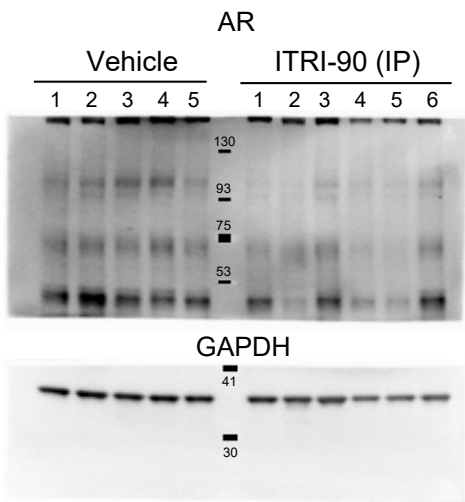

f

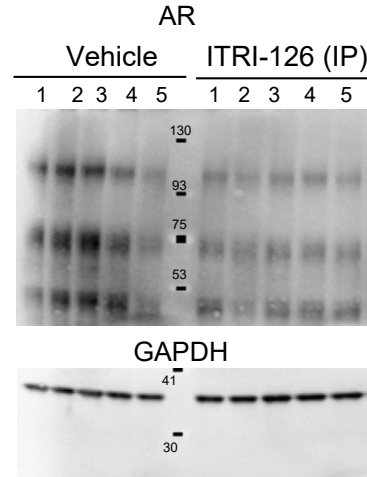

i

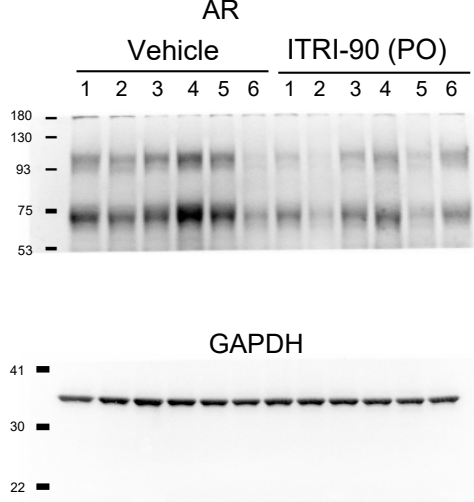

Figure S10

**b**

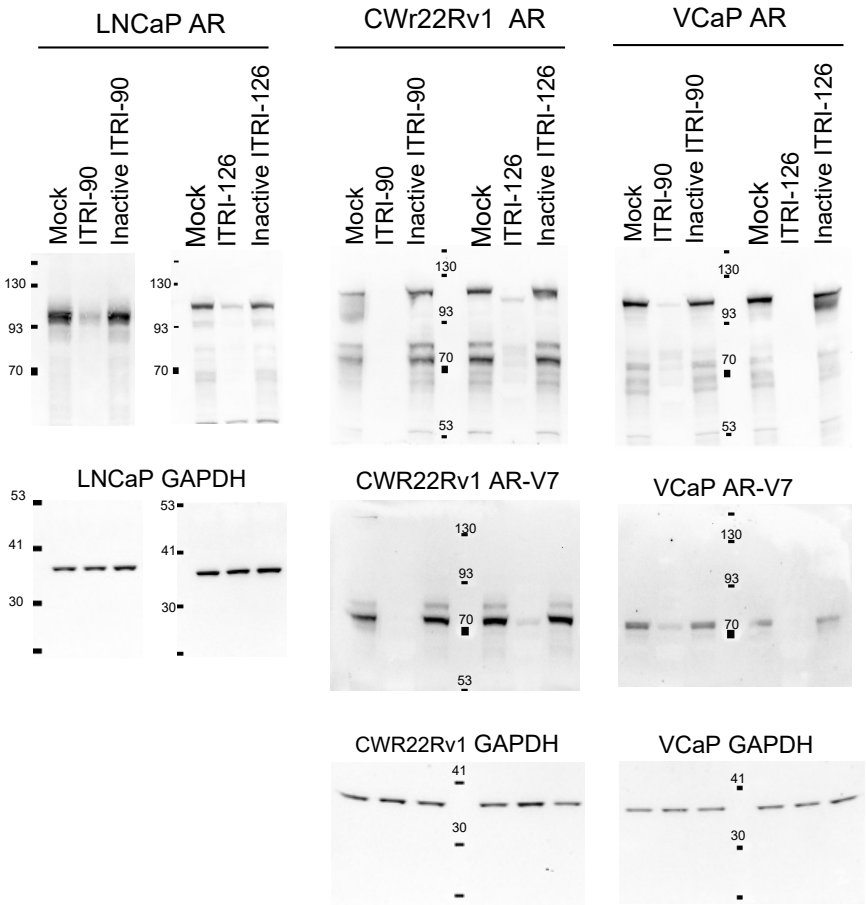

**c**

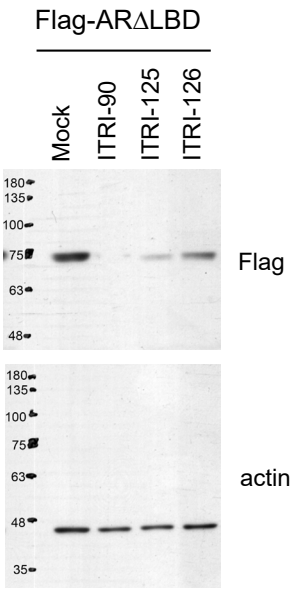

Figure S13

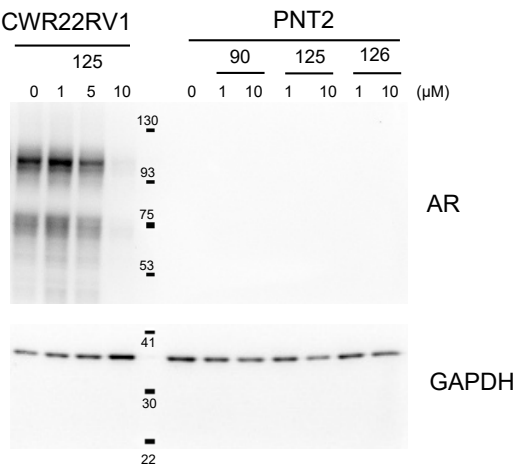

Figure S15

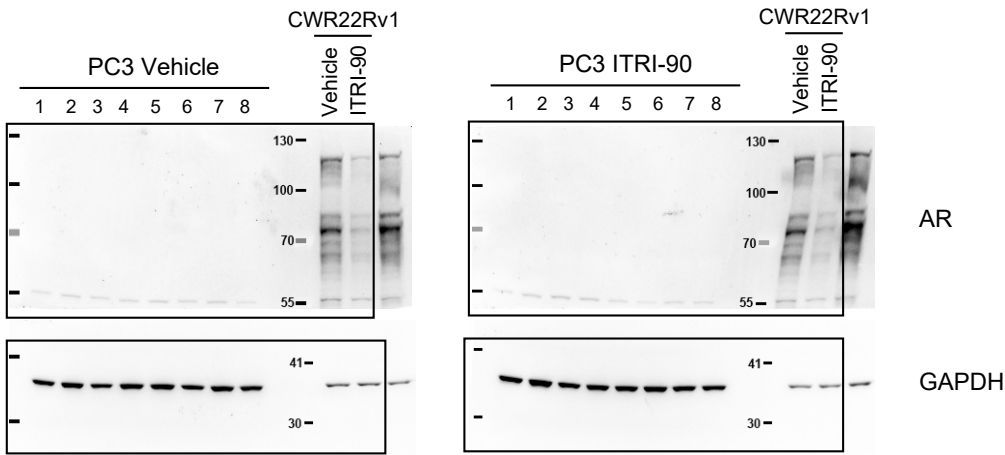

Supplement: Supplemental Western Blots [file mmc1.pdf]
