# Supplementary material for: Targeting androgen receptor and the variants by an orally bioavailable Proteolysis Targeting Chimeras compound in castration resistant prostate cancer
Source: eBioMedicine. 2023 Mar 7;90:104500. doi: 10.1016/j.ebiom.2023.104500 (PMC10011747; doi:10.1016/j.ebiom.2023.104500)
Supplement: Supplemental Figures 1–15 and Tables 1, 2 [file mmc2.pdf]

Supplementary Material for:

Targeting androgen receptor and the variants by an orally bioavailable Proteolysis Targeting Chimeras compound in castration resistant prostate cancer

Chiu-Lien Hung[1], Hao-Hsuan Liu[1], Chih-Wei Fu[1], Hsun-Hao Yeh[2], Tsan-Lin Hu[1], Zong-Keng Kuo[1], Yu-Chin Lin[1], Mei-Ru Jhang[1], Chrong-Shiong Hwang[1], Hung-Chih Hsu[3,4], Hsing-Jien Kung[5,6,7], Ling-Yu Wang[2,3,*]

[1] Department of Preclinical Drug Discovery Technology, Biomedical Technology and Devices Research Labs, Industrial Technology Research Institute, Hsinchu 31040, Taiwan.

[2] Department of Biochemistry and Molecular Biology, Chang Gung University, Taoyuan 33302, Taiwan.

[3] Division of Hematology-Oncology, Chang Gung Memorial Hospital at Linkou, Taoyuan 33305, Taiwan.

[4] College of Medicine, Chang Gung University, Taoyuan 33305, Taiwan.

[5] Institute of Molecular and Genomic Medicine, National Health Research Institutes, Zhunan, Miaoli County 35053, Taiwan.

[6] Research Center of Cancer Translational Medicine, Taipei Medical University, Taipei 11031, Taiwan.

[7] Ph.D. Program for Cancer Biology and Drug Discovery, College of Medical Science and Technology, Taipei Medical University, Taipei 11031, Taiwan.

* Corresponding author: Ling-Yu Wang
Department of Biochemistry and Molecular Biology, Chang Gung University
No.259, Wenhua 1st Rd., Guishan District.,
Taoyuan City 33302, Taiwan
+886-3-211-8800 ext.3989
lywang@mail.cgu.edu.tw

Table of Contents

Figure S1. ^{1}H-NMR spectra of compound 3 and compound 3a.
Figure S2. ^{1}H-NMR spectra of ITRI-90, ITRI-125 and ITRI-126.
Figure S3. ^{1}H-NMR spectra of inactive ITRI-90 and inactive ITRI-126
Figure S4. ^{13}C-NMR spectra of ITRI-90, ITRI-125 and ITRI-126.
Figure S5. ^{13}C-NMR spectra of inactive ITRI-90 and inactive ITRI-126.

Figure S6. HRMS spectra of ITRI-90, ITRI-125 and ITRI-126.
Figure S7. HRMS spectra of inactive ITRI-90 and inactive ITRI-126.
Figure S8. HPLC spectra of ITRI-90, ITRI-125 and ITRI-126.
Figure S9. HPLC spectra of inactive ITRI-90 and inactive ITRI-126.
Figure S10. Verification of PROTAC-induced AR and AR-V7 protein degradation.
Figure S11. Comparison of AR target inhibition by ITRI-PROTAC and enzalutamide.
Figure S12. Caspase 3/7 activity of LNCaP and CWR22Rv1 cells upon ITRI-126 treatment.
Figure S13. Viability of normal prostate epithelial PNT2 cells treated with ITRI-PROTAC compounds.
Figure S14. Tumor growth inhibition upon enzalutamide or docetaxel treatment in CWR22Rv1 xenograft model.
Figure S15. ITRI-90 is ineffective for AR-negative PC3 cells.
Table S1. Relative affinity binding, *in vitro* potencies and *in vitro* stability of candidate compounds.
Table S2. Primers used in qRT-PCR analysis

a

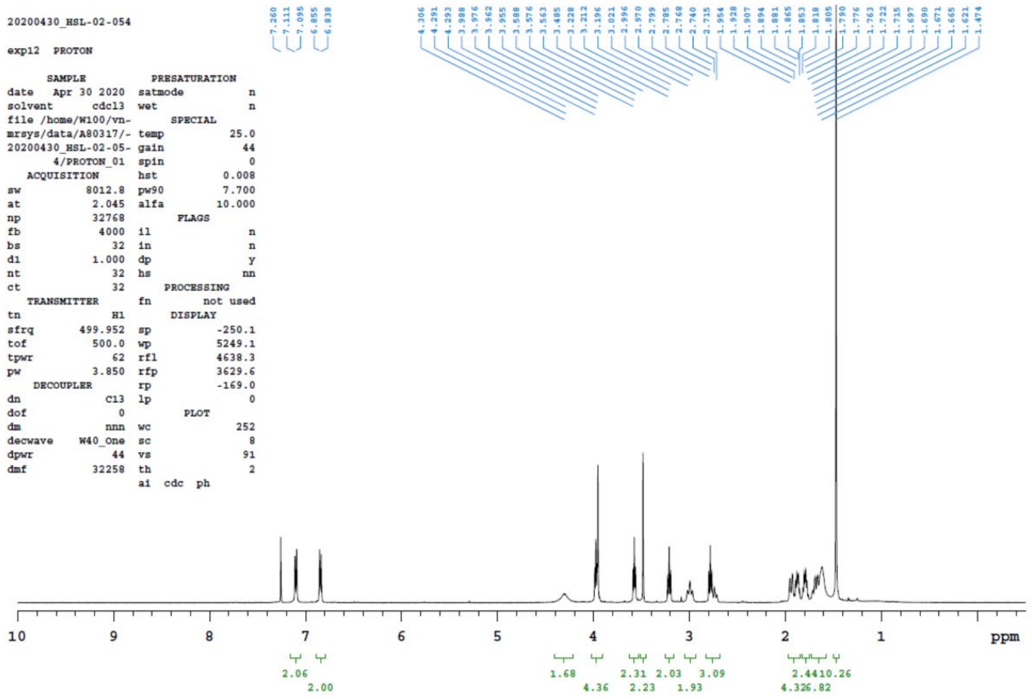

b

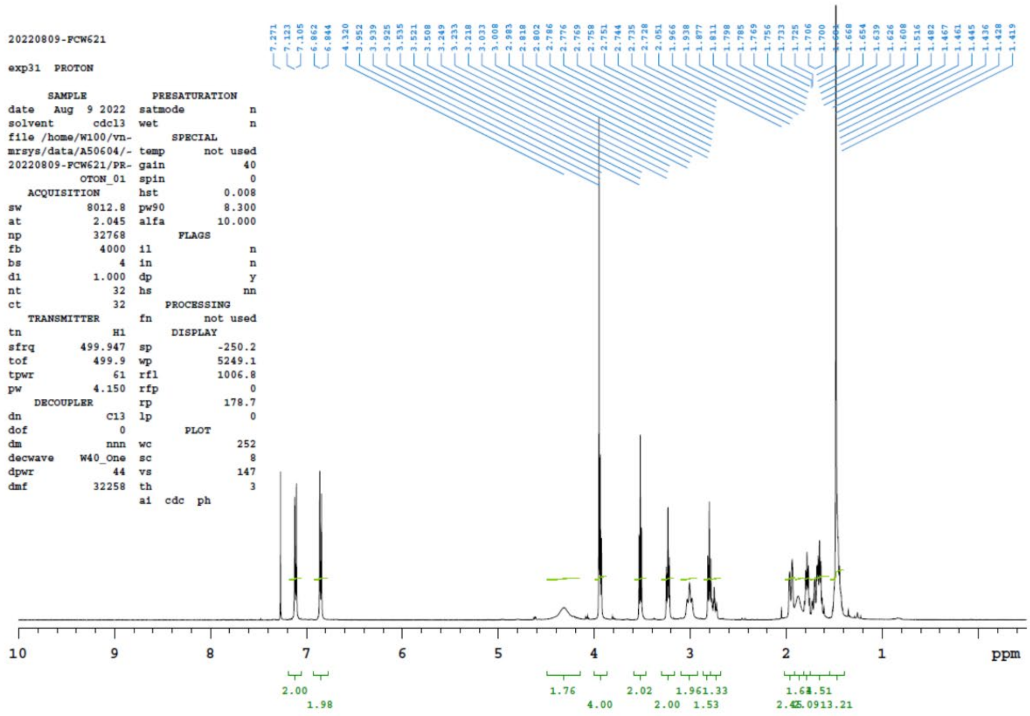

Figure S1. ^{1}H-NMR spectra of compound 3 (a) and compound 3a (b).

a

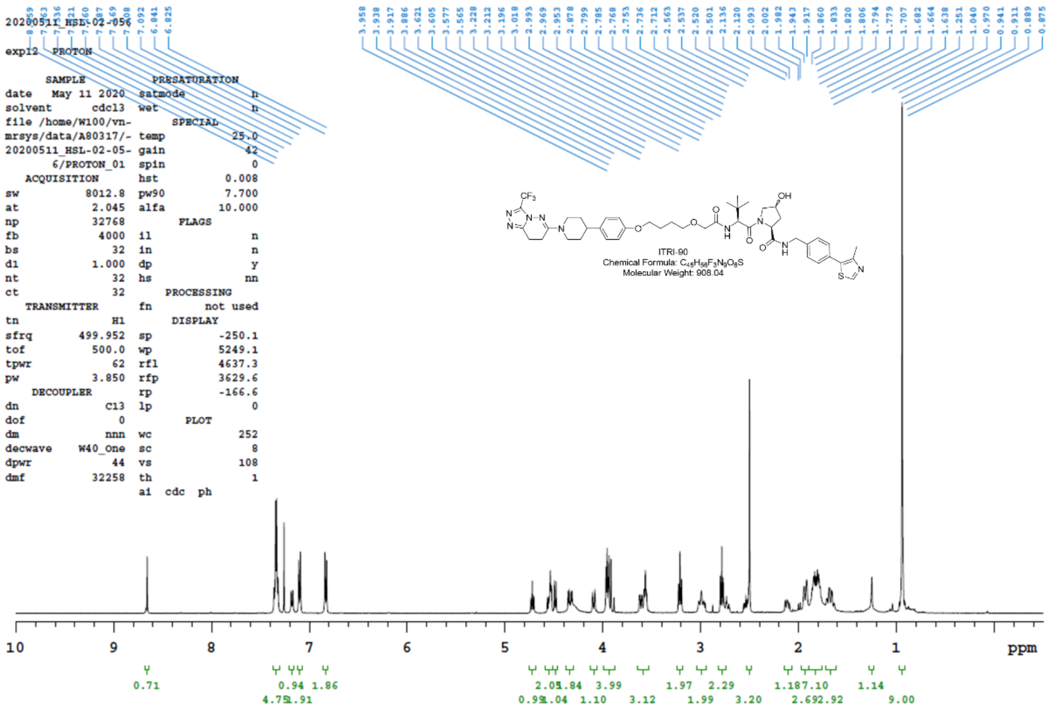
20200511_HSL-02-056
exp12 PROTON
SAMPLE
date May 11 2020
solvent cdcl3
file /home/W100/vn-mrsys/data/A80317/-20200511_HSL-02-05-6/PROTON_01
ACQUISITION
sw 8012.8
at 2.045
np 32768
fb 4000
bs 32
d1 1.000
nt 32
ct 32
TRANSMITTER
tn H1
sfrq 499.952
tof 500.0
tpwr 62
pw 3.850
DECOUPLER
dn C13
dof 0
dm nnn
decwave W40_One
dpwr 44
dmf 32258
PRESATURATION
satmode n
wet n
SPECIAL
temp 25.0
gain 42
spin 0
hst 0.008
pw90 7.700
alfa 10.000
FLAGS
il n
in n
dp y
hs nn
PROCESSING
fn not used
DISPLAY
sp -250.1
wp 5249.1
rfl 4637.3
rfp 3629.6
rp -166.6
lp 0
PLOT
wc 252
sc 8
vs 108
th 1
ai cdc ph
ITRI-90
Chemical Formula: C45H55F3N8O6S
Molecular Weight: 908.04
ppm

b

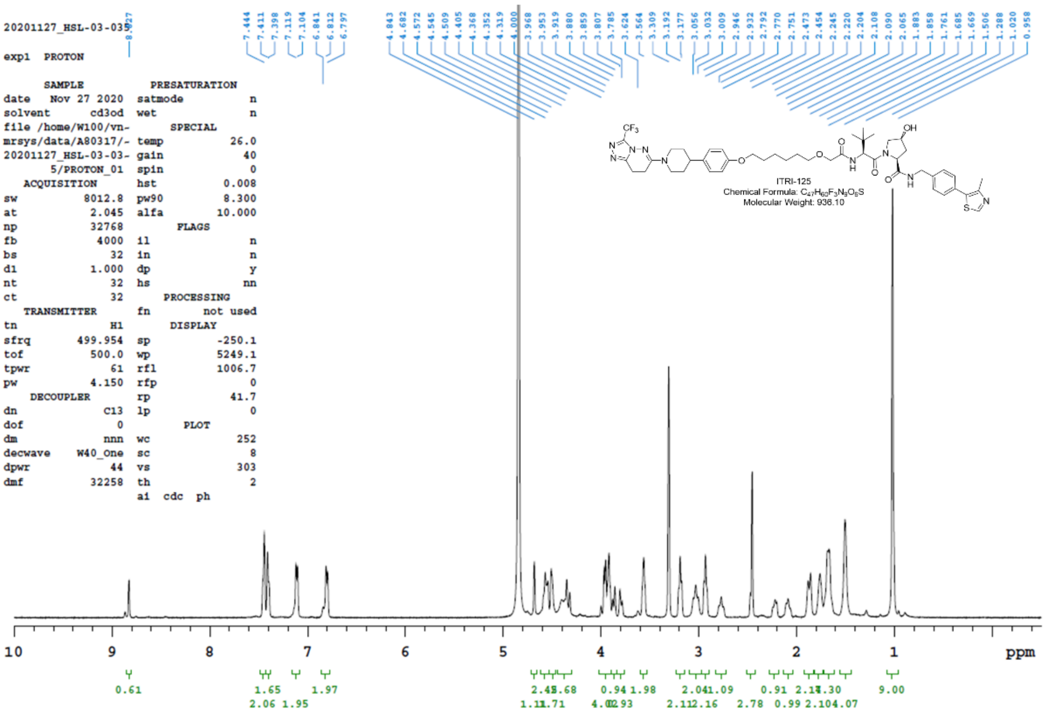
20201127_HSL-03-035
exp1 PROTON
SAMPLE
date Nov 27 2020
solvent cd3od
file /home/W100/vn-mrsys/data/A80317/-20201127_HSL-03-03-5/PROTON_01
ACQUISITION
sw 8012.8
at 2.045
np 32768
fb 4000
bs 32
d1 1.000
nt 32
ct 32
TRANSMITTER
tn H1
sfrq 499.954
tof 500.0
tpwr 61
pw 4.150
DECOUPLER
dn C13
dof 0
dm nnn
decwave W40_One
dpwr 44
dmf 32258
PRESATURATION
satmode n
wet n
SPECIAL
temp 26.0
gain 40
spin 0
hst 0.008
pw90 8.300
alfa 10.000
FLAGS
il n
in n
dp y
hs nn
PROCESSING
fn not used
DISPLAY
sp -250.1
wp 5249.1
rfl 1006.7
rfp 0
rp 41.7
lp 0
PLOT
wc 252
sc 8
vs 303
th 2
ai cdc ph
ITRI-125
Chemical Formula: C47H60F3N9O6S
Molecular Weight: 936.10
ppm

c

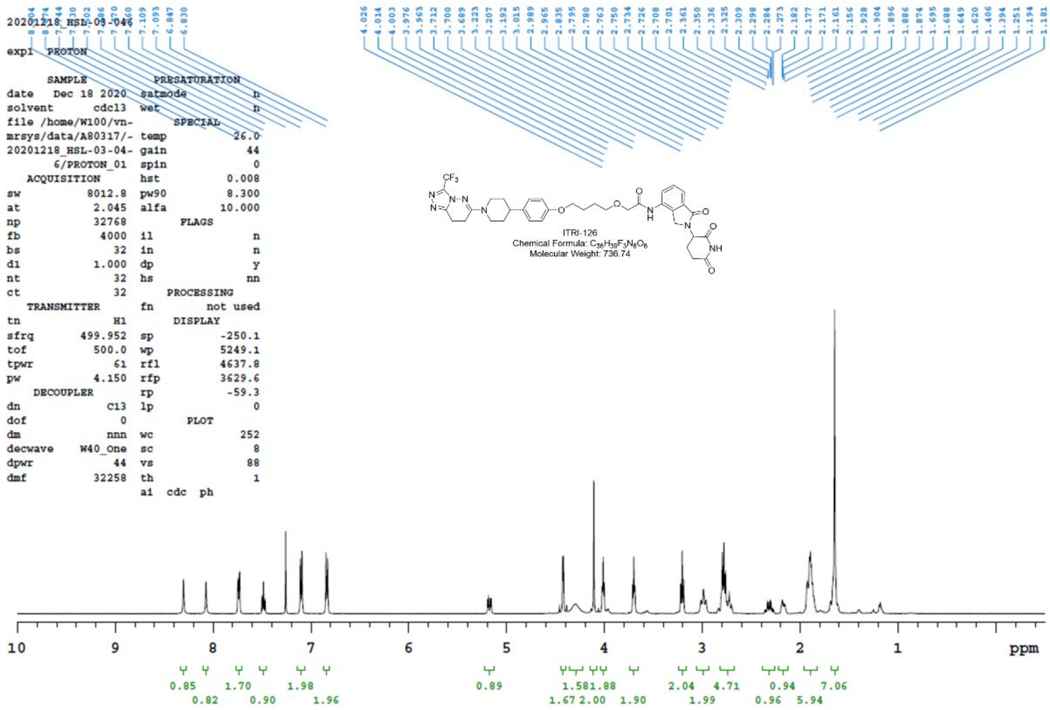

Figure S2. ^{1}H-NMR spectra of ITRI-90 (a), ITRI-125 (b) and ITRI-126 (c).

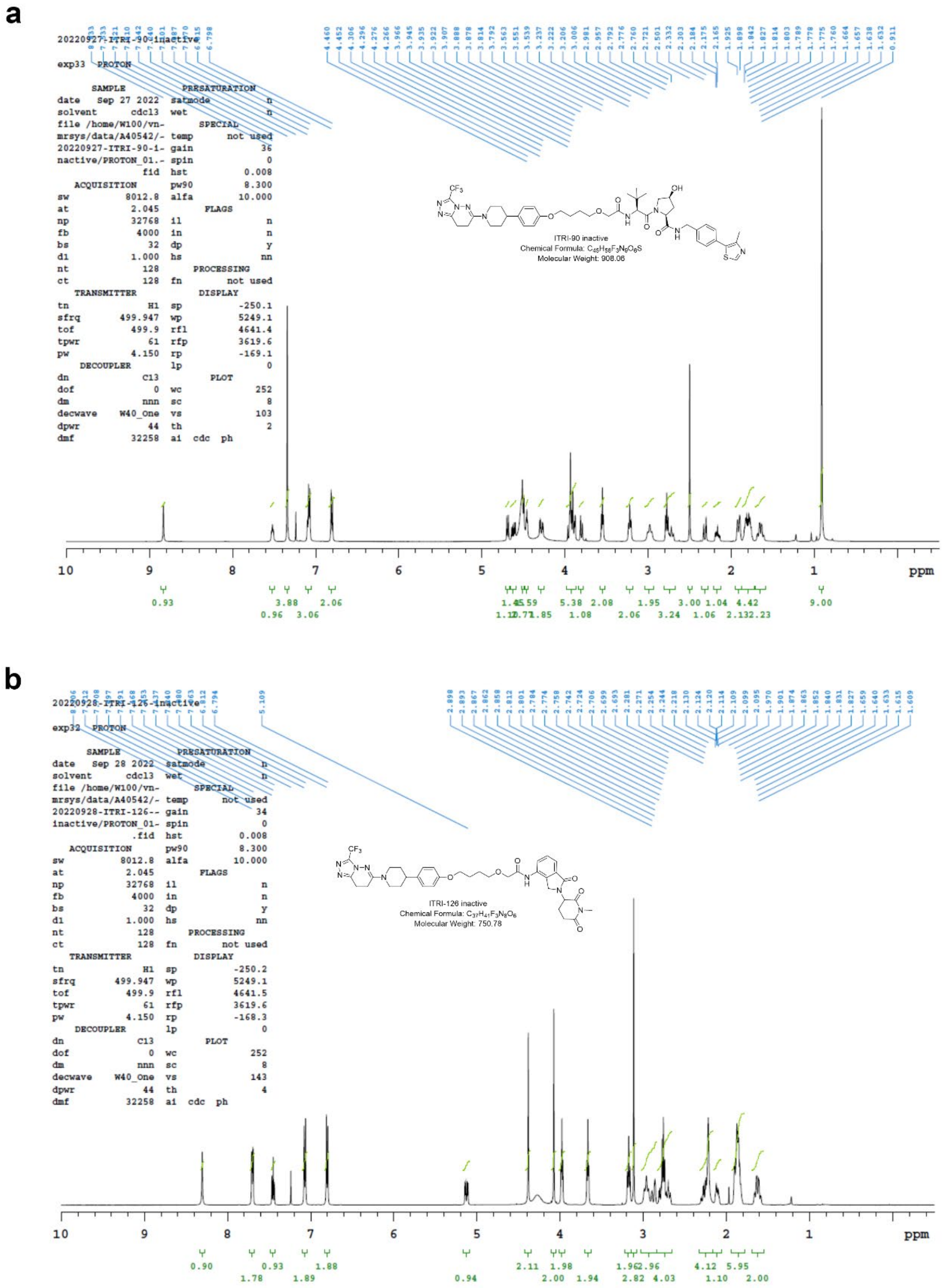

Figure S3. ^{1}H-NMR spectra of inactive ITRI-90 (a) and inactive ITRI-126 (b).

a

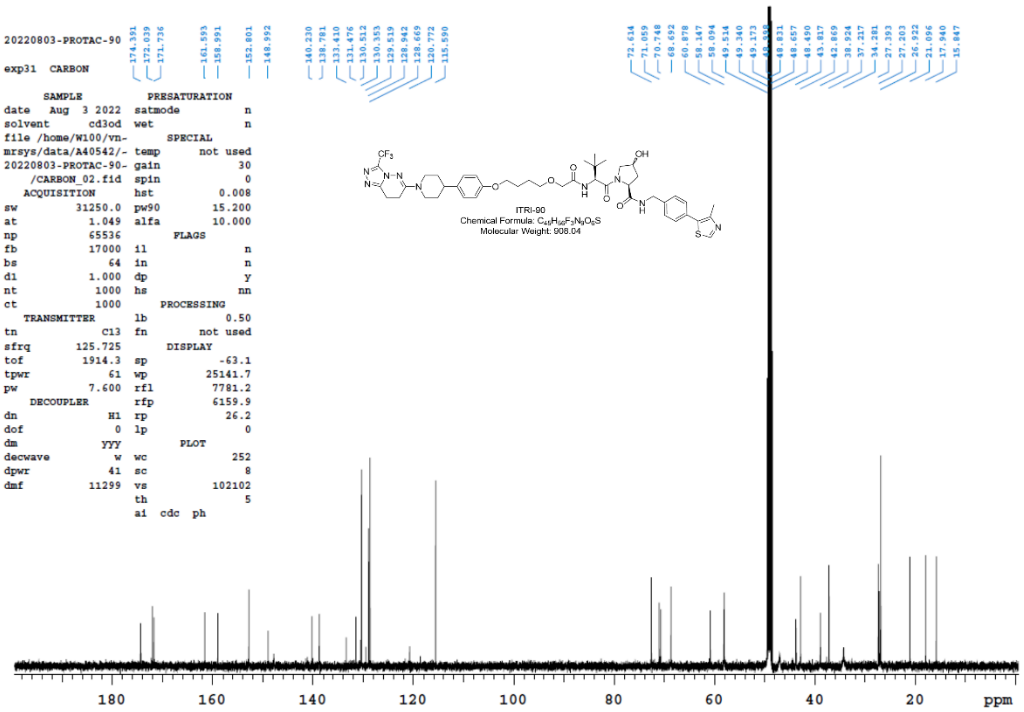

20220803-PROTAC-90
exp31 CARBON
174.391 172.039 171.736 161.593 158.991 152.801 148.992 140.230 138.781 133.410 131.476 130.512 130.353 129.519 128.942 128.669 120.772 115.590
72.614 71.059 70.748 68.692 60.878 58.147 58.094 49.514 49.340 49.173 48.831 48.657 48.490 43.817 42.869 38.924 37.217 34.281 27.393 27.203 26.922 21.096 17.940 15.847
SAMPLE
date Aug 3 2022
solvent cd3od
file /home/W100/vn-mrsys/data/A40542/-20220803-PROTAC-90-/CARBON_02.fid
ACQUISITION
sw 31250.0
at 1.049
np 65536
fb 17000
bs 64
d1 1.000
nt 1000
ct 1000
TRANSMITTER
tn C13
sfrq 125.725
tof 1914.3
tpwr 61
pw 7.600
DECOUPLER
dn H1
dof 0
dm yyy
decwave w
dpwr 41
dmf 11299
PRESATURATION
satmode n
wet n
SPECIAL
temp not used
gain 30
spin 0
hst 0.008
pw90 15.200
alfa 10.000
FLAGS
il n
in n
dp y
hs nn
PROCESSING
lb 0.50
fn not used
DISPLAY
sp -63.1
wp 25141.7
rfl 7781.2
rfp 6159.9
rp 26.2
lp 0
PLOT
wc 252
sc 8
vs 102102
th 5
ai cdc ph
CF3
OH
ITRI-90
Chemical Formula: C45H56F3N9O5S
Molecular Weight: 908.04
180 160 140 120 100 80 60 40 20 ppm

b

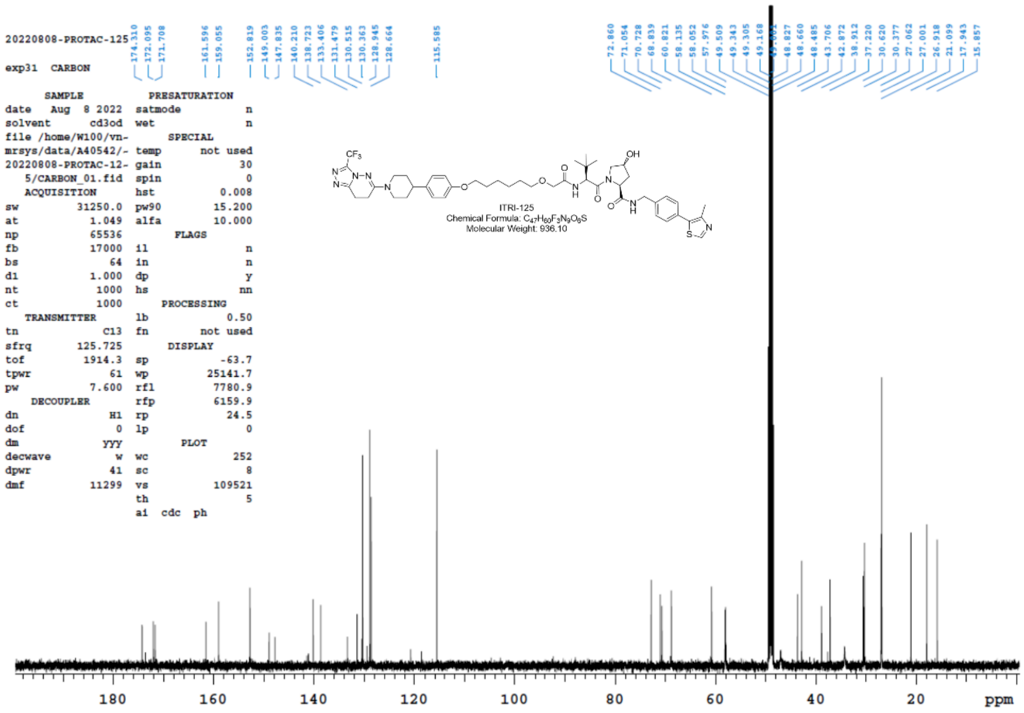

20220808-PROTAC-125
exp31 CARBON
174.310 172.095 171.708 161.596 159.055 152.819 149.003 147.835 140.210 138.723 133.406 131.479 130.515 130.363 128.945 128.664 115.585
72.860 71.054 70.728 68.839 60.821 58.135 58.052 57.976 49.509 49.343 49.305 49.168 48.827 48.660 48.485 43.706 42.872 38.912 37.220 30.620 30.377 27.062 27.001 26.918 21.099 17.943 15.857
SAMPLE
date Aug 8 2022
solvent cd3od
file /home/W100/vn-mrsys/data/A40542/-20220808-PROTAC-12-5/CARBON_01.fid
ACQUISITION
sw 31250.0
at 1.049
np 65536
fb 17000
bs 64
d1 1.000
nt 1000
ct 1000
TRANSMITTER
tn C13
sfrq 125.725
tof 1914.3
tpwr 61
pw 7.600
DECOUPLER
dn H1
dof 0
dm yyy
decwave w
dpwr 41
dmf 11299
PRESATURATION
satmode n
wet n
SPECIAL
temp not used
gain 30
spin 0
hst 0.008
pw90 15.200
alfa 10.000
FLAGS
il n
in n
dp y
hs nn
PROCESSING
lb 0.50
fn not used
DISPLAY
sp -63.7
wp 25141.7
rfl 7780.9
rfp 6159.9
rp 24.5
lp 0
PLOT
wc 252
sc 8
vs 109521
th 5
ai cdc ph
CF3
OH
ITRI-125
Chemical Formula: C47H60F3N9O5S
Molecular Weight: 936.10
180 160 140 120 100 80 60 40 20 ppm

c

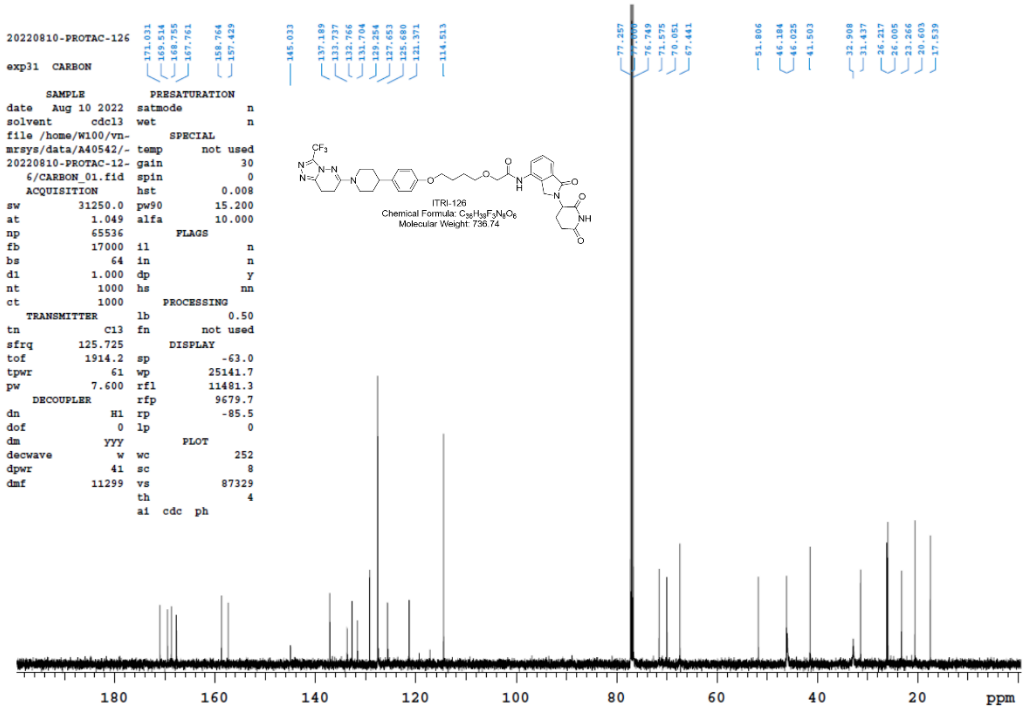

Figure S4. ^{13}C-NMR spectra of ITRI-90 (a), ITRI-125 (b) and ITRI-126 (c).

a

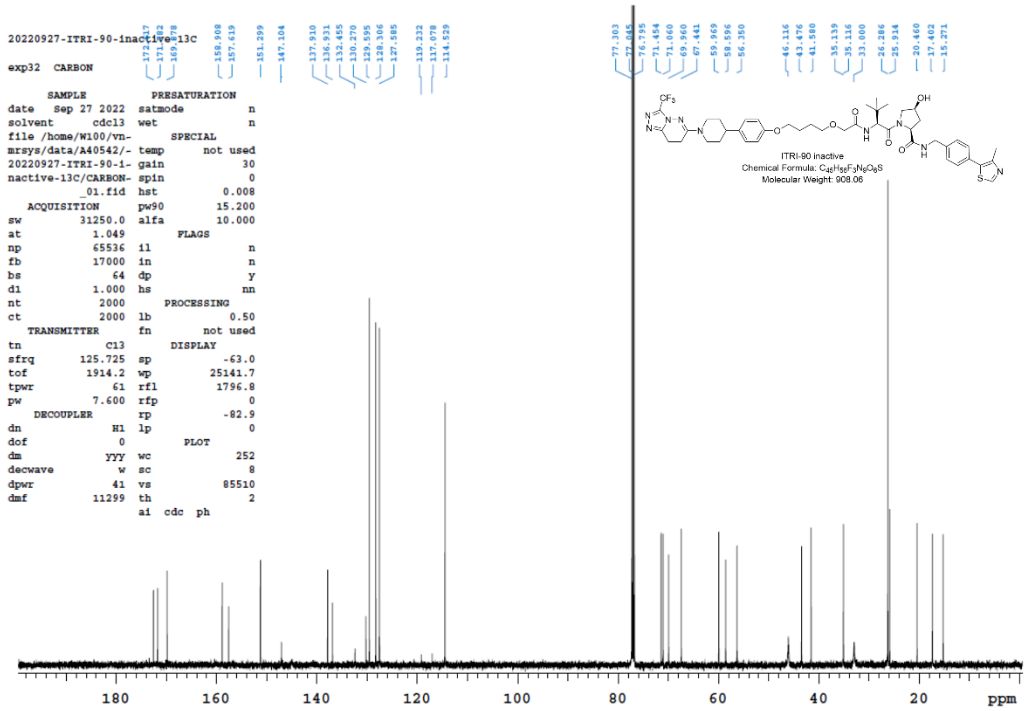

b

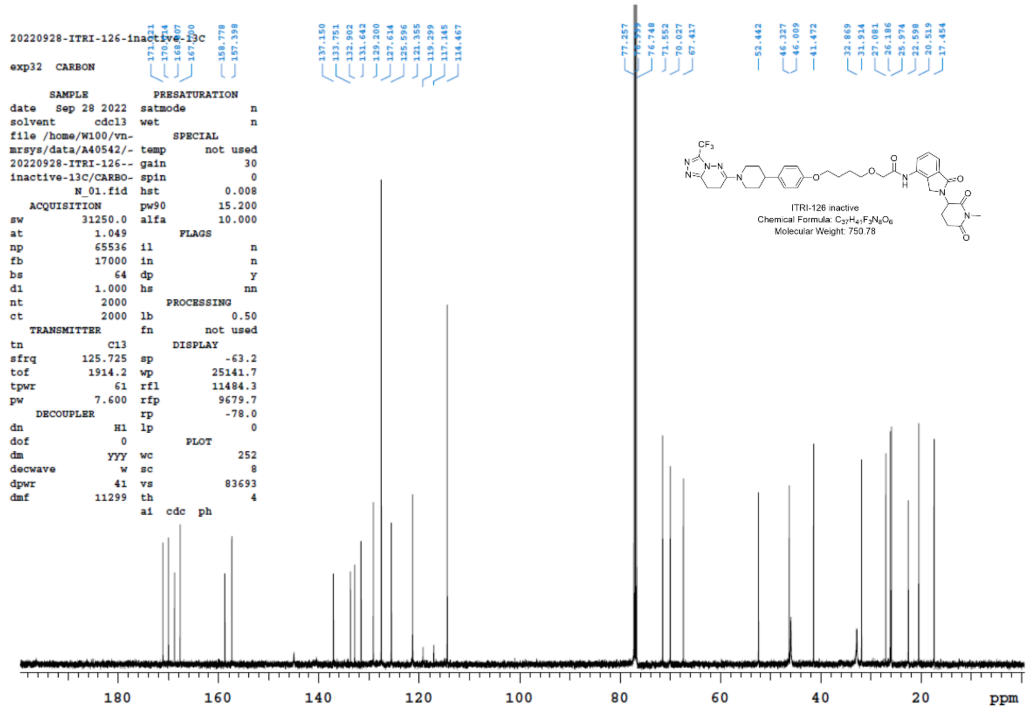

Figure S5. ^{13}C-NMR spectra of inactive ITRI-90 (a) and inactive ITRI-126 (b).

a

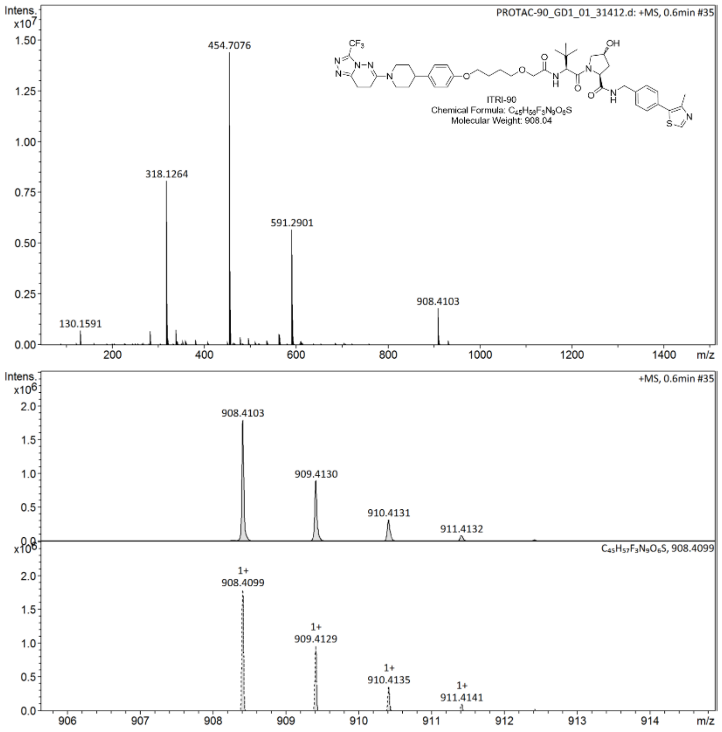
Intens.
x10^7
PROTAC-90_GD1_01_31412.d: +MS, 0.6min #35
454.7076
318.1264
591.2901
908.4103
130.1591
ITRI-90
Chemical Formula: C45H56F3N9O6S
Molecular Weight: 908.04
m/z
+MS, 0.6min #35
908.4103
909.4130
910.4131
911.4132
C45H57F3N9O6S, 908.4099
1+
908.4099
1+
909.4129
1+
910.4135
1+
911.4141
m/z

b

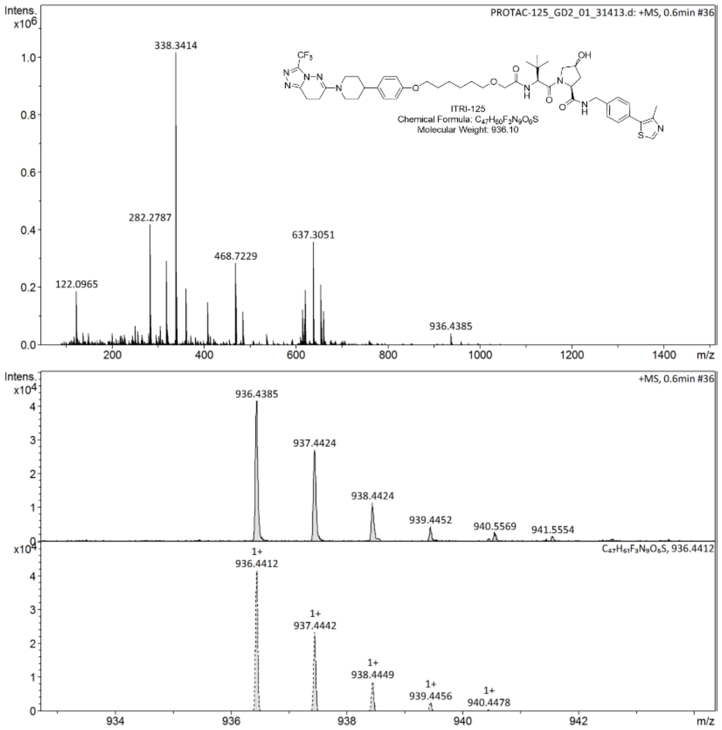
Intens.
x10^6
PROTAC-125_GD2_01_31413.d: +MS, 0.6min #36
338.3414
282.2787
468.7229
637.3051
122.0965
936.4385
ITRI-125
Chemical Formula: C47H60F3N9O6S
Molecular Weight: 936.10
m/z
+MS, 0.6min #36
936.4385
937.4424
938.4424
939.4452
940.5569
941.5554
C47H61F3N9O6S, 936.4412
1+
936.4412
1+
937.4442
1+
938.4449
1+
939.4456
1+
940.4478
m/z

c

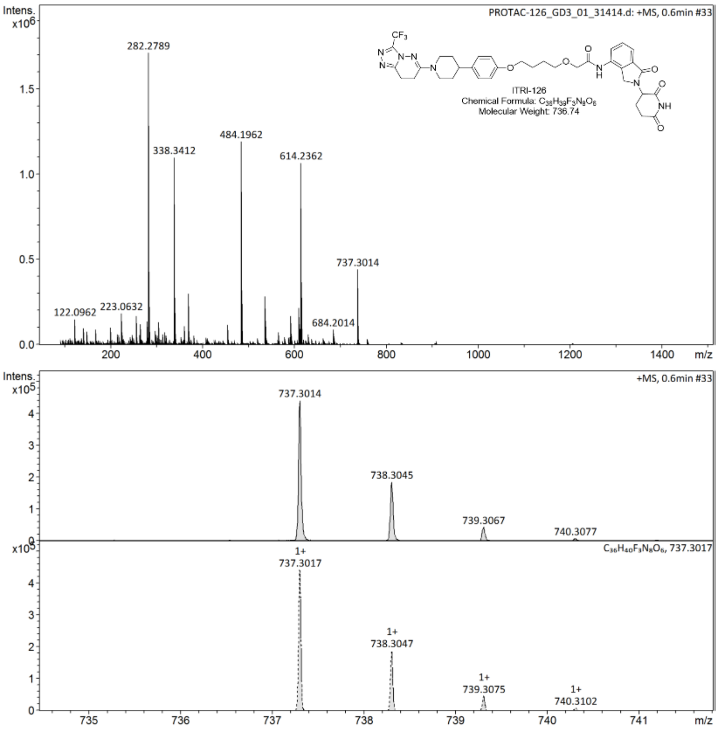

Figure S6. HRMS spectra of ITRI-90 (a), ITRI-125 (b) and ITRI-126 (c).

a

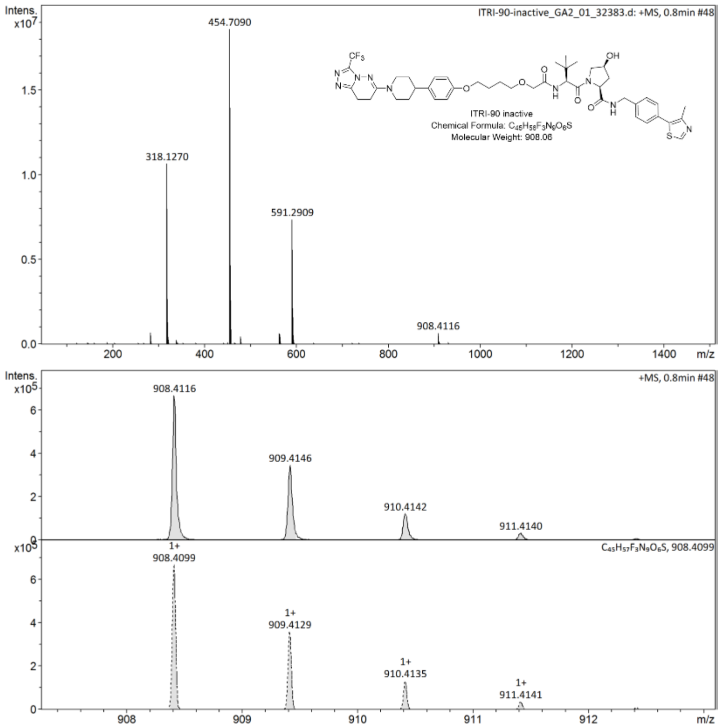

b

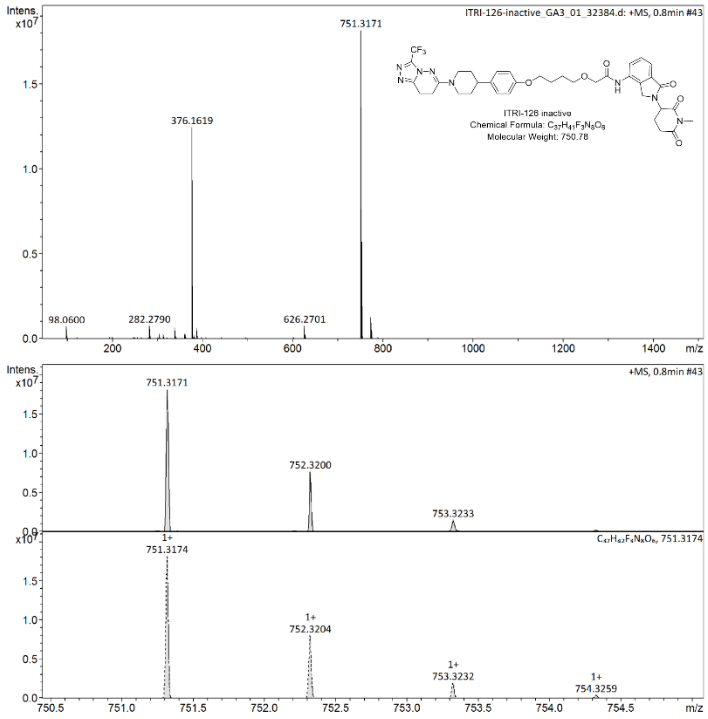

Figure S7. HRMS spectra of inactive ITRI-90 (a) and inactive ITRI-126 (b).

a

Column condition:

Column: ODS-3, Inertsil, 4.6*250 mm, 5 um

Mobile phase: 80 % ACN + 20 % H_2O

Flow: 1.0 mL/min

Column Temp: room temperature

Wavelength: 254 nm

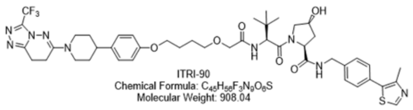

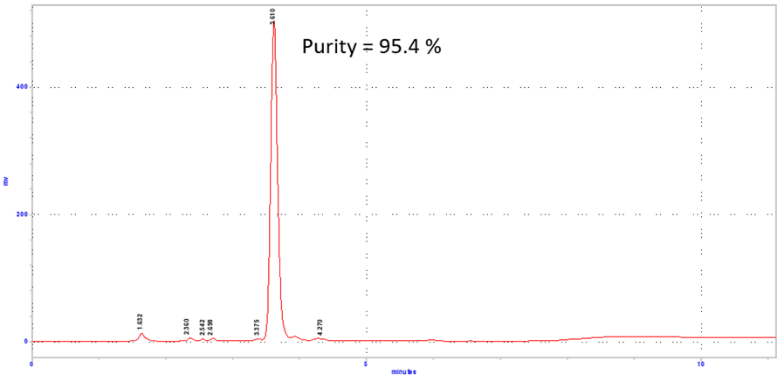

#	Retention time	Area (uv*sec)	Area (%)	height	Height (%)	concentration		theoretical plate number	Symmetry factor	color
1	1.632	94764	2.4919	12.6208	2.3973	0.0000	0.0000	1949	1.115	
2	2.360	22279	0.5858	3.7276	0.7080	0.0000	0.0000	3984	0.687	
3	2.542	7179	0.1888	2.2215	0.4220	0.0000	0.0000	13239	1.129	
4	2.698	14081	0.3703	2.9529	0.5609	0.0000	0.0000	7364	1.248	
5	3.375	7259	0.1909	1.4018	0.2663	0.0000	0.0000	8532	0.509	
6	3.610	3627954	95.3992	500.7910	95.1247	0.0000	0.0000	0	0.000	
7	4.270	29403	0.7732	2.7416	0.5208	0.0000	0.0000	0	0.000	
	Total	3802919		526.457		0.0000	0.0000			

b

Column condition:

Column: ODS-3, Inertsil, 4.6*250 mm, 5 um

Mobile phase: 80 % ACN + 20 % H_2O

Flow: 1.0 mL/min

Column Temp: room temperature

Wavelength: 254 nm

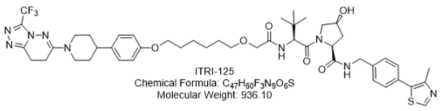

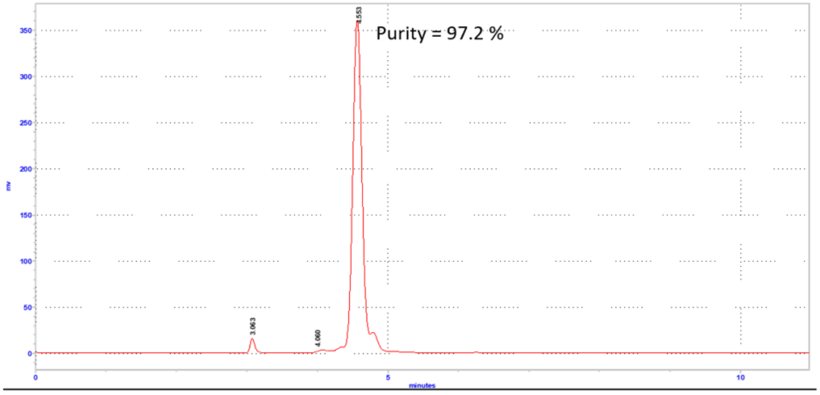

#	Retention time	Area (uv*sec)	Area (%)	height	Height (%)	concentration		theoretical plate number	Symmetry factor	color
1	3.063	70110	2.3590	15.3472	4.2110	0.0000	0.0000	11241	1.497	
2	4.060	14067	0.4733	1.7373	0.4767	0.0000	0.0000	5488	0.859	
3	4.553	2887839	97.1677	347.3708	95.3123	0.0000	0.0000	6692	1.041	
	Total	2972016		364.455		0.0000	0.0000			

c

Column condition:

Column: ODS-3, Inertsil, 4.6*250 mm, 5 um

Mobile phase: 60 % ACN + 40 % H_2O

Flow: 1.0 mL/min

Column Temp: room temperature

Wavelength: 254 nm

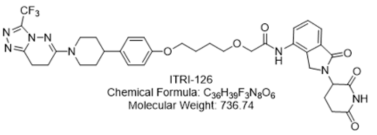

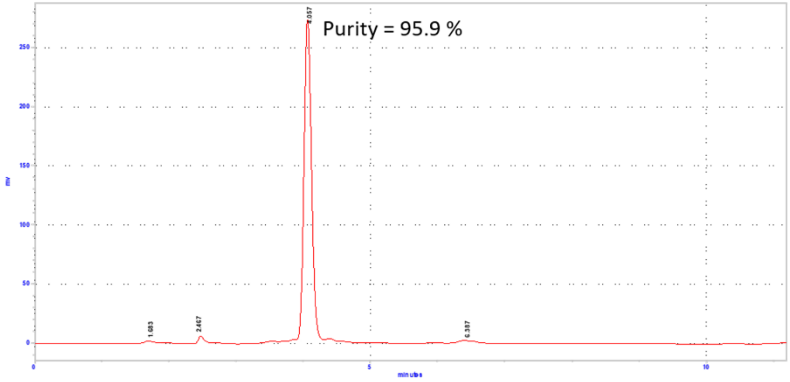

#	Retention time	Area (uv*sec)	Area (%)	height	Height (%)	concentration		theoretical plate number	Symmetry factor	color
1	1.683	18752	0.8741	1.7111	0.6084	0.0000	0.0000	621	1.480	
2	2.467	32767	1.5274	6.0543	2.1526	0.0000	0.0000	5268	1.638	
3	4.057	2056741	95.8759	271.4727	96.5190	0.0000	0.0000	6661	1.203	
4	6.387	36951	1.7225	2.0255	0.7201	0.0000	0.0000	2478	2.140	
	Total	2145211		281.264		0.0000	0.0000			

Figure S8. HPLC spectra of ITRI-90 (a), ITRI-125 (b) and ITRI-126 (c).

a

Column condition:

Column: ODS-3, Inertsil, 4.6*250 mm, 5 um

Mobile phase: 60 % ACN + 40 % H_2O + 0.1 % TFA

Flow: 1.0 mL/min

Column Temp: room temperature

Wavelength: 210 nm

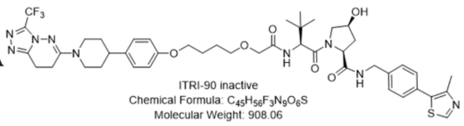

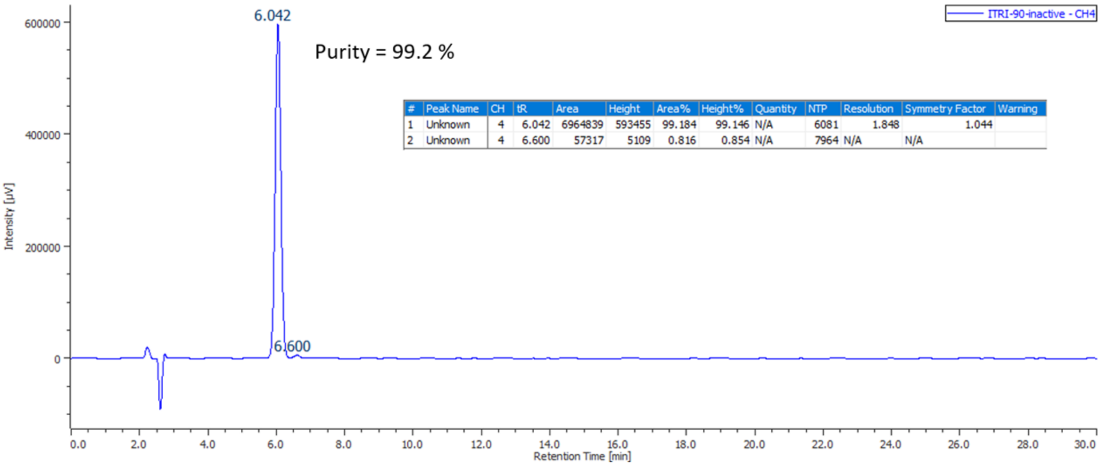

#	Peak Name	CH	tR	Area	Height	Area%	Height%	Quantity	NTP	Resolution	Symmetry Factor	Warning
1	Unknown	4	6.042	6964839	593455	99.184	99.146	N/A	6081	1.848	1.044	
2	Unknown	4	6.600	57317	5109	0.816	0.854	N/A	7964	N/A	N/A	

b

Column condition:

Column: ODS-3, Inertsil, 4.6*250 mm, 5 um

Mobile phase: 60 % ACN + 40 % H_2O + 0.1 % TFA

Flow: 1.0 mL/min

Column Temp: room temperature

Wavelength: 210 nm

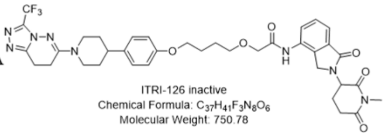

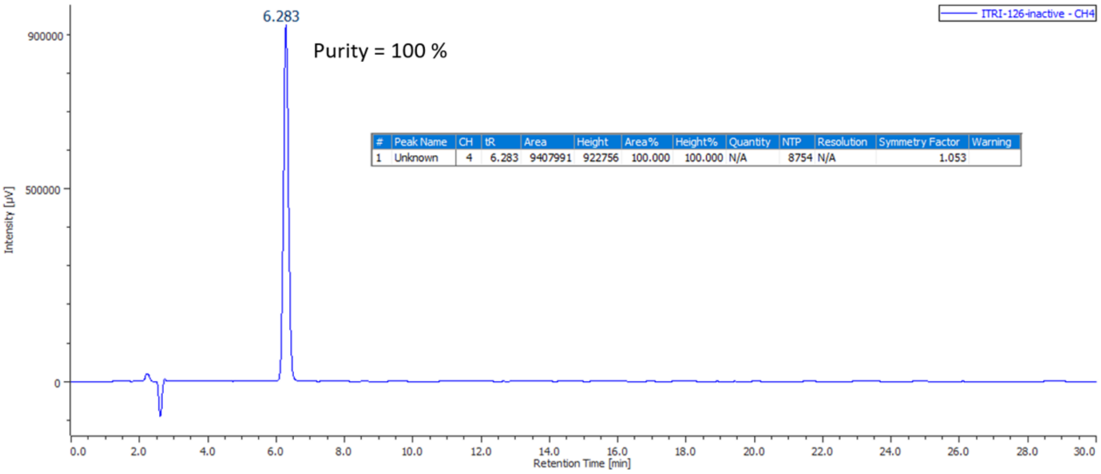

#	Peak Name	CH	tR	Area	Height	Area%	Height%	Quantity	NTP	Resolution	Symmetry Factor	Warning
1	Unknown	4	6.283	9407991	922756	100.000	100.000	N/A	8754	N/A	1.053	

Figure S9. HPLC spectra of inactive ITRI-90 (a) and inactive ITRI-126 (b).

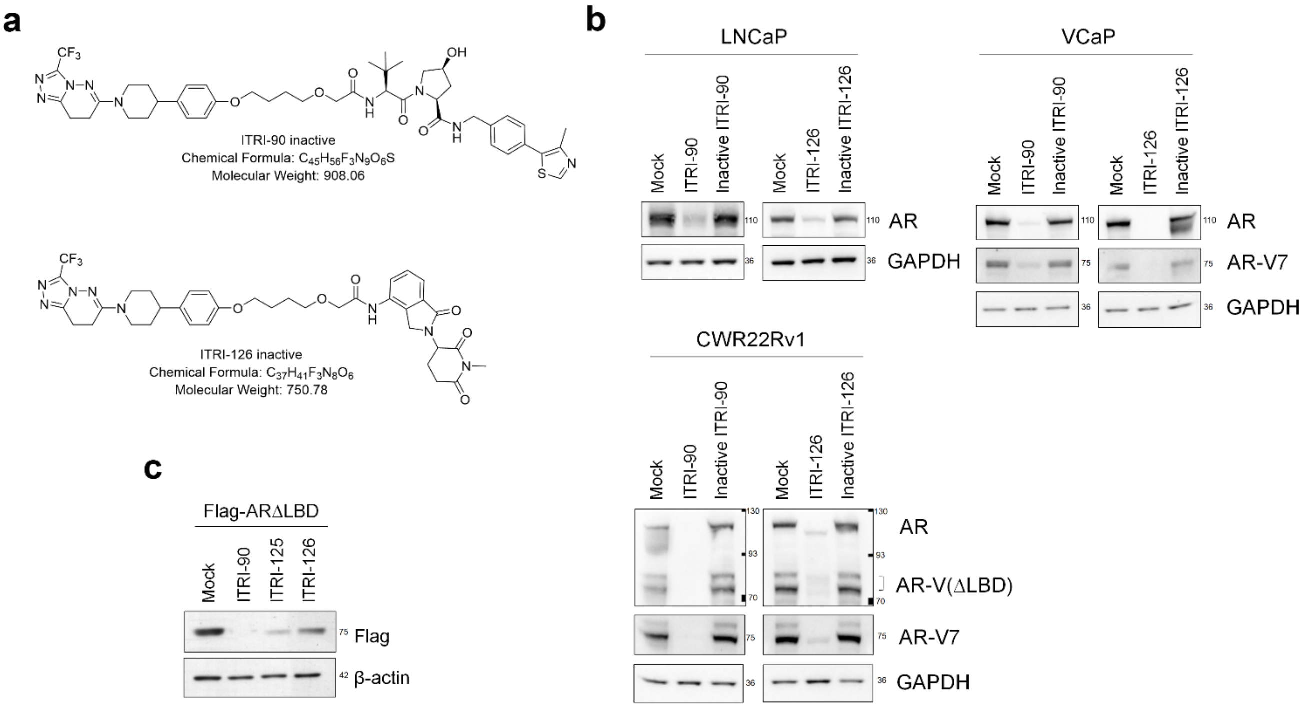

Figure S10. Verification of PROTAC-induced AR and AR-V7 protein degradation. (a) Chemical structures of inactive ITRI-90 and inactive ITRI-126. (b) Representative western blots of AR protein levels in LNCaP, CWR22Rv1 and VCaP cells treated with 24 hours of the AR degraders and inactive degraders. AR was detected by N-terminal antibody which also detects C-terminal truncated AR-V(ΔLBD) in CWR22Rv1 cells. AR-V7 was detected by AR-V7 specific antibody. (c) Degradation of ectopically expressed ARΔLBD in DU145 cells treated with ITRI PROTACs. Cells were transfected with Flag-ARΔLBD one day prior to ITRI compound treatment, following by western analysis of the protein expression after the 24-hour drug treatment.

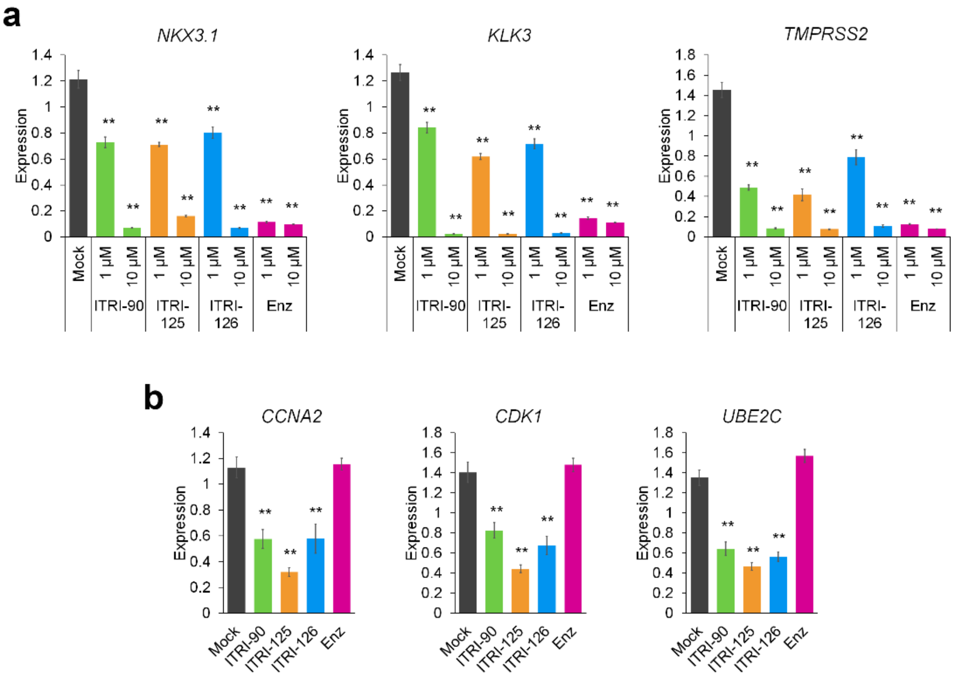

Figure S11. Comparison of AR target inhibition by ITRI-PROTAC and enzalutamide. (a) qRT-PCR analysis of AR target gene expression in LNCaP cells with 24-hour treatment of the indicated compounds. (b) qRT-PCR analysis of AR-V7 target gene expression in CWR22Rv1 cells with 24-hour treatment of 5 µM ITRI-PROTAC or 5 µM enzalutamide (Enz). GAPDH and RPL13A were used as reference genes. Asterisks indicate statistically significant difference between the drug treated groups and mock (** $p < 0.01$, two-tailed Student's t test).

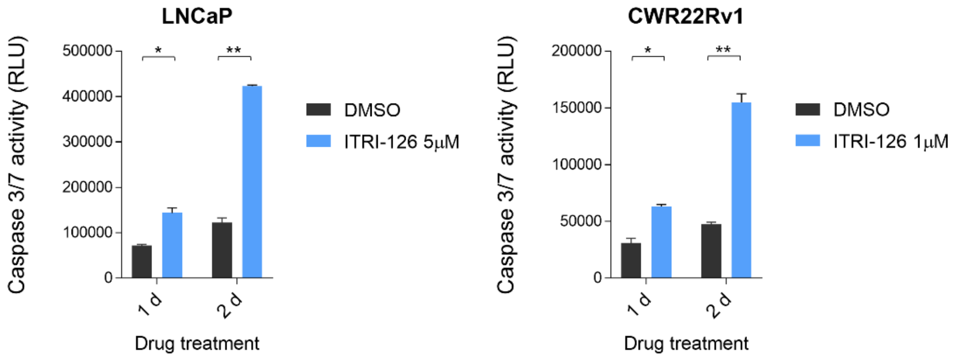

Figure S12. Caspase 3/7 activity of LNCaP and CWR22Rv1 cells upon ITRI-126 treatment. LNCaP and CWR22Rv1 cells were treated with 5 μM and 1 μM of ITRI-126 respectively. After 1 or 2 days of drug treatment, the cells were subjected to caspase 3/7 activity detection using a luminescence-based assay. Caspase activity is indicated by relative light unit (RLU) (* $p < 0.001$, ** $p < 0.0001$, two-tailed Student's t test).

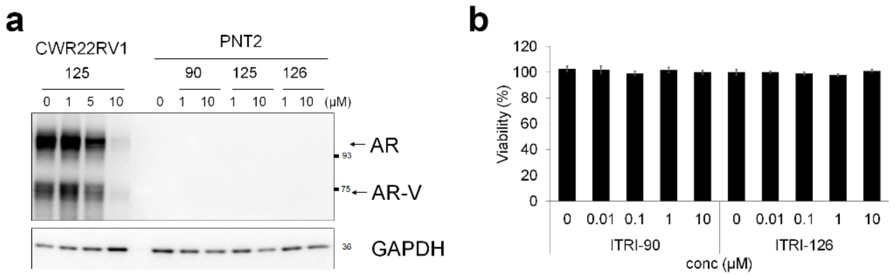

Figure S13. Viability of normal prostate epithelial PNT2 cells treated with ITRI-PROTAC compounds. (a) Western blot of AR in CWR22Rv1 and PNT2 cells with 24 hours of the PROTAC treatment. (b) PNT2 cell viability treated with various dose of ITRI-90 and ITRI-126 for 7 days was detected by Alamar blue. Viability was calculated against the value of mock treatment (0 μM).

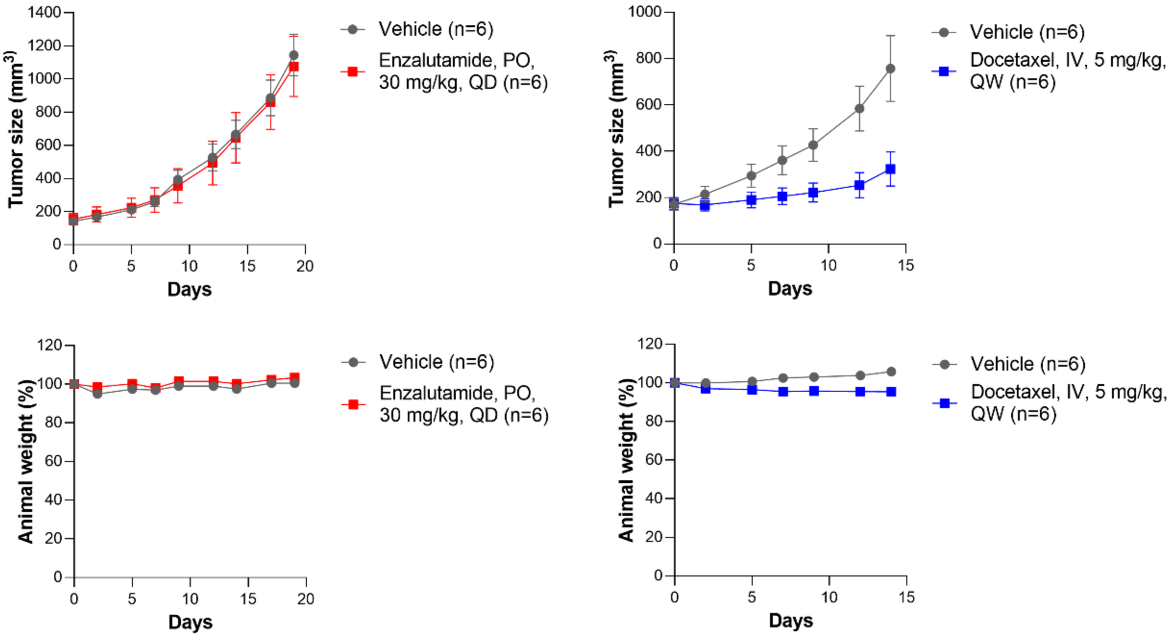

Figure S14. Tumor growth inhibition upon enzalutamide or docetaxel treatment in CWR22Rv1 xenograft model. CWR22Rv1 tumor xenografts implanted in SCID mice were treated with enzalutamide orally at 30 mg/kg once daily dosing, or with docetaxel at 5 mg/kg dosing via intravenous injection once weekly. The scatter plots illustrate tumor size and corresponding animal weight along the course of analyses.

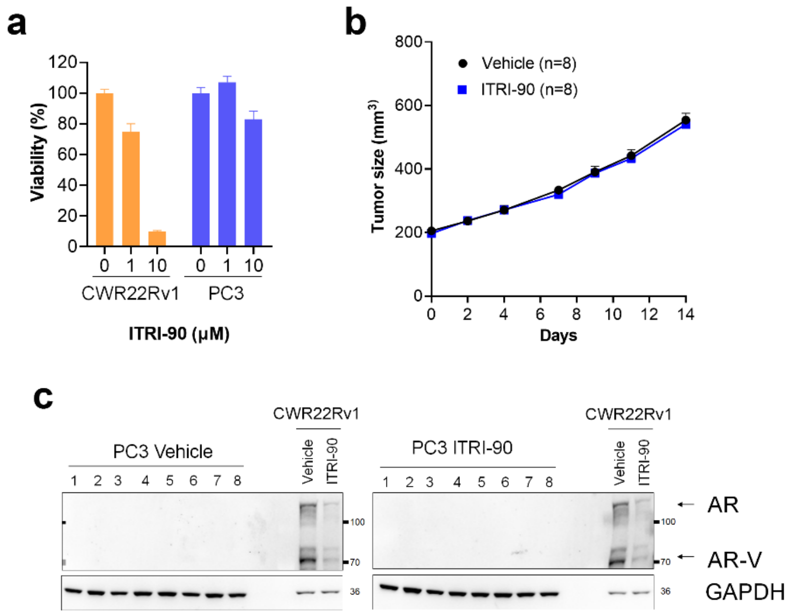

Figure S15. ITRI-90 is ineffective for AR-negative PC3 cells. (a) CWR22Rv1 and PC3 cell viability was detected upon 7 days treatment of ITRI-90. (b,c) Antitumor activity of ITRI-90 towards PC3 tumor xenografts. PC3 tumor xenografts implanted in SCID mice were treated with ITRI-90 orally using 100 mg/kg twice daily dosing. The numbers of tumor in each group is as indicated. (b) Scatter plots of the tumor size. (c) Western blotting of AR and AR-V proteins in the tumors collected at the final time points. CWR22Rv1 xenograft samples from Figure 7i were used as positive control for AR and AR-V expression.

Table S1. Relative affinity binding, *in vitro* potencies and *in vitro* stability of candidate compounds.

Binder category	Compound	ASMS relative binding (%)	AR degradation	Cell viability (%)	Plasma stability $T_{1/2}$ (hr)	MLM stability $T_{1/2}$ (min)
ARN binder	ARN-5<br>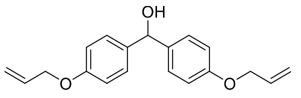	52.2	NA	NA	NA	NA
	ARN-5-derived ITRI-49	32.5	22.20%	51.5 ± 1.6	> 4	< 5
	ARN-5-derived ITRI-52	18.9	35.25%	50.55 ± 0.48	> 4	< 5
NCS binder	NCS-4<br>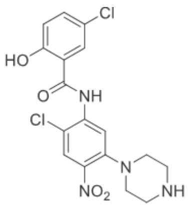	61.9	NA	NA	NA	NA
	NSC-4-derived ITRI-86	51.8	26.45%	82.8 ± 1.11	> 4	> 30
	NSC-4-derived ITRI-87	50.2	26.95%	76.47 ± 1.16	> 4	> 30
AZD binder	AZD-1<br>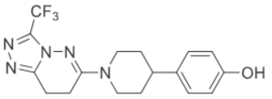	29.4	NA	NA	NA	NA
	AZD-1 derived ITRI-90	15.9	98.28%	10.53 ± 2.42	> 4	> 30
	AZD-1 derived ITRI-125	13.6	92.53%	18.54 ± 7.67	> 4	> 30

Table S2. Primers used in qRT-PCR analysis

Primer name	Sequence
CCNA2-F	CAGAAAACCATTGGTCCCTC
CCNA2-R	CACTCACTGGCTTTTCATCTTC
CDC20-F	CCTCTGGTCTCCCCATTAC
CDC20-R	ATGTGTGACCTTTGAGTTCAG
CDK1-F	CCTAGTACTGCAATTCGGGAAATT
CDK1-R	CCTGGAATCCTGCATAAGCAC
EDN2-F	CGTCCTCATCTCATGCCC
EDN2-R	AGGCCGTAAGGAGCTGTCT
GAPDH-F	AATCCCATCACCATCTTCCAG
GAPDH-R	CCTTCTCCATGGTGGTGAAGAC
NKX3.1-F	CCAGCACAAAAGGCAGGGTAG
NKX3.1-R	TGGGGAAAGCAAGATGGATTC
PSA-F	ACCAGAGGAGTTCTTGACCCCAAA
PSA-R	CCCCAGAATCACCCGAGCAG
RPL13A-F	GCCATCGTGGCTAAACAGGTA
RPL13A-R	GTTGGTGTTCATCCGCTTGC
TMPRSS2-F	GAAAGGGAAGACCTCAGAAGTGC
TMPRSS2-R	CGATAAATCCAGTCCGTGAATACC
UBE2C-F	GACCTGAGGTATAAGCTCTCGC
UBE2C-R	TTACCCTGGGTGTCCACG
UGT2B17-F	ACCAGCCAAACCCTTGC
UGT2B17-R	GGCTGATGCAATCATGTTG
