# Supplementary material for: Targeting androgen receptor and the variants by an orally bioavailable Proteolysis Targeting Chimeras compound in castration resistant prostate cancer
Source: eBioMedicine. 2023 Mar 7;90:104500. doi: 10.1016/j.ebiom.2023.104500 (PMC10011747; doi:10.1016/j.ebiom.2023.104500)
Supplement: Caption for Supplementary Material does not contain scientific information for the reseach article itself, and is considered to be dispensable. [file mmc3.docx]

Caption for supplementary material

Supplementary data

File name: Manuscript EBIOM-D-22-02531_ Supplementary data
