# Supplementary material for: Targeting androgen receptor and the variants by an orally bioavailable Proteolysis Targeting Chimeras compound in castration resistant prostate cancer
Source: eBioMedicine. 2023 Mar 7;90:104500. doi: 10.1016/j.ebiom.2023.104500 (PMC10011747; doi:10.1016/j.ebiom.2023.104500)
Supplement: Reagent Validation [file mmc4.pdf]

Reagent Validation

Table of content

LNCaP Cell Line DNA Typing Report (Page 2-7)

CWR22Rv1 Cell Line DNA Typing Report (Page 8-12)

VCaP Cell Line DNA Typing Report (Page 13-17)

C4-2B Cell Line DNA Typing Report (Page 18-22)

C4-2B/Enz^{R} Cell Line DNA Typing Report (Page 23-28)

PNT2 Cell Line DNA Typing Report (Page 29-33)

HEK293T Cell Line DNA Typing Report (Page 34-39)

DU145 Cell Line DNA Typing Report (Page 40-44)

PC3 Cell Line DNA Typing Report (Page 45-49)

Anti-AR antibody datasheet (Page 50-51)

Anti-AR-V7 antibody datasheet (Page 52)

Anti-GAPDH antibody datasheet (Page 53-54)

Anti-β-actin antibody datasheet (Page 55-56)

Anti-Flag-M2 antibody datasheet (Page 57-60)

Anti-HA antibody datasheet (Page 61-63)

Recombinant human androgen receptor protein datasheet (Page 64-66)

Cell Line DNA Typing Report

Case Number: CID20220071

Report Date: 08/24/2022

Genelabs Life Science Corp.
12F.-6, No.3, Yuanqu St.,
Nangang Dist.,
Taipei City 115, Taiwan
TEL: +886-2-26557678
FAX: +886-2-26557572
E-mail: cellid@genelabs.com.tw

Sample Information:

i. Applicant Name: 王齡玉 Ling-Yu Wang

ii. Institution: 長庚大學生化科 Department of Biochemistry and Molecular Biology, Chang Gung University, Taoyuan, Taiwan

iii. Sample Description: LNCaP

iv. Sample type: Cell Pellet

v. Sample Received Date: 08/12/2022

Test Description:

DNA of the sample is extracted by Roche MagNA Pure Compact System.

DNA conc. = 111.9 ng/µl; OD260/280 = 2.00 ; OD260/230 = 1.96

The STR loci are amplified by Promega GenePrint® 24 System.

The CE analysis is performed on ABI PRISM 3730 GENETIC ANALYZER.

The raw data is analyzed by GeneMapper® Software V3.7.

The STR analysis is operated and reported by Mission Biotech.

This report is issued by:

Laboratory Director　　　General Manager

STR Analysis Result:

ANSI/ATCC ASN-0002 STR Loci	Repeat Numbers	Extended STR Loci	Repeat Numbers	Extended STR Loci	Repeat Numbers
**D5S818**	11,12	D3S1358	16,16	DYS391*	10
**D13S317**	10,12	D1S1656	16,16	D8S1179	12,14
**D7S820**	9.1,10.3	D2S441	11.3,11.3	D12S391	21,22
**D16S539**	11,11	D10S1248	14,16	D19S433	13.2,15
**vWA**	16,18	Penta E	12,16	FGA	19,20
**TH01**	9,9	D18S51	11,12	D22S1045	15,17
**Amelogenin**	X,Y	D2S1338	16,16	Case Number: CID20220071<br>Test Date: 08/18/2022	
**TPOX**	8,9	Penta D	12,12		
**CSF1PO**	10,11	D21S11	29,31.2,32.2		

Allele Report

Case Number: CID20220071

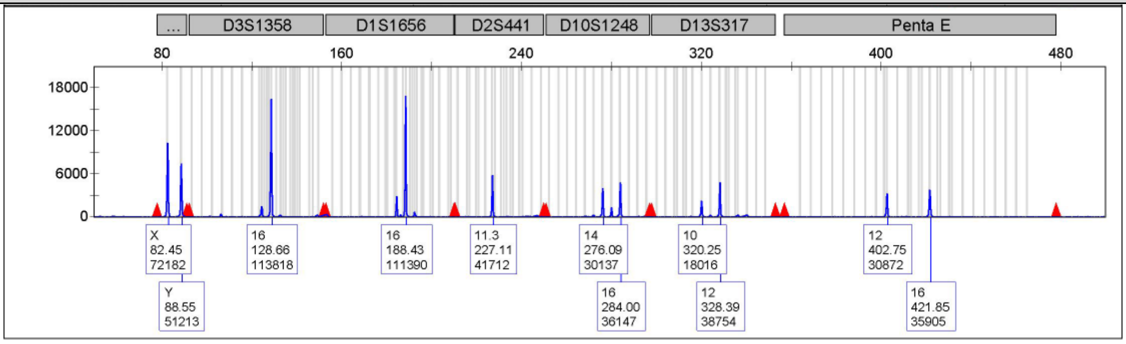

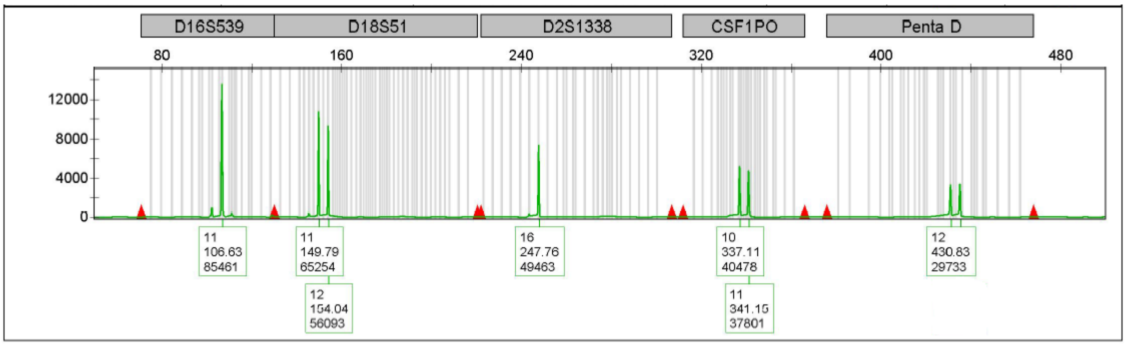

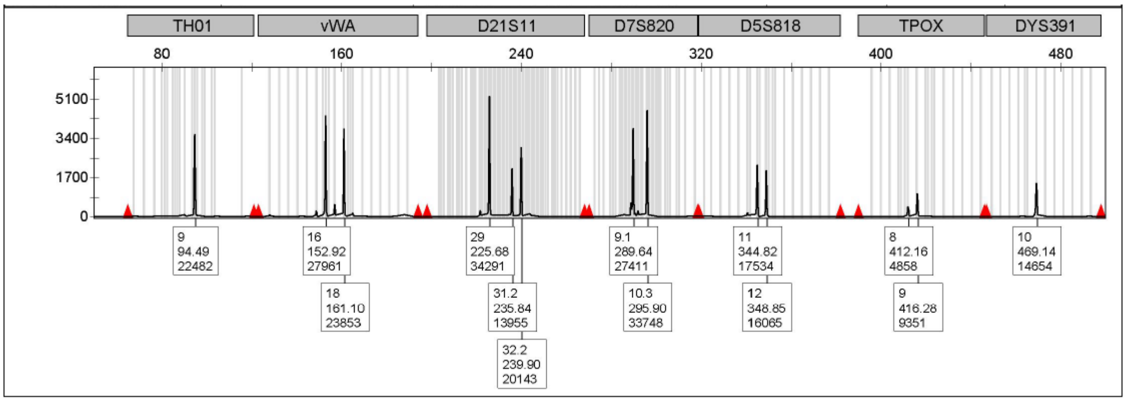

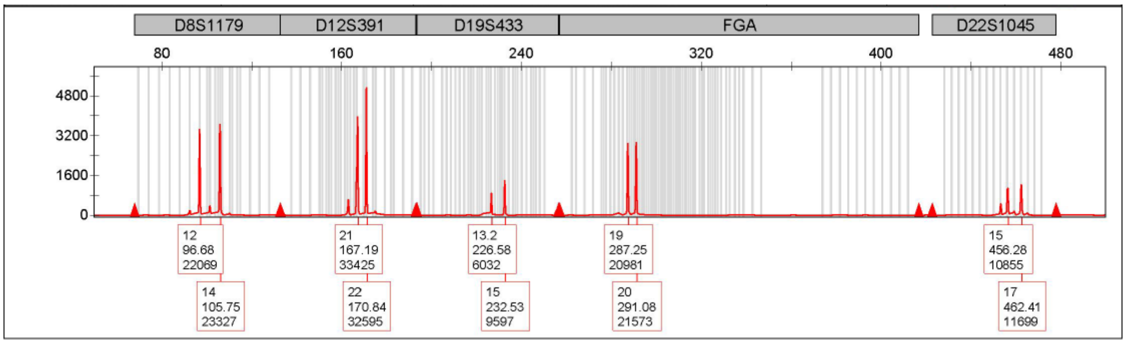

	Dye	Sample File Name	Marker	Allele	Size	Height	Area
1	B,1	039_B10_LNCaP.fsa	AMEL	X	82.45	10338	72182
2	B,2	039_B10_LNCaP.fsa	AMEL	Y	88.55	7482	51213
3	B,3	039_B10_LNCaP.fsa	D3S1358	16	128.66	16452	113818
4	B,4	039_B10_LNCaP.fsa	D1S1656	16	188.43	16855	111390
5	B,5	039_B10_LNCaP.fsa	D2S441	11.3	227.11	5840	41712
6	B,6	039_B10_LNCaP.fsa	D10S1248	14	276.09	4036	30137
7	B,7	039_B10_LNCaP.fsa	D10S1248	16	284	4782	36147
8	B,8	039_B10_LNCaP.fsa	D13S317	10	320.25	2277	18016
9	B,9	039_B10_LNCaP.fsa	D13S317	12	328.39	4746	38754
10	B,10	039_B10_LNCaP.fsa	Penta E	12	402.75	3324	30872
11	B,11	039_B10_LNCaP.fsa	Penta E	16	421.85	3864	35905
12	G,1	039_B10_LNCaP.fsa	D16S539	11	106.63	13655	85461
13	G,2	039_B10_LNCaP.fsa	D18S51	11	149.79	10854	65254
14	G,3	039_B10_LNCaP.fsa	D18S51	12	154.04	9382	56093
15	G,4	039_B10_LNCaP.fsa	D2S1338	16	247.76	7455	49463
16	G,5	039_B10_LNCaP.fsa	CSF1PO	10	337.11	5215	40478
17	G,6	039_B10_LNCaP.fsa	CSF1PO	11	341.15	4747	37801
18	G,7	039_B10_LNCaP.fsa	Penta D	12	430.83	3378	29733
19	Y,1	039_B10_LNCaP.fsa	TH01	9	94.49	3592	22482
20	Y,2	039_B10_LNCaP.fsa	vWA	16	152.92	4408	27961
21	Y,3	039_B10_LNCaP.fsa	vWA	18	161.1	3828	23853
22	Y,4	039_B10_LNCaP.fsa	D21S11	29	225.68	5214	34291
23	Y,5	039_B10_LNCaP.fsa	D21S11	31.2	235.84	2134	13955
24	Y,6	039_B10_LNCaP.fsa	D21S11	32.2	239.9	3022	20143
25	Y,7	039_B10_LNCaP.fsa	D7S820	9.1	289.64	3867	27411
26	Y,8	039_B10_LNCaP.fsa	D7S820	10.3	295.9	4638	33748
27	Y,9	039_B10_LNCaP.fsa	D5S818	11	344.82	2273	17534
28	Y,10	039_B10_LNCaP.fsa	D5S818	12	348.85	2051	16065
29	Y,11	039_B10_LNCaP.fsa	TPOX	8	412.16	541	4858
30	Y,12	039_B10_LNCaP.fsa	TPOX	9	416.28	1042	9351
31	Y,13	039_B10_LNCaP.fsa	DYS391	10	469.14	1520	14654
32	R,1	039_B10_LNCaP.fsa	D8S1179	12	96.68	3490	22069
33	R,2	039_B10_LNCaP.fsa	D8S1179	14	105.75	3677	23327
34	R,3	039_B10_LNCaP.fsa	D12S391	21	167.19	3982	33425
35	R,4	039_B10_LNCaP.fsa	D12S391	22	170.84	5156	32595
36	R,5	039_B10_LNCaP.fsa	D19S433	13.2	226.58	889	6032

37	R,6	039_B10_LNCaP.fsa	D19S433	15	232.53	1433	9597
38	R,7	039_B10_LNCaP.fsa	FGA	19	287.25	2914	20981
39	R,8	039_B10_LNCaP.fsa	FGA	20	291.08	2951	21573
40	R,9	039_B10_LNCaP.fsa	D22S1045	15	456.28	1150	10855
41	R,10	039_B10_LNCaP.fsa	D22S1045	17	462.41	1250	11699

The comparison between case result and STR profile database:

Cell Name	Locus name / STR Repeat Number									Match (%)	STR Profile Database*
	D5S818	D13S317	D7S820	D16S539	VWA	TH01	AM	TPOX	CSF1PO		
Test Sample	11,12	10,12	9.1,10.3	11,11	16,18	9,9	X,Y	8,9	10,11		
LNCaP	11,12	10,12	9.1,10.3	11,11	16,18	9,9	X,X	8,9	10,11	100	ExPASy
LNCaP clone FGC	11,12	10,12	9.1,10.3	11,11	16,18	9,9	X,Y	8,9	10,11	100	ExPASy
LNCaP C4	11,12	10,11	9,9.1, 10.3	11,11	16,18, 19	9,9	X,Y	8,9	10,11	86.67	ExPASy
LNCaP clone FGC	11,12	10,12	9,11	11,11	16,18	9,9	X,Y	8,9	10,11	85.71	ExPASy
LNCaP C4-2B	11,12	10,11	8.1,9. 1,10.3	10,11	16,18	9,9	X,Y	8,9	9,10,11	83.87	ExPASy
LNCaP	11,12	10,11, 12,13	9,11	11,11	16,18	9,9	X,X	8,9	10,11	80	ExPASy

* Online STR Analysis is performed on some bioresource websites as below:

https://web.expasy.org/cellosaurus-str-search/ (ExPASy)

https://www.atcc.org/STR_Database.aspx (ATCC)

https://www.dsmz.de/services/services-human-and-animal-cell-lines/online-str-analysis.html (DSMZ)

* This table just shows the top 10 cell lines within database those matches are ≥80%, and if there isn't any match ≥80%, it just shows the cell line which scores highest match with the test sample. If necessary, please utilize the online STR analysis on ATCC/DSMZ website for detailed comparison.

The interpretation of comparison:

Depending on the comparison, the highest 100% match is between the test sample and two cell line(LNCaP, LNCaP clone FGC) within database, and there are other four cell lines those matches are ≥80%.

Referring to ASN-0002-2011 (Authentication of Human Cell Lines: Standardization of STR Profiling), Cell line samples matching at ≥80% of alleles across the eight (8) core loci are said to be related, allowing authentication of the sample being tested. STR profiles are said to be unrelated when they match at <55% of alleles. STR profiles matching at 55-80% of alleles may be related and warrant further investigation as there may be overlap with a small number of cell lines that show marked genetic instability and slip below the 80% threshold.

- ***This STR analysis service is for research purposes only, and any medical usage is not allowed.***

REPORT DOWNLOAD

勁因科技有限公司

Genelabs Life science Corporation

www.genelabs.com.tw

Phone:(02)26557678, (04)22633813, (06)2094380

Freecall:0800-231914, 0800-094380

Cell Line DNA Typing Report

Case Number: CID20220072

Report Date: 08/24/2022

Genelabs Life Science Corp.
12F.-6, No.3, Yuanqu St.,
Nangang Dist.,
Taipei City 115, Taiwan
TEL: +886-2-26557678
FAX: +886-2-26557572
E-mail: cellid@genelabs.com.tw

Sample Information:

i. Applicant Name: 王齡玉 Ling-Yu Wang

ii. Institution: 長庚大學生化科 Department of Biochemistry and Molecular Biology, Chang Gung University, Taoyuan, Taiwan

iii. Sample Description: CWR22Rv1

iv. Sample type: Cell Pellet

v. Sample Received Date: 08/12/2022

Test Description:

DNA of the sample is extracted by Roche MagNA Pure Compact System.

DNA conc. = 47.3 ng/μl; OD260/280 = 1.98 ; OD260/230 = 1.79

The STR loci are amplified by Promega GenePrint® 24 System.

The CE analysis is performed on ABI PRISM 3730 GENETIC ANALYZER.

The raw data is analyzed by GeneMapper® Software V3.7.

The STR analysis is operated and reported by Mission Biotech.

This report is issued by:

Liang Kuei Chang — Laboratory Director

James Chung — General Manager

STR Analysis Result:

ANSI/ATCC ASN-0002 STR Loci	Repeat Numbers	Extended STR Loci	Repeat Numbers	Extended STR Loci	Repeat Numbers
**D5S818**	11,13	D3S1358	15,15	DYS391*	11
**D13S317**	9,12	D1S1656	17,18,19	D8S1179	13,14
**D7S820**	9,10,11	D2S441	11,12	D12S391	18,18
**D16S539**	12,12	D10S1248	13,15	D19S433	13,14
**vWA**	15,21	Penta E	5,13	FGA	20,23
**TH01**	6,9.3	D18S51	13,14	D22S1045	15,15
**Amelogenin**	X,Y	D2S1338	17,18	Case Number: CID20220072<br>Test Date: 08/18/2022	
**TPOX**	8,8	Penta D	9,12		
**CSF1PO**	10,11	D21S11	30,30		

Allele Report

Case Number: CID20220072

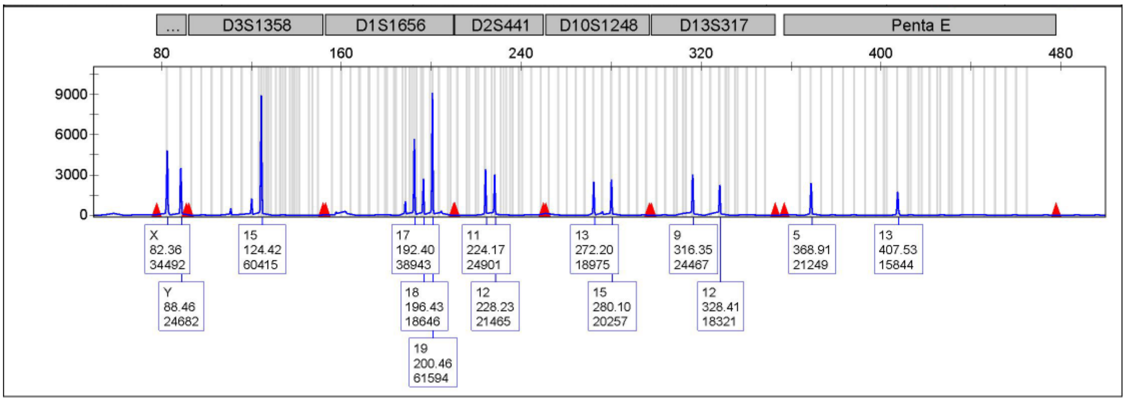

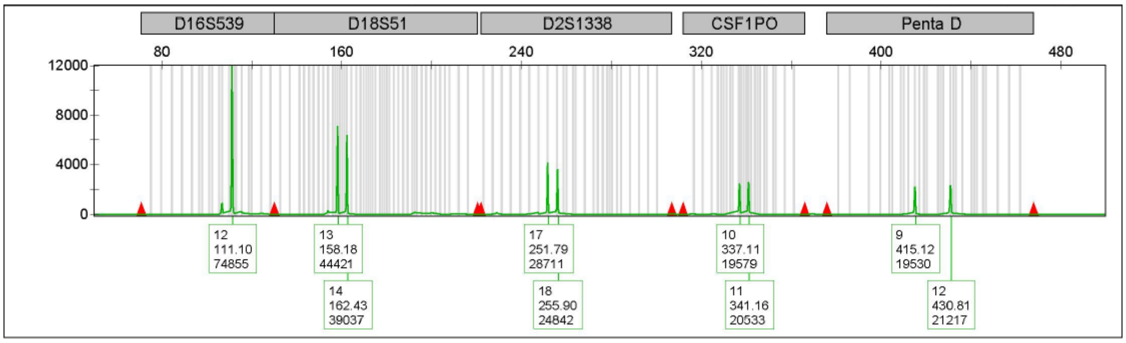

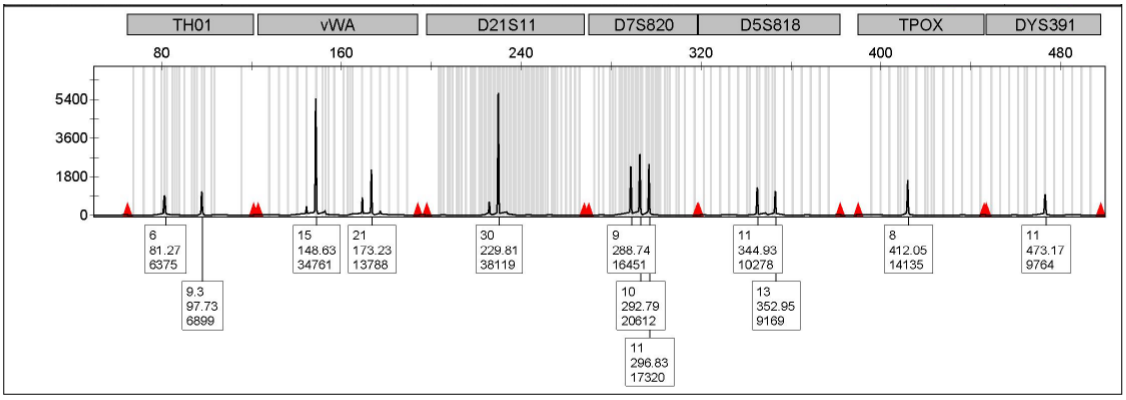

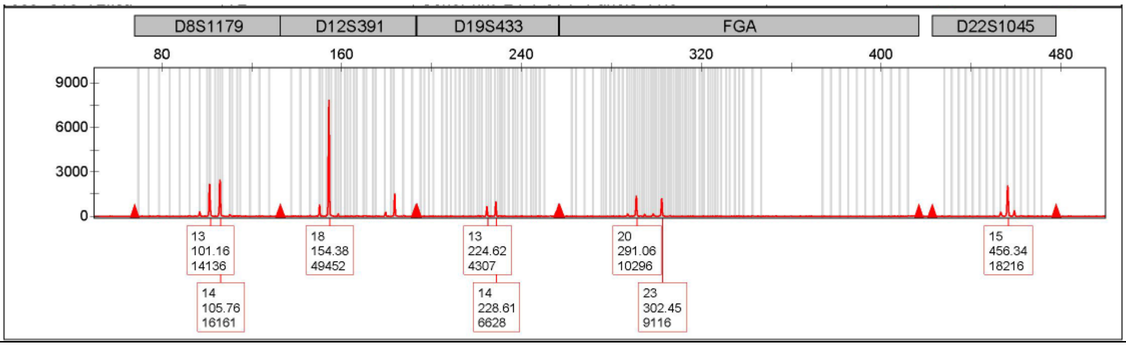

	Dye	Sample File Name	Marker	Allele	Size	Height	Area
1	B,1	038_C10_CWR22Rv1.fsa	AMEL	X	82.36	4809	34492
2	B,2	038_C10_CWR22Rv1.fsa	AMEL	Y	88.46	3555	24682
3	B,3	038_C10_CWR22Rv1.fsa	D3S1358	15	124.42	8918	60415
4	B,4	038_C10_CWR22Rv1.fsa	D1S1656	17	192.4	5701	38943
5	B,5	038_C10_CWR22Rv1.fsa	D1S1656	18	196.43	2735	18646
6	B,6	038_C10_CWR22Rv1.fsa	D1S1656	19	200.46	9154	61594
7	B,7	038_C10_CWR22Rv1.fsa	D2S441	11	224.17	3429	24901
8	B,8	038_C10_CWR22Rv1.fsa	D2S441	12	228.23	3057	21465
9	B,9	038_C10_CWR22Rv1.fsa	D10S1248	13	272.2	2470	18975
10	B,10	038_C10_CWR22Rv1.fsa	D10S1248	15	280.1	2681	20257
11	B,11	038_C10_CWR22Rv1.fsa	D13S317	9	316.35	3049	24467
12	B,12	038_C10_CWR22Rv1.fsa	D13S317	12	328.41	2266	18321
13	B,13	038_C10_CWR22Rv1.fsa	Penta E	5	368.91	2493	21249
14	B,14	038_C10_CWR22Rv1.fsa	Penta E	13	407.53	1811	15844
15	G,1	038_C10_CWR22Rv1.fsa	D16S539	12	111.1	12108	74855
16	G,2	038_C10_CWR22Rv1.fsa	D18S51	13	158.18	7147	44421
17	G,3	038_C10_CWR22Rv1.fsa	D18S51	14	162.43	6411	39037
18	G,4	038_C10_CWR22Rv1.fsa	D2S1338	17	251.79	4182	28711
19	G,5	038_C10_CWR22Rv1.fsa	D2S1338	18	255.9	3666	24842
20	G,6	038_C10_CWR22Rv1.fsa	CSF1PO	10	337.11	2535	19579
21	G,7	038_C10_CWR22Rv1.fsa	CSF1PO	11	341.16	2656	20533
22	G,8	038_C10_CWR22Rv1.fsa	Penta D	9	415.12	2278	19530
23	G,9	038_C10_CWR22Rv1.fsa	Penta D	12	430.81	2446	21217
24	Y,1	038_C10_CWR22Rv1.fsa	TH01	6	81.27	937	6375
25	Y,2	038_C10_CWR22Rv1.fsa	TH01	9.3	97.73	1111	6899
26	Y,3	038_C10_CWR22Rv1.fsa	vWA	15	148.63	5480	34761
27	Y,4	038_C10_CWR22Rv1.fsa	vWA	21	173.23	2161	13788
28	Y,5	038_C10_CWR22Rv1.fsa	D21S11	30	229.81	5711	38119
29	Y,6	038_C10_CWR22Rv1.fsa	D7S820	9	288.74	2298	16451
30	Y,7	038_C10_CWR22Rv1.fsa	D7S820	10	292.79	2877	20612
31	Y,8	038_C10_CWR22Rv1.fsa	D7S820	11	296.83	2417	17320
32	Y,9	038_C10_CWR22Rv1.fsa	D5S818	11	344.93	1290	10278
33	Y,10	038_C10_CWR22Rv1.fsa	D5S818	13	352.95	1173	9169
34	Y,11	038_C10_CWR22Rv1.fsa	TPOX	8	412.05	1668	14135
35	Y,12	038_C10_CWR22Rv1.fsa	DYS391	11	473.17	1040	9764
36	R,1	038_C10_CWR22Rv1.fsa	D8S1179	13	101.16	2174	14136

37	R,2	038_C10_CWR22Rv1.fsa	D8S1179	14	105.76	2501	16161
38	R,3	038_C10_CWR22Rv1.fsa	D12S391	18	154.38	7885	49452
39	R,4	038_C10_CWR22Rv1.fsa	D19S433	13	224.62	661	4307
40	R,5	038_C10_CWR22Rv1.fsa	D19S433	14	228.61	993	6628
41	R,6	038_C10_CWR22Rv1.fsa	FGA	20	291.06	1424	10296
42	R,7	038_C10_CWR22Rv1.fsa	FGA	23	302.45	1229	9116
43	R,8	038_C10_CWR22Rv1.fsa	D22S1045	15	456.34	2100	18216

The comparison between case result and STR profile database:

Cell Name	Locus name / STR Repeat Number									Match (%)	STR Profile Database*
	D5S818	D13S317	D7S820	D16S539	VWA	TH01	AM	TPOX	CSF1PO		
Test Sample	11,13	9,12	9,10,11	12,12	15,21	6,9.3	X,Y	8,8	10,11		
22Rv1	11,13	9,12	9,10,11	12,12	15,21	6,9.3	X,Y	8,8	10,11	100	ExPASy
CWR22Rv1-AR-EK	11,12	9,12	10,11	12,12	15,21	6,9.3	X,Y	8,8	10,11	89.66	ExPASy
22Rv1	11,12	8,9,12	10,11	12,12	14,15,20,21	6,9.3	X,Y	8,8	10,11	81.25	ExPASy
CWR22Pc	11,12	8,12	9,10,11	12,12	15,18	6,9.3	X,Y	8,8	10,11	80	ExPASy

* Online STR Analysis is performed on some bioresource websites as below:

https://web.expasy.org/cellosaurus-str-search/ (ExPASy)

https://www.atcc.org/STR_Database.aspx (ATCC)

https://www.dsmz.de/services/services-human-and-animal-cell-lines/online-str-analysis.html (DSMZ)

* This table just shows the top 10 cell lines within database those matches are ≥80%, and if there isn't any match ≥80%, it just shows the cell line which scores highest match with the test sample. If necessary, please utilize the online STR analysis on ATCC/DSMZ website for detailed comparison.

The interpretation of comparison:

Depending on the comparison, the highest 100% match is between the test sample and 22Rv1 cell line within database, and there are other three cell lines those matches are ≥80%.

Referring to ASN-0002-2011 (Authentication of Human Cell Lines: Standardization of STR Profiling), Cell line samples matching at ≥80% of alleles across the eight (8) core loci are said to be related, allowing authentication of the sample being tested. STR profiles are said to be unrelated when they match at <55% of alleles. STR profiles matching at 55-80% of alleles may be related and warrant further investigation as there may be overlap with a small number of cell lines that show marked genetic instability and slip below the 80% threshold.

- ***This STR analysis service is for research purposes only, and any medical usage is not allowed.***

GeneLabs Life Science Innovation
勁因科技有限公司

Genelabs Life science Corporation

www.genelabs.com.tw

Phone:(02)26557678, (04)22633813, (06)2094380

Freecall:0800-231914, 0800-094380

REPORT DOWNLOAD

Cell Line DNA Typing Report

Case Number: CID20220073

Report Date: 08/24/2022

Genelabs Life Science Corp.
12F.-6, No.3, Yuanqu St.,
Nangang Dist.,
Taipei City 115, Taiwan
TEL: +886-2-26557678
FAX: +886-2-26557572
E-mail: cellid@genelabs.com.tw

Sample Information:

i. Applicant Name: 王齡玉 Ling-Yu Wang

ii. Institution: 長庚大學生化科 Department of Biochemistry and Molecular Biology, Chang Gung University, Taoyuan, Taiwan

iii. Sample Description: VCaP

iv. Sample type: Cell Pellet

v. Sample Received Date: 08/12/2022

Test Description:

DNA of the sample is extracted by Roche MagNA Pure Compact System.

DNA conc. = 85.9 ng/μl; OD260/280 = 1.99 ; OD260/230 = 1.95

The STR loci are amplified by Promega GenePrint® 24 System.

The CE analysis is performed on ABI PRISM 3730 GENETIC ANALYZER.

The raw data is analyzed by GeneMapper® Software V3.7.

The STR analysis is operated and reported by Mission Biotech.

This report is issued by:

Liang Kuei Chang — Laboratory Director

James Chung — General Manager

STR Analysis Result:

ANSI/ATCC ASN-0002 STR Loci	Repeat Numbers	Extended STR Loci	Repeat Numbers	Extended STR Loci	Repeat Numbers
**D5S818**	12,12	D3S1358	14,15	DYS391*	11
**D13S317**	11,12	D1S1656	13,17.3	D8S1179	12,13
**D7S820**	9,12	D2S441	11,14	D12S391	21,23
**D16S539**	9,9	D10S1248	13,14	D19S433	13,13
**vWA**	18,19	Penta E	10,12	FGA	21,26
**TH01**	9.3,9.3	D18S51	13,13	D22S1045	16,17
**Amelogenin**	X,Y	D2S1338	17,25	Case Number: CID20220073<br>Test Date: 08/18/2022	
**TPOX**	8,11	Penta D	9,9		
**CSF1PO**	10,12	D21S11	31,31		

Allele Report

Case Number: CID20220073

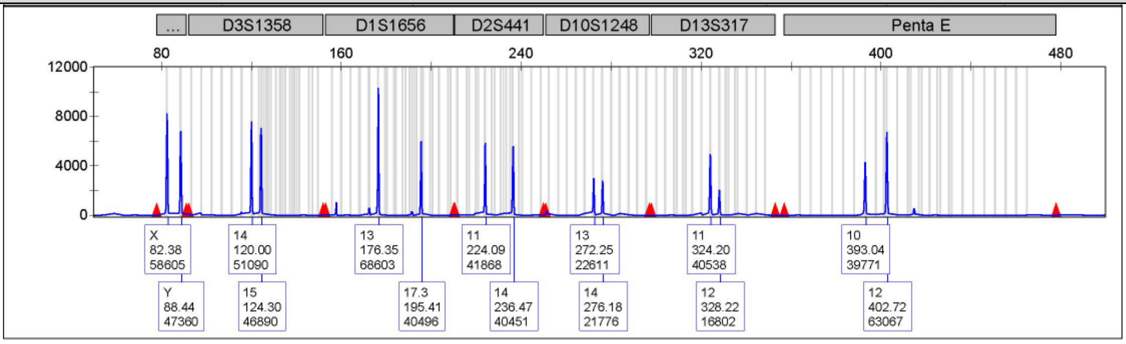

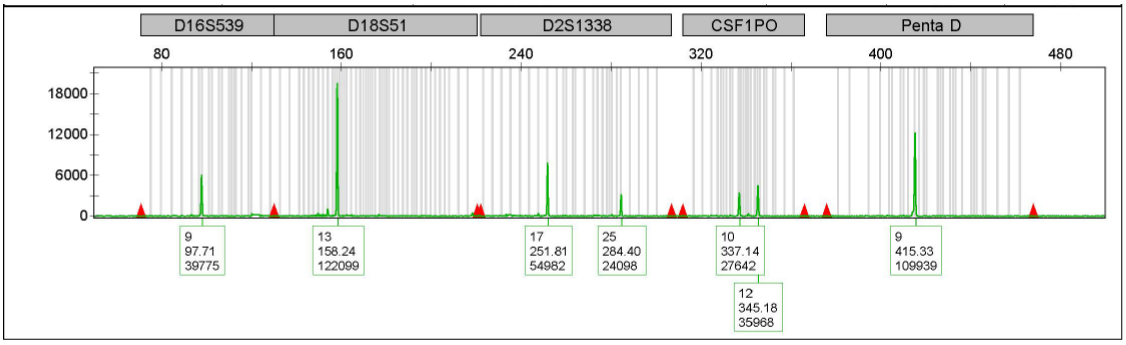

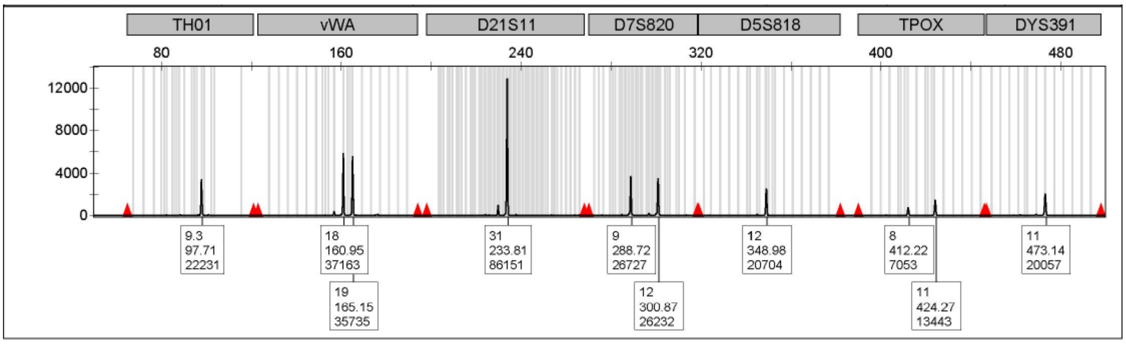

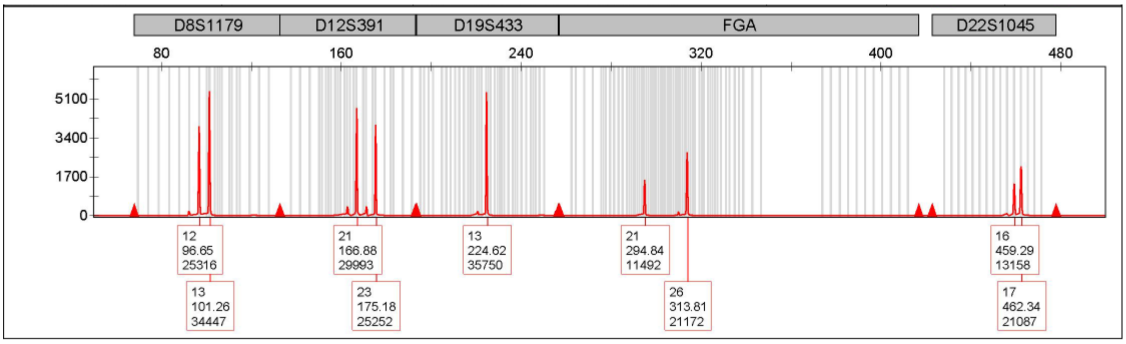

	Dye	Sample File Name	Marker	Allele	Size	Height	Area
1	B,1	037_D10_VCaP.fsa	AMEL	X	82.38	8290	58605
2	B,2	037_D10_VCaP.fsa	AMEL	Y	88.44	6839	47360
3	B,3	037_D10_VCaP.fsa	D3S1358	14	120	7619	51090
4	B,4	037_D10_VCaP.fsa	D3S1358	15	124.3	7079	46890
5	B,5	037_D10_VCaP.fsa	D1S1656	13	176.35	10346	68603
6	B,6	037_D10_VCaP.fsa	D1S1656	17.3	195.41	6031	40496
7	B,7	037_D10_VCaP.fsa	D2S441	11	224.09	5837	41868
8	B,8	037_D10_VCaP.fsa	D2S441	14	236.47	5619	40451
9	B,9	037_D10_VCaP.fsa	D10S1248	13	272.25	3001	22611
10	B,10	037_D10_VCaP.fsa	D10S1248	14	276.18	2814	21776
11	B,11	037_D10_VCaP.fsa	D13S317	11	324.2	4975	40538
12	B,12	037_D10_VCaP.fsa	D13S317	12	328.22	2004	16802
13	B,13	037_D10_VCaP.fsa	Penta E	10	393.04	4323	39771
14	B,14	037_D10_VCaP.fsa	Penta E	12	402.72	6752	63067
15	G,1	037_D10_VCaP.fsa	D16S539	9	97.71	6159	39775
16	G,2	037_D10_VCaP.fsa	D18S51	13	158.24	19649	122099
17	G,3	037_D10_VCaP.fsa	D2S1338	17	251.81	7964	54982
18	G,4	037_D10_VCaP.fsa	D2S1338	25	284.4	3204	24098
19	G,5	037_D10_VCaP.fsa	CSF1PO	10	337.14	3439	27642
20	G,6	037_D10_VCaP.fsa	CSF1PO	12	345.18	4452	35968
21	G,7	037_D10_VCaP.fsa	Penta D	9	415.33	12370	109939
22	Y,1	037_D10_VCaP.fsa	TH01	9.3	97.71	3463	22231
23	Y,2	037_D10_VCaP.fsa	vWA	18	160.95	5886	37163
24	Y,3	037_D10_VCaP.fsa	vWA	19	165.15	5676	35735
25	Y,4	037_D10_VCaP.fsa	D21S11	31	233.81	12961	86151
26	Y,5	037_D10_VCaP.fsa	D7S820	9	288.72	3765	26727
27	Y,6	037_D10_VCaP.fsa	D7S820	12	300.87	3584	26232
28	Y,7	037_D10_VCaP.fsa	D5S818	12	348.98	2565	20704
29	Y,8	037_D10_VCaP.fsa	TPOX	8	412.22	763	7053
30	Y,9	037_D10_VCaP.fsa	TPOX	11	424.27	1467	13443
31	Y,10	037_D10_VCaP.fsa	DYS391	11	473.14	2075	20057
32	R,1	037_D10_VCaP.fsa	D8S1179	12	96.65	3935	25316
33	R,2	037_D10_VCaP.fsa	D8S1179	13	101.26	5466	34447
34	R,3	037_D10_VCaP.fsa	D12S391	21	166.88	4687	29993
35	R,4	037_D10_VCaP.fsa	D12S391	23	175.18	3986	25252
36	R,5	037_D10_VCaP.fsa	D19S433	13	224.62	5410	35750

37	R,6	037_D10_VCaP.fsa	FGA	21	294.84	1573	11492
38	R,7	037_D10_VCaP.fsa	FGA	26	313.81	2791	21172
39	R,8	037_D10_VCaP.fsa	D22S1045	16	459.29	1411	13158
40	R,9	037_D10_VCaP.fsa	D22S1045	17	462.34	2210	21087

The comparison between case result and STR profile database:

Cell Name	Locus name / STR Repeat Number									Match (%)	STR Profile Database*
	D5S818	D13S317	D7S820	D16S539	VWA	TH01	AM	TPOX	CSF1PO		
Test Sample	12,12	11,12	9,12	9,9	18,19	9.3,9.3	X,Y	8,11	10,12		
VCaP	12,12	11,12	9,12	9,9	18,19	9.3,9.3	X,Y	8,11	10,12	100	ExPASy
chHES-85	10,12	11,12	9,12	9,10	18,19	9,9	X,X	8,11	12,12	81.48	ExPASy

* Online STR Analysis is performed on some bioresource websites as below:

https://web.expasy.org/cellosaurus-str-search/ (ExPASy)

https://www.atcc.org/STR_Database.aspx (ATCC)

https://www.dsmz.de/services/services-human-and-animal-cell-lines/online-str-analysis.html (DSMZ)

* This table just shows the top 10 cell lines within database those matches are ≥80%, and if there isn't any match ≥80%, it just shows the cell line which scores highest match with the test sample. If necessary, please utilize the online STR analysis on ATCC/DSMZ website for detailed comparison.

The interpretation of comparison:

Depending on the comparison, the highest 100% match is between the test sample and VCaP cell line within database, and there is another cell line that match is ≥80%.

Referring to ASN-0002-2011 (Authentication of Human Cell Lines: Standardization of STR Profiling), Cell line samples matching at ≥80% of alleles across the eight (8) core loci are said to be related, allowing authentication of the sample being tested. STR profiles are said to be unrelated when they match at <55% of alleles. STR profiles matching at 55-80% of alleles may be related and warrant further investigation as there may be overlap with a small number of cell lines that show marked genetic instability and slip below the 80% threshold.

- ***This STR analysis service is for research purposes only, and any medical usage is not allowed.***

勁因科技有限公司

Genelabs Life science Corporation

www.genelabs.com.tw

Phone:(02)26557678, (04)22633813, (06)2094380

Freecall:0800-231914, 0800-094380

REPORT DOWNLOAD

Cell Line DNA Typing Report

Case Number: CID20220074

Report Date: 08/24/2022

Genelabs Life Science Corp.
12F.-6, No.3, Yuanqu St.,
Nangang Dist.,
Taipei City 115, Taiwan
TEL: +886-2-26557678
FAX: +886-2-26557572
E-mail: cellid@genelabs.com.tw

Sample Information:

i. Applicant Name: 王齡玉 Ling-Yu Wang

ii. Institution: 長庚大學生化科 Department of Biochemistry and Molecular Biology, Chang Gung University, Taoyuan, Taiwan

iii. Sample Description: C4-2B

iv. Sample type: Cell Pellet

v. Sample Received Date: 08/12/2022

Test Description:

DNA of the sample is extracted by Roche MagNA Pure Compact System.

DNA conc. = 142.7 ng/μl; OD260/280 = 1.99 ; OD260/230 = 2.04

The STR loci are amplified by Promega GenePrint® 24 System.

The CE analysis is performed on ABI PRISM 3730 GENETIC ANALYZER.

The raw data is analyzed by GeneMapper® Software V3.7.

The STR analysis is operated and reported by Mission Biotech.

This report is issued by:

Liang Kuei Chang — Laboratory Director

James Chung — General Manager

STR Analysis Result:

ANSI/ATCC ASN-0002 STR Loci	Repeat Numbers	Extended STR Loci	Repeat Numbers	Extended STR Loci	Repeat Numbers
**D5S818**	11,12	D3S1358	15,16	DYS391*	N/A
**D13S317**	10,11	D1S1656	14,16,17	D8S1179	12,14
**D7S820**	9.1,10.3	D2S441	11.3,11.3	D12S391	20,21,22,23
**D16S539**	10,11	D10S1248	14,14	D19S433	13.2,14
**vWA**	16,18	Penta E	11,12,16,17	FGA	19,20
**TH01**	9,9	D18S51	11,12	D22S1045	15,16,17
**Amelogenin**	X,X	D2S1338	16,17	Case Number: CID20220074<br>Test Date: 08/18/2022	
**TPOX**	8,9	Penta D	12,13,14		
**CSF1PO**	9,10,11	D21S11	29,32.2		

Allele Report

Case Number: CID20220074

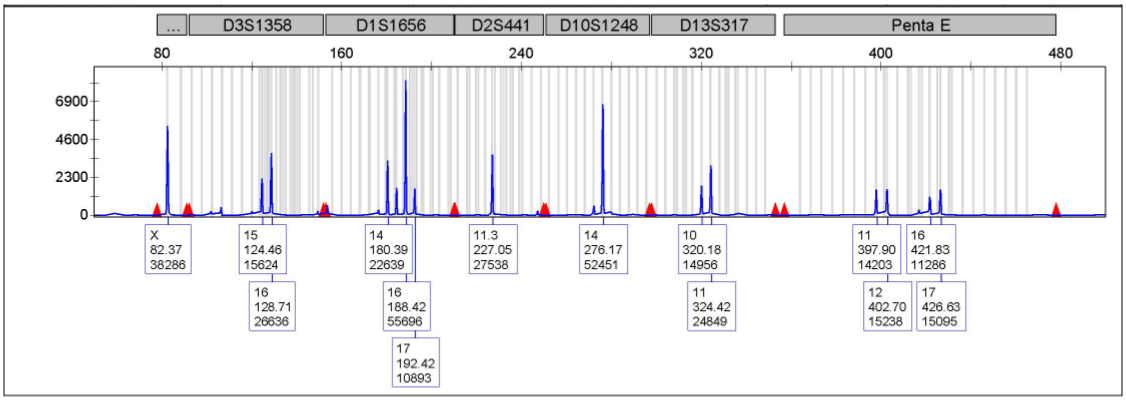

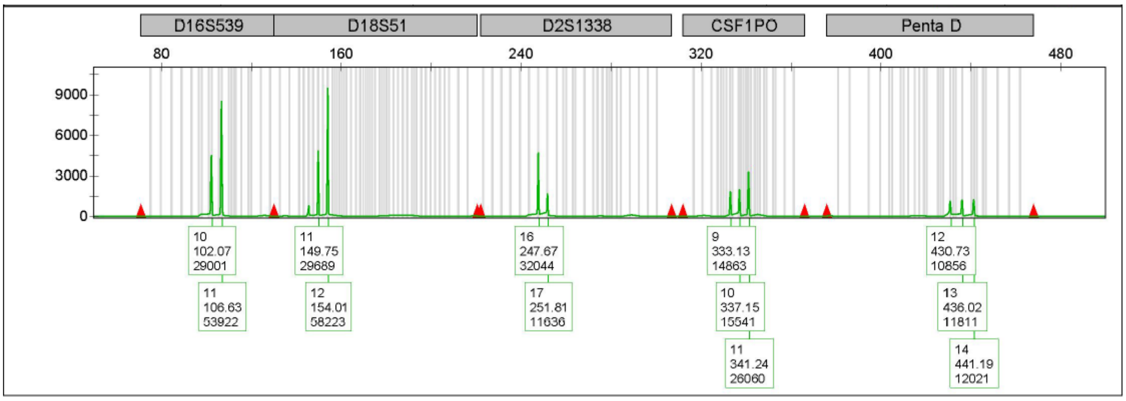

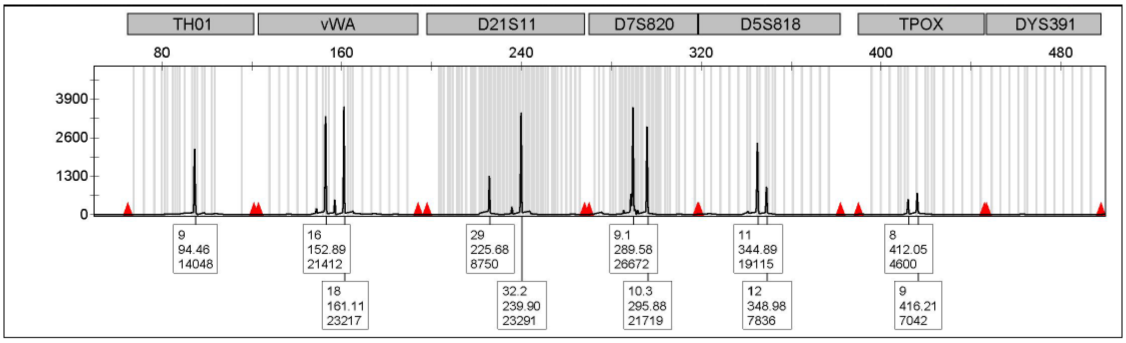

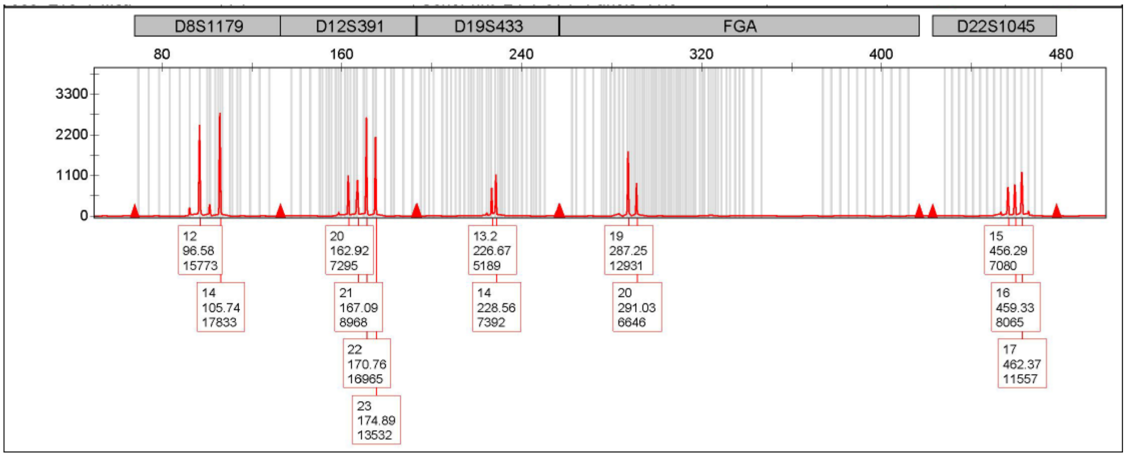

	Dye	Sample File Name	Marker	Allele	Size	Height	Area
1	B,1	036_E10_C4-2B.fsa	AMEL	X	82.37	5458	38286
2	B,2	036_E10_C4-2B.fsa	D3S1358	15	124.46	2236	15624
3	B,3	036_E10_C4-2B.fsa	D3S1358	16	128.71	3788	26636
4	B,4	036_E10_C4-2B.fsa	D1S1656	14	180.39	3350	22639
5	B,5	036_E10_C4-2B.fsa	D1S1656	16	188.42	8237	55696
6	B,6	036_E10_C4-2B.fsa	D1S1656	17	192.42	1652	10893
7	B,7	036_E10_C4-2B.fsa	D2S441	11.3	227.05	3687	27538
8	B,8	036_E10_C4-2B.fsa	D10S1248	14	276.17	6755	52451
9	B,9	036_E10_C4-2B.fsa	D13S317	10	320.18	1792	14956
10	B,10	036_E10_C4-2B.fsa	D13S317	11	324.42	3034	24849
11	B,11	036_E10_C4-2B.fsa	Penta E	11	397.9	1546	14203
12	B,12	036_E10_C4-2B.fsa	Penta E	12	402.7	1628	15238
13	B,13	036_E10_C4-2B.fsa	Penta E	16	421.83	1186	11286
14	B,14	036_E10_C4-2B.fsa	Penta E	17	426.63	1603	15095
15	G,1	036_E10_C4-2B.fsa	D16S539	10	102.07	4559	29001
16	G,2	036_E10_C4-2B.fsa	D16S539	11	106.63	8591	53922
17	G,3	036_E10_C4-2B.fsa	D18S51	11	149.75	4871	29689
18	G,4	036_E10_C4-2B.fsa	D18S51	12	154.01	9543	58223
19	G,5	036_E10_C4-2B.fsa	D2S1338	16	247.67	4754	32044
20	G,6	036_E10_C4-2B.fsa	D2S1338	17	251.81	1671	11636
21	G,7	036_E10_C4-2B.fsa	CSF1PO	9	333.13	1909	14863
22	G,8	036_E10_C4-2B.fsa	CSF1PO	10	337.15	2009	15541
23	G,9	036_E10_C4-2B.fsa	CSF1PO	11	341.24	3311	26060
24	G,10	036_E10_C4-2B.fsa	Penta D	12	430.73	1208	10856
25	G,11	036_E10_C4-2B.fsa	Penta D	13	436.02	1294	11811
26	G,12	036_E10_C4-2B.fsa	Penta D	14	441.19	1334	12021
27	Y,1	036_E10_C4-2B.fsa	TH01	9	94.46	2236	14048
28	Y,2	036_E10_C4-2B.fsa	vWA	16	152.89	3342	21412
29	Y,3	036_E10_C4-2B.fsa	vWA	18	161.11	3645	23217
30	Y,4	036_E10_C4-2B.fsa	D21S11	29	225.68	1321	8750
31	Y,5	036_E10_C4-2B.fsa	D21S11	32.2	239.9	3442	23291
32	Y,6	036_E10_C4-2B.fsa	D7S820	9.1	289.58	3618	26672
33	Y,7	036_E10_C4-2B.fsa	D7S820	10.3	295.88	2989	21719
34	Y,8	036_E10_C4-2B.fsa	D5S818	11	344.89	2434	19115
35	Y,9	036_E10_C4-2B.fsa	D5S818	12	348.98	960	7836
36	Y,10	036_E10_C4-2B.fsa	TPOX	8	412.05	535	4600

37	Y,11	036_E10_C4-2B.fsa	TPOX	9	416.21	766	7042
38	R,1	036_E10_C4-2B.fsa	D8S1179	12	96.58	2476	15773
39	R,2	036_E10_C4-2B.fsa	D8S1179	14	105.74	2805	17833
40	R,3	036_E10_C4-2B.fsa	D12S391	20	162.92	1113	7295
41	R,4	036_E10_C4-2B.fsa	D12S391	21	167.09	986	8968
42	R,5	036_E10_C4-2B.fsa	D12S391	22	170.76	2673	16965
43	R,6	036_E10_C4-2B.fsa	D12S391	23	174.89	2160	13532
44	R,7	036_E10_C4-2B.fsa	D19S433	13.2	226.67	771	5189
45	R,8	036_E10_C4-2B.fsa	D19S433	14	228.56	1130	7392
46	R,9	036_E10_C4-2B.fsa	FGA	19	287.25	1758	12931
47	R,10	036_E10_C4-2B.fsa	FGA	20	291.03	900	6646
48	R,11	036_E10_C4-2B.fsa	D22S1045	15	456.29	794	7080
49	R,12	036_E10_C4-2B.fsa	D22S1045	16	459.33	883	8065
50	R,13	036_E10_C4-2B.fsa	D22S1045	17	462.37	1196	11557

The comparison between case result and STR profile database:

Cell Name	Locus name / STR Repeat Number									Match (%)	STR Profile Database*
	D5S818	D13S317	D7S820	D16S539	VWA	TH01	AM	TPOX	CSF1PO		
Test Sample	11,12	10,11	9.1,10.3	10,11	16,18	9,9	X,X	8,9	9,10,11		
LNCaP C4-2B	11,12	10,11	8.1,9.1,10.3	10,11	16,18	9,9	X,Y	8,9	9,10,11	96.97	ExPASy
LNCaP	11,12	10,11,12,13	9.1,10.3	11,11	16,18	9,9	X,X	8,9	10,11	87.5	ExPASy
LNCaP C4	11,12	10,11	9,9.1,10.3	11,11	16,18,19	9,9	X,Y	8,9	10,11	87.5	ExPASy

* Online STR Analysis is performed on some bioresource websites as below:

https://web.expasy.org/cellosaurus-str-search/ (ExPASy)

https://www.atcc.org/STR_Database.aspx (ATCC)

https://www.dsmz.de/services/services-human-and-animal-cell-lines/online-str-analysis.html (DSMZ)

* This table just shows the top 10 cell lines within database those matches are ≥80%, and if there isn't any match ≥80%, it just shows the cell line which scores highest match with the test sample. If necessary, please utilize the online STR analysis on ATCC/DSMZ website for detailed comparison.

The interpretation of comparison:

Depending on the comparison, the highest 96.97% match is between the test sample and LNCaP C4-2B cell line within database, and there are other two cell lines those matches are ≥80%.

Referring to ASN-0002-2011 (Authentication of Human Cell Lines: Standardization of STR Profiling), Cell line samples matching at ≥80% of alleles across the eight (8) core loci are said to be related, allowing authentication of the sample being tested. STR profiles are said to be unrelated when they match at <55% of alleles. STR profiles matching at 55-80% of alleles may be related and warrant further investigation as there may be overlap with a small number of cell lines that show marked genetic instability and slip below the 80% threshold.

- ***This STR analysis service is for research purposes only, and any medical usage is not allowed.***

Genelabs Life science Corporation

www.genelabs.com.tw

Phone:(02)26557678, (04)22633813, (06)2094380

Freecall:0800-231914, 0800-094380

REPORT DOWNLOAD

Cell Line DNA Typing Report

Case Number: CID20220075

Report Date: 08/24/2022

Genelabs Life Science Corp.
12F.-6, No.3, Yuanqu St.,
Nangang Dist.,
Taipei City 115, Taiwan
TEL: +886-2-26557678
FAX: +886-2-26557572
E-mail: cellid@genelabs.com.tw

Sample Information:

i. Applicant Name: 王齡玉 Ling-Yu Wang

ii. Institution: 長庚大學生化科 Department of Biochemistry and Molecular Biology, Chang Gung University, Taoyuan, Taiwan

iii. Sample Description: C4-2B/EnzR

iv. Sample type: Cell Pellet

v. Sample Received Date: 08/12/2022

Test Description:

DNA of the sample is extracted by Roche MagNA Pure Compact System.

DNA conc. = 111.3 ng/μl; OD260/280 = 2.01 ; OD260/230 = 1.97

The STR loci are amplified by Promega GenePrint® 24 System.

The CE analysis is performed on ABI PRISM 3730 GENETIC ANALYZER.

The raw data is analyzed by GeneMapper® Software V3.7.

The STR analysis is operated and reported by Mission Biotech.

This report is issued by:

Laboratory Director   General Manager

STR Analysis Result:

**ANSI/ATCC ASN-0002 STR Loci**	**Repeat Numbers**	Extended STR Loci	Repeat Numbers	Extended STR Loci	Repeat Numbers
**D5S818**	11,12	D3S1358	15,16	DYS391*	N/A
**D13S317**	9,10,11	D1S1656	16,17	D8S1179	11,12,14
**D7S820**	9.1,10.3	D2S441	11.3,11.3	D12S391	21,22
**D16S539**	10,11	D10S1248	13,14,15	D19S433	13,15
**vWA**	15,16,18	Penta E	12,16,17,18	FGA	18,21
**TH01**	9,9	D18S51	9,12	D22S1045	16,17,18
**Amelogenin**	X,X	D2S1338	16,17	Case Number: CID20220075<br>Test Date: 08/18/2022	
**TPOX**	8,9	Penta D	12,13		
**CSF1PO**	9,10,11	D21S11	29,31.2,32.2		

Allele Report

Case Number: CID20220075

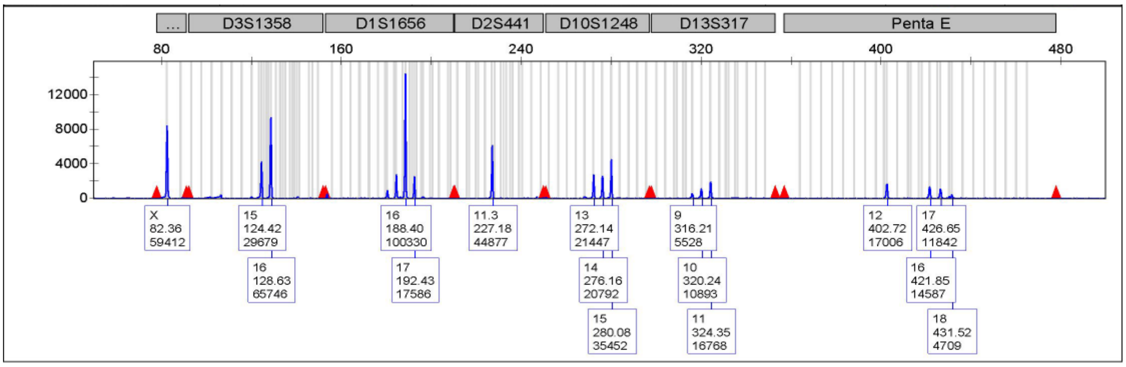

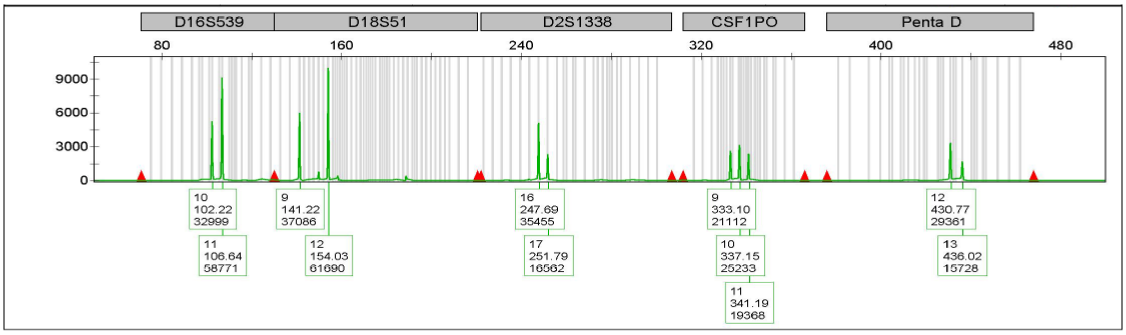

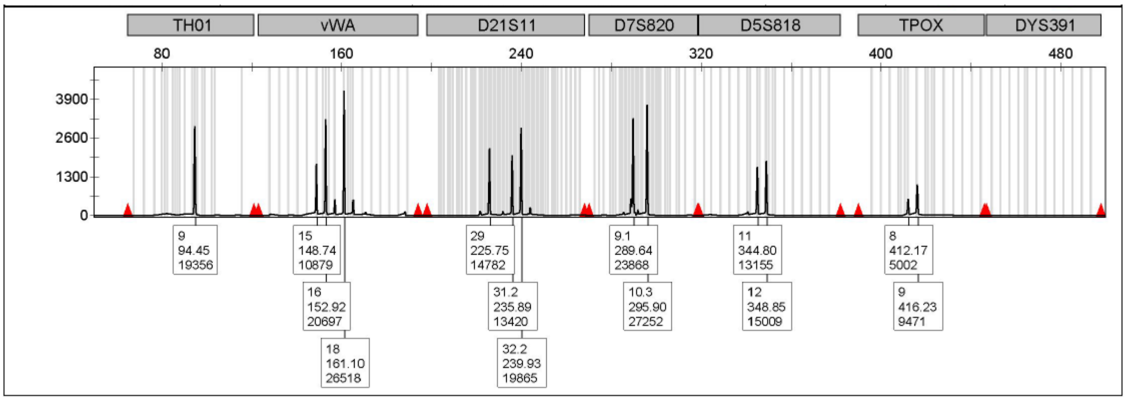

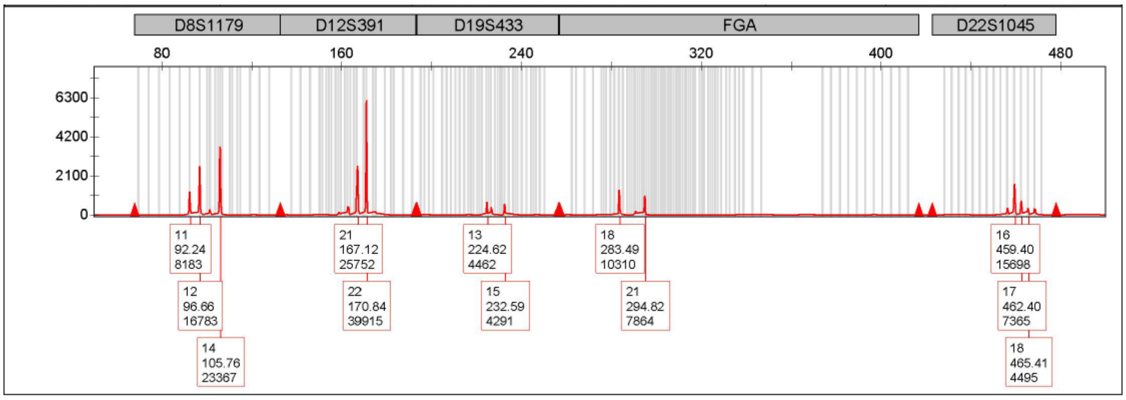

	Dye	Sample File Name	Marker	Allele	Size	Height	Area
1	B,1	035_F10_C4-2B/EnzR.fsa	AMEL	X	82.36	8436	59412
2	B,2	035_F10_C4-2B/EnzR.fsa	D3S1358	15	124.42	4211	29679
3	B,3	035_F10_C4-2B/EnzR.fsa	D3S1358	16	128.63	9414	65746
4	B,4	035_F10_C4-2B/EnzR.fsa	D1S1656	16	188.4	14423	100330
5	B,5	035_F10_C4-2B/EnzR.fsa	D1S1656	17	192.43	2543	17586
6	B,6	035_F10_C4-2B/EnzR.fsa	D2S441	11.3	227.18	6163	44877
7	B,7	035_F10_C4-2B/EnzR.fsa	D10S1248	13	272.14	2736	21447
8	B,8	035_F10_C4-2B/EnzR.fsa	D10S1248	14	276.16	2656	20792
9	B,9	035_F10_C4-2B/EnzR.fsa	D10S1248	15	280.08	4511	35452
10	B,10	035_F10_C4-2B/EnzR.fsa	D13S317	9	316.21	664	5528
11	B,11	035_F10_C4-2B/EnzR.fsa	D13S317	10	320.24	1292	10893
12	B,12	035_F10_C4-2B/EnzR.fsa	D13S317	11	324.35	2015	16768
13	B,13	035_F10_C4-2B/EnzR.fsa	Penta E	12	402.72	1861	17006
14	B,14	035_F10_C4-2B/EnzR.fsa	Penta E	16	421.85	1535	14587
15	B,15	035_F10_C4-2B/EnzR.fsa	Penta E	17	426.65	1228	11842
16	B,16	035_F10_C4-2B/EnzR.fsa	Penta E	18	431.52	549	4709
17	G,1	035_F10_C4-2B/EnzR.fsa	D16S539	10	102.22	5267	32999
18	G,2	035_F10_C4-2B/EnzR.fsa	D16S539	11	106.64	9196	58771
19	G,3	035_F10_C4-2B/EnzR.fsa	D18S51	9	141.22	6023	37086
20	G,4	035_F10_C4-2B/EnzR.fsa	D18S51	12	154.03	10049	61690
21	G,5	035_F10_C4-2B/EnzR.fsa	D2S1338	16	247.69	5114	35455
22	G,6	035_F10_C4-2B/EnzR.fsa	D2S1338	17	251.79	2372	16562
23	G,7	035_F10_C4-2B/EnzR.fsa	CSF1PO	9	333.1	2677	21112
24	G,8	035_F10_C4-2B/EnzR.fsa	CSF1PO	10	337.15	3180	25233
25	G,9	035_F10_C4-2B/EnzR.fsa	CSF1PO	11	341.19	2363	19368
26	G,10	035_F10_C4-2B/EnzR.fsa	Penta D	12	430.77	3299	29361
27	G,11	035_F10_C4-2B/EnzR.fsa	Penta D	13	436.02	1708	15728
28	Y,1	035_F10_C4-2B/EnzR.fsa	TH01	9	94.45	3019	19356
29	Y,2	035_F10_C4-2B/EnzR.fsa	vWA	15	148.74	1752	10879
30	Y,3	035_F10_C4-2B/EnzR.fsa	vWA	16	152.92	3247	20697
31	Y,4	035_F10_C4-2B/EnzR.fsa	vWA	18	161.1	4197	26518
32	Y,5	035_F10_C4-2B/EnzR.fsa	D21S11	29	225.75	2267	14782
33	Y,6	035_F10_C4-2B/EnzR.fsa	D21S11	31.2	235.89	2045	13420
34	Y,7	035_F10_C4-2B/EnzR.fsa	D21S11	32.2	239.93	2966	19865
35	Y,8	035_F10_C4-2B/EnzR.fsa	D7S820	9.1	289.64	3284	23868
36	Y,9	035_F10_C4-2B/EnzR.fsa	D7S820	10.3	295.9	3732	27252

37	Y,10	035_F10_C4-2B/EnzR.fsa	D5S818	11	344.8	1655	13155
38	Y,11	035_F10_C4-2B/EnzR.fsa	D5S818	12	348.85	1852	15009
39	Y,12	035_F10_C4-2B/EnzR.fsa	TPOX	8	412.17	578	5002
40	Y,13	035_F10_C4-2B/EnzR.fsa	TPOX	9	416.23	1042	9471
41	R,1	035_F10_C4-2B/EnzR.fsa	D8S1179	11	92.24	1255	8183
42	R,2	035_F10_C4-2B/EnzR.fsa	D8S1179	12	96.66	2624	16783
43	R,3	035_F10_C4-2B/EnzR.fsa	D8S1179	14	105.76	3688	23367
44	R,4	035_F10_C4-2B/EnzR.fsa	D12S391	21	167.12	2658	25752
45	R,5	035_F10_C4-2B/EnzR.fsa	D12S391	22	170.84	6181	39915
46	R,6	035_F10_C4-2B/EnzR.fsa	D19S433	13	224.62	691	4462
47	R,7	035_F10_C4-2B/EnzR.fsa	D19S433	15	232.59	624	4291
48	R,8	035_F10_C4-2B/EnzR.fsa	FGA	18	283.49	1373	10310
49	R,9	035_F10_C4-2B/EnzR.fsa	FGA	21	294.82	1104	7864
50	R,10	035_F10_C4-2B/EnzR.fsa	D22S1045	16	459.4	1689	15698
51	R,11	035_F10_C4-2B/EnzR.fsa	D22S1045	17	462.4	809	7365
52	R,12	035_F10_C4-2B/EnzR.fsa	D22S1045	18	465.41	484	4495

The comparison between case result and STR profile database:

Cell Name	Locus name / STR Repeat Number									Match (%)	STR Profile Database*
	D5S818	D13S317	D7S820	D16S539	VWA	TH01	AM	TPOX	CSF1PO		
Test Sample	11,12	9,10,11	9.1,10.3	10,11	15,16, 18	9,9	X,X	8,9	9,10,11		
LNCaP C4-2B	11,12	10,11	8.1,9.1, 10.3	10,11	16,18	9,9	X,Y	8,9	9,10,11	91.43	ExPASy
LNCaP	11,12	10,11, 12,13	9.1,10.3	11,11	16,18	9,9	X,X	8,9	10,11	82.35	ExPASy
LNCaP C4	11,12	10,11	9,9.1,10 .3	11,11	16,18, 19	9,9	X,Y	8,9	10,11	82.35	ExPASy
LNCaP clone FGC	11,12	10,12	9.1,10.3	11,11	16,18	9,9	X,Y	8,9	10,11	81.25	ExPASy
LNCaP clone FGC-Luc2	11,12	10,12	9.1,10.3	11,11	16,18	9,9	X,Y	8,9	10,11	81.25	ExPASy

* Online STR Analysis is performed on some bioresource websites as below:

https://web.expasy.org/cellosaurus-str-search/ (ExPASy)

https://www.atcc.org/STR_Database.aspx (ATCC)

https://www.dsmz.de/services/services-human-and-animal-cell-lines/online-str-analysis.html (DSMZ)

* This table just shows the top 10 cell lines within database those matches are ≥80%, and if there isn't any match ≥80%, it just shows the cell line which scores highest match with the test sample. If necessary, please utilize the online STR analysis on ATCC/DSMZ website for detailed comparison.

The interpretation of comparison:

Depending on the comparison, the highest 91.43% match is between the test sample and LNCaP C4-2B cell line within database, and there are other four cell lines those matches are ≥80%.

Referring to ASN-0002-2011 (Authentication of Human Cell Lines: Standardization of STR Profiling), Cell line samples matching at ≥80% of alleles across the eight (8) core loci are said to be related, allowing authentication of the sample being tested. STR profiles are said to be unrelated when they match at <55% of alleles. STR profiles matching at 55-80% of alleles may be related and warrant further investigation as there may be overlap with a small number of cell lines that show marked genetic instability and slip below the 80% threshold.

- ***This STR analysis service is for research purposes only, and any medical usage is not allowed.***

REPORT
DOWNLOAD

Genelabs Life science Corporation
www.genelabs.com.tw
Phone:(02)26557678, (04)22633813, (06)2094380
Freecall:0800-231914, 0800-094380

Cell Line DNA Typing Report

Case Number: CID20220076

Report Date: 08/24/2022

Genelabs Life Science Corp.
12F.-6, No.3, Yuanqu St.,
Nangang Dist.,
Taipei City 115, Taiwan
TEL: +886-2-26557678
FAX: +886-2-26557572
E-mail: cellid@genelabs.com.tw

Sample Information:

i. Applicant Name: 王齡玉 Ling-Yu Wang

ii. Institution: 長庚大學生化科 Department of Biochemistry and Molecular Biology, Chang Gung University, Taoyuan, Taiwan

iii. Sample Description: PNT2

iv. Sample type: Cell Pellet

v. Sample Received Date: 08/12/2022

Test Description:

DNA of the sample is extracted by Roche MagNA Pure Compact System.

DNA conc. = 98.0 ng/μl; OD260/280 = 1.97 ; OD260/230 = 2.05

The STR loci are amplified by Promega GenePrint® 24 System.

The CE analysis is performed on ABI PRISM 3730 GENETIC ANALYZER.

The raw data is analyzed by GeneMapper® Software V3.7.

The STR analysis is operated and reported by Mission Biotech.

This report is issued by:

Laboratory Director   General Manager

STR Analysis Result:

ANSI/ATCC ASN-0002 STR Loci	Repeat Numbers	Extended STR Loci	Repeat Numbers	Extended STR Loci	Repeat Numbers
**D5S818**	12,13	D3S1358	16,17	DYS391*	10
**D13S317**	8,13	D1S1656	16.3,17.3	D8S1179	13,14
**D7S820**	11,11	D2S441	10,12	D12S391	21,22
**D16S539**	9,9	D10S1248	14,14	D19S433	14,16
**vWA**	17,17	Penta E	5,12	FGA	22,22
**TH01**	6,9.3	D18S51	16,16	D22S1045	16,16
**Amelogenin**	X,Y	D2S1338	17,19	Case Number: CID20220076<br>Test Date: 08/18/2022	
**TPOX**	8,9	Penta D	9,15		
**CSF1PO**	11,13	D21S11	29,33.2		

Allele Report

Case Number: CID20220076

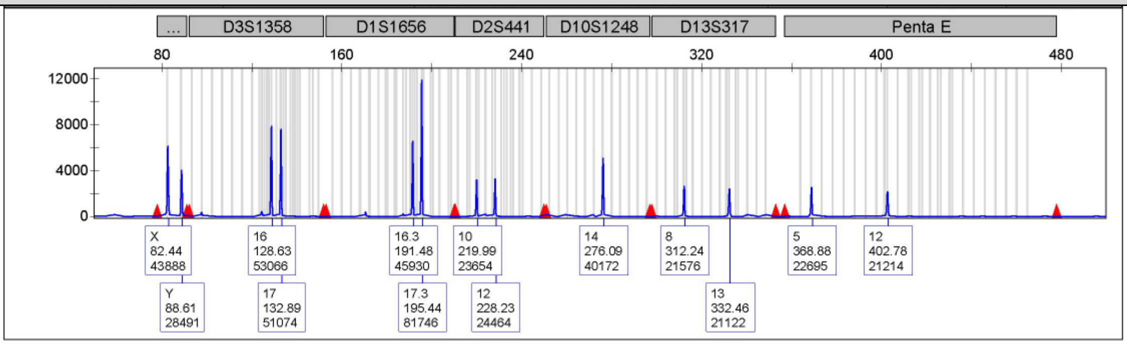

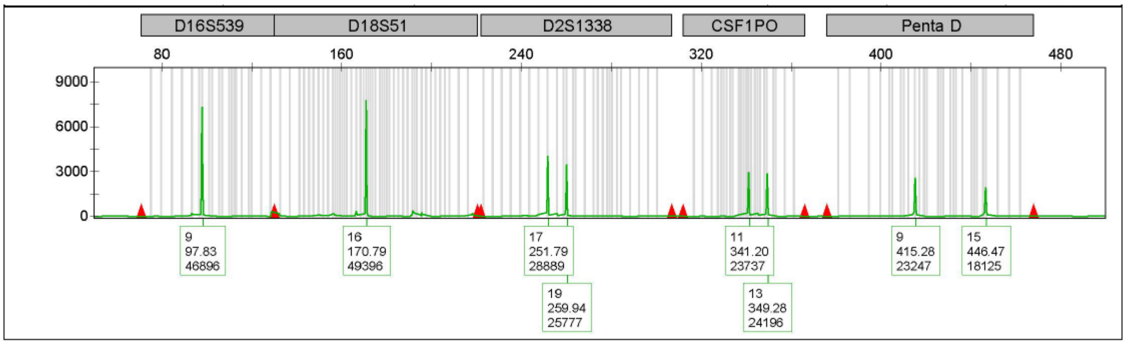

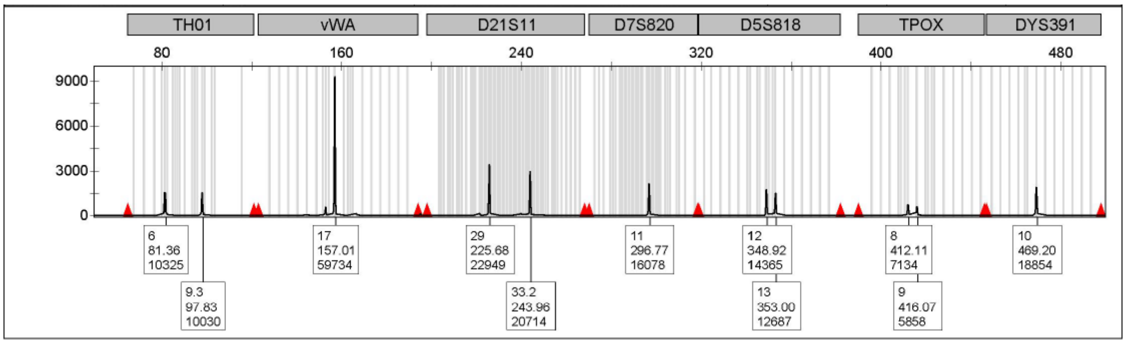

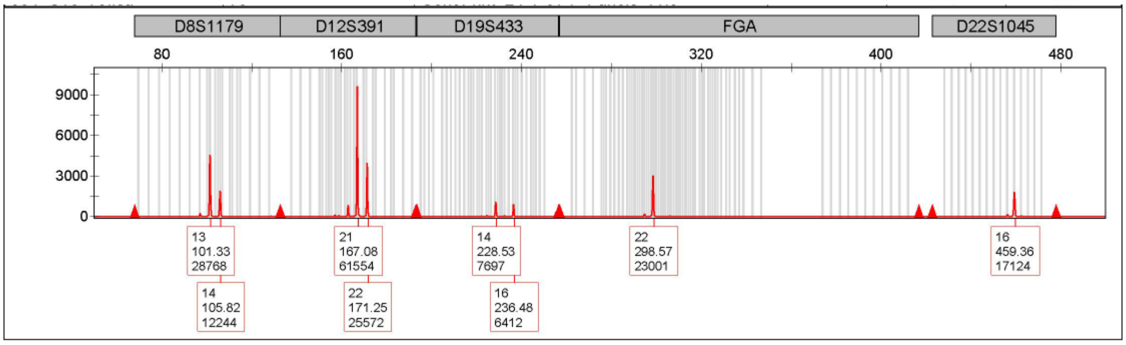

	Dye	Sample File Name	Marker	Allele	Size	Height	Area
1	B,1	034_G10_PNT2.fsa	AMEL	X	82.44	6158	43888
2	B,2	034_G10_PNT2.fsa	AMEL	Y	88.61	4088	28491
3	B,3	034_G10_PNT2.fsa	D3S1358	16	128.63	7951	53066
4	B,4	034_G10_PNT2.fsa	D3S1358	17	132.89	7693	51074
5	B,5	034_G10_PNT2.fsa	D1S1656	16.3	191.48	6622	45930
6	B,6	034_G10_PNT2.fsa	D1S1656	17.3	195.44	11939	81746
7	B,7	034_G10_PNT2.fsa	D2S441	10	219.99	3236	23654
8	B,8	034_G10_PNT2.fsa	D2S441	12	228.23	3284	24464
9	B,9	034_G10_PNT2.fsa	D10S1248	14	276.09	5132	40172
10	B,10	034_G10_PNT2.fsa	D13S317	8	312.24	2679	21576
11	B,11	034_G10_PNT2.fsa	D13S317	13	332.46	2466	21122
12	B,12	034_G10_PNT2.fsa	Penta E	5	368.88	2563	22695
13	B,13	034_G10_PNT2.fsa	Penta E	12	402.78	2218	21214
14	G,1	034_G10_PNT2.fsa	D16S539	9	97.83	7374	46896
15	G,2	034_G10_PNT2.fsa	D18S51	16	170.79	7783	49396
16	G,3	034_G10_PNT2.fsa	D2S1338	17	251.79	4063	28889
17	G,4	034_G10_PNT2.fsa	D2S1338	19	259.94	3520	25777
18	G,5	034_G10_PNT2.fsa	CSF1PO	11	341.2	2956	23737
19	G,6	034_G10_PNT2.fsa	CSF1PO	13	349.28	2914	24196
20	G,7	034_G10_PNT2.fsa	Penta D	9	415.28	2590	23247
21	G,8	034_G10_PNT2.fsa	Penta D	15	446.47	1948	18125
22	Y,1	034_G10_PNT2.fsa	TH01	6	81.36	1567	10325
23	Y,2	034_G10_PNT2.fsa	TH01	9.3	97.83	1576	10030
24	Y,3	034_G10_PNT2.fsa	vWA	17	157.01	9364	59734
25	Y,4	034_G10_PNT2.fsa	D21S11	29	225.68	3443	22949
26	Y,5	034_G10_PNT2.fsa	D21S11	33.2	243.96	3032	20714
27	Y,6	034_G10_PNT2.fsa	D7S820	11	296.77	2177	16078
28	Y,7	034_G10_PNT2.fsa	D5S818	12	348.92	1789	14365
29	Y,8	034_G10_PNT2.fsa	D5S818	13	353	1575	12687
30	Y,9	034_G10_PNT2.fsa	TPOX	8	412.11	804	7134
31	Y,10	034_G10_PNT2.fsa	TPOX	9	416.07	657	5858
32	Y,11	034_G10_PNT2.fsa	DYS391	10	469.2	1942	18854
33	R,1	034_G10_PNT2.fsa	D8S1179	13	101.33	4593	28768
34	R,2	034_G10_PNT2.fsa	D8S1179	14	105.82	1930	12244
35	R,3	034_G10_PNT2.fsa	D12S391	21	167.08	9658	61554
36	R,4	034_G10_PNT2.fsa	D12S391	22	171.25	4009	25572

37	R,5	034_G10_PNT2.fsa	D19S433	14	228.53	1098	7697
38	R,6	034_G10_PNT2.fsa	D19S433	16	236.48	933	6412
39	R,7	034_G10_PNT2.fsa	FGA	22	298.57	3020	23001
40	R,8	034_G10_PNT2.fsa	D22S1045	16	459.36	1808	17124

The comparison between case result and STR profile database:

Cell Name	Locus name / STR Repeat Number									Match (%)	STR Profile Database*
	D5S818	D13S317	D7S820	D16S539	VWA	TH01	AM	TPOX	CSF1PO		
Test Sample	12,13	8,13	11,11	9,9	17,17	6,9.3	X,Y	8,9	11,13		
PNT2	12,13	8,13	11,11	9,9	17,17	6,9.3	X,Y	8,9	11,13	100	ExPASy

* Online STR Analysis is performed on some bioresource websites as below:

https://web.expasy.org/cellosaurus-str-search/ (ExPASy)

https://www.atcc.org/STR_Database.aspx (ATCC)

https://www.dsmz.de/services/services-human-and-animal-cell-lines/online-str-analysis.html (DSMZ)

* This table just shows the top 10 cell lines within database those matches are ≥80%, and if there isn't any match ≥80%, it just shows the cell line which scores highest match with the test sample. If necessary, please utilize the online STR analysis on ATCC/DSMZ website for detailed comparison.

The interpretation of comparison:

Depending on the comparison, the highest 100% match is between the test sample and PNT2 cell line within database.

Referring to ASN-0002-2011 (Authentication of Human Cell Lines: Standardization of STR Profiling), Cell line samples matching at ≥80% of alleles across the eight (8) core loci are said to be related, allowing authentication of the sample being tested. STR profiles are said to be unrelated when they match at <55% of alleles. STR profiles matching at 55-80% of alleles may be related and warrant further investigation as there may be overlap with a small number of cell lines that show marked genetic instability and slip below the 80% threshold.

- ***This STR analysis service is for research purposes only, and any medical usage is not allowed.***

GeneLabs Life Science Innovation
勁因科技有限公司

Genelabs Life science Corporation

www.genelabs.com.tw

Phone:(02)26557678, (04)22633813, (06)2094380

Freecall:0800-231914, 0800-094380

REPORT DOWNLOAD

Cell Line DNA Typing Report

Case Number: CID20220077

Report Date: 08/24/2022

Genelabs Life Science Corp.
12F.-6, No.3, Yuanqu St.,
Nangang Dist.,
Taipei City 115, Taiwan
TEL: +886-2-26557678
FAX: +886-2-26557572
E-mail: cellid@genelabs.com.tw

Sample Information:

i. Applicant Name: 王齡玉 Ling-Yu Wang

ii. Institution: 長庚大學生化科 Department of Biochemistry and Molecular Biology, Chang Gung University, Taoyuan, Taiwan

iii. Sample Description: HEK293T

iv. Sample type: Cell Pellet

v. Sample Received Date: 08/12/2022

Test Description:

DNA of the sample is extracted by Roche MagNA Pure Compact System.

DNA conc. = 89.4 ng/µl; OD260/280 = 2.00 ; OD260/230 = 1.92

The STR loci are amplified by Promega GenePrint® 24 System.

The CE analysis is performed on ABI PRISM 3730 GENETIC ANALYZER.

The raw data is analyzed by GeneMapper® Software V3.7.

The STR analysis is operated and reported by Mission Biotech.

This report is issued by:

Liang Kuei Chang — Laboratory Director

James Chung — General Manager

STR Analysis Result:

ANSI/ATCC ASN-0002 STR Loci	Repeat Numbers	Extended STR Loci	Repeat Numbers	Extended STR Loci	Repeat Numbers
D5S818	8,9	D3S1358	15,16,17	DYS391*	N/A
D13S317	12,13	D1S1656	14,15,16,17,17.3	D8S1179	11,12,16
D7S820	11,11	D2S441	11,15	D12S391	19,20,21,22
D16S539	9,13	D10S1248	13,14	D19S433	16,17,19
vWA	16,19,20,21	Penta E	7,15	FGA	23,24
TH01	7,9.3	D18S51	16,17,18	D22S1045	16,17
Amelogenin	X,X	D2S1338	18,19	Case Number: CID20220077<br>Test Date: 08/18/2022	
TPOX	11,11	Penta D	9,10		
CSF1PO	10,11,12	D21S11	28,29,31.2		

Allele Report

Case Number: CID20220077

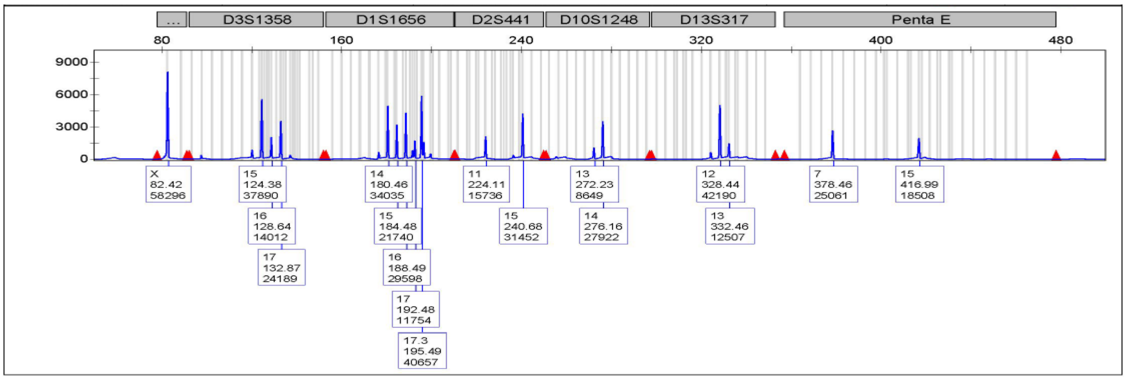

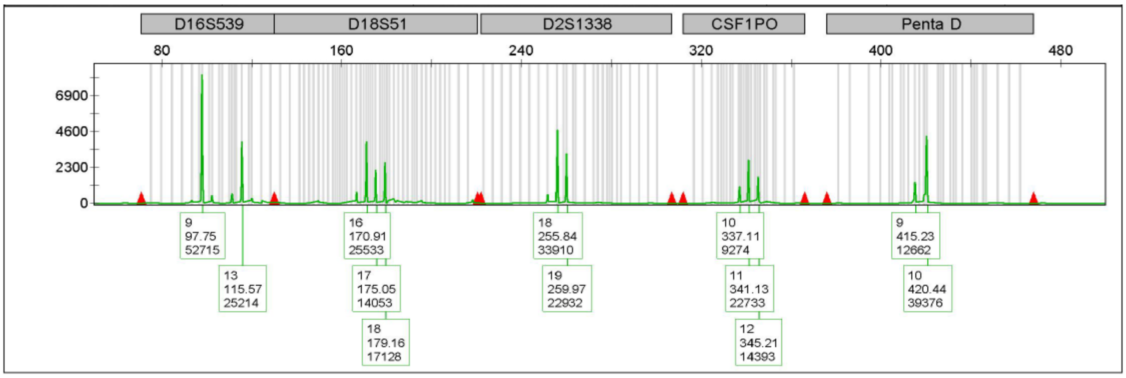

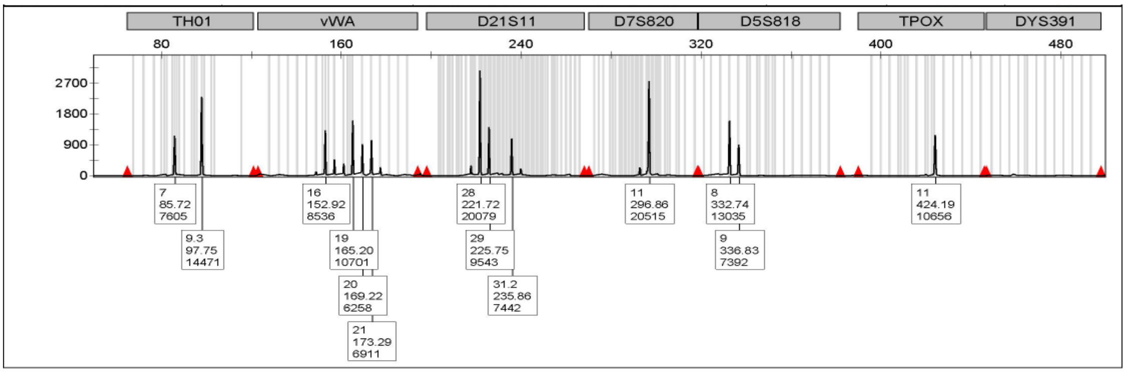

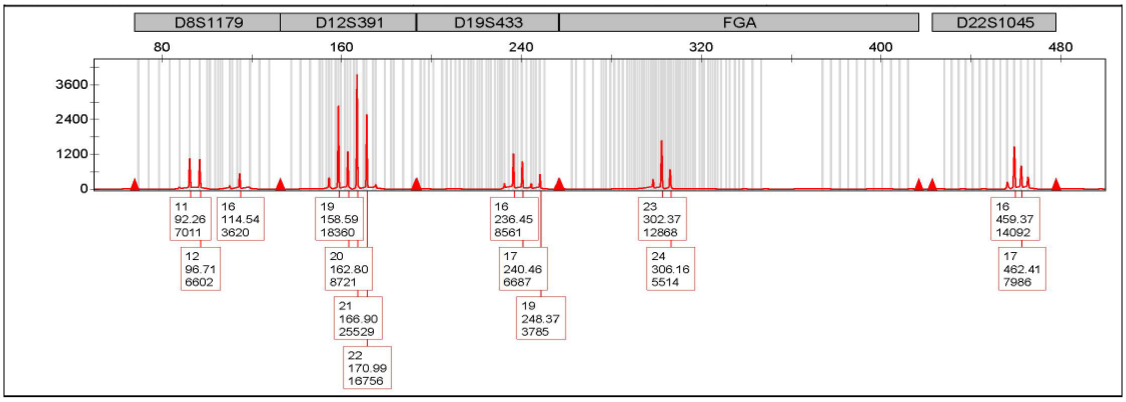

	Dye	Sample File Name	Marker	Allele	Size	Height	Area
1	B,1	033_H10_HEK293T.fsa	AMEL	X	82.42	8164	58296
2	B,2	033_H10_HEK293T.fsa	D3S1358	15	124.38	5616	37890
3	B,3	033_H10_HEK293T.fsa	D3S1358	16	128.64	2089	14012
4	B,4	033_H10_HEK293T.fsa	D3S1358	17	132.87	3590	24189
5	B,5	033_H10_HEK293T.fsa	D1S1656	14	180.46	4988	34035
6	B,6	033_H10_HEK293T.fsa	D1S1656	15	184.48	3201	21740
7	B,7	033_H10_HEK293T.fsa	D1S1656	16	188.49	4329	29598
8	B,8	033_H10_HEK293T.fsa	D1S1656	17	192.48	1735	11754
9	B,9	033_H10_HEK293T.fsa	D1S1656	17.3	195.49	5913	40657
10	B,10	033_H10_HEK293T.fsa	D2S441	11	224.11	2139	15736
11	B,11	033_H10_HEK293T.fsa	D2S441	15	240.68	4260	31452
12	B,12	033_H10_HEK293T.fsa	D10S1248	13	272.23	1086	8649
13	B,13	033_H10_HEK293T.fsa	D10S1248	14	276.16	3540	27922
14	B,14	033_H10_HEK293T.fsa	D13S317	12	328.44	5055	42190
15	B,15	033_H10_HEK293T.fsa	D13S317	13	332.46	1480	12507
16	B,16	033_H10_HEK293T.fsa	Penta E	7	378.46	2739	25061
17	B,17	033_H10_HEK293T.fsa	Penta E	15	416.99	1952	18508
18	G,1	033_H10_HEK293T.fsa	D16S539	9	97.75	8282	52715
19	G,2	033_H10_HEK293T.fsa	D16S539	13	115.57	4001	25214
20	G,3	033_H10_HEK293T.fsa	D18S51	16	170.91	4009	25533
21	G,4	033_H10_HEK293T.fsa	D18S51	17	175.05	2167	14053
22	G,5	033_H10_HEK293T.fsa	D18S51	18	179.16	2658	17128
23	G,6	033_H10_HEK293T.fsa	D2S1338	18	255.84	4705	33910
24	G,7	033_H10_HEK293T.fsa	D2S1338	19	259.97	3214	22932
25	G,8	033_H10_HEK293T.fsa	CSF1PO	10	337.11	1137	9274
26	G,9	033_H10_HEK293T.fsa	CSF1PO	11	341.13	2792	22733
27	G,10	033_H10_HEK293T.fsa	CSF1PO	12	345.21	1737	14393
28	G,11	033_H10_HEK293T.fsa	Penta D	9	415.23	1435	12662
29	G,12	033_H10_HEK293T.fsa	Penta D	10	420.44	4356	39376
30	Y,1	033_H10_HEK293T.fsa	TH01	7	85.72	1182	7605
31	Y,2	033_H10_HEK293T.fsa	TH01	9.3	97.75	2306	14471
32	Y,3	033_H10_HEK293T.fsa	vWA	16	152.92	1339	8536
33	Y,4	033_H10_HEK293T.fsa	vWA	19	165.2	1610	10701
34	Y,5	033_H10_HEK293T.fsa	vWA	20	169.22	929	6258
35	Y,6	033_H10_HEK293T.fsa	vWA	21	173.29	1055	6911
36	Y,7	033_H10_HEK293T.fsa	D21S11	28	221.72	3075	20079

37	Y,8	033_H10_HEK293T.fsa	D21S11	29	225.75	1431	9543
38	Y,9	033_H10_HEK293T.fsa	D21S11	31.2	235.86	1092	7442
39	Y,10	033_H10_HEK293T.fsa	D7S820	11	296.86	2775	20515
40	Y,11	033_H10_HEK293T.fsa	D5S818	8	332.74	1622	13035
41	Y,12	033_H10_HEK293T.fsa	D5S818	9	336.83	915	7392
42	Y,13	033_H10_HEK293T.fsa	TPOX	11	424.19	1195	10656
43	R,1	033_H10_HEK293T.fsa	D8S1179	11	92.26	1074	7011
44	R,2	033_H10_HEK293T.fsa	D8S1179	12	96.71	1041	6602
45	R,3	033_H10_HEK293T.fsa	D8S1179	16	114.54	564	3620
46	R,4	033_H10_HEK293T.fsa	D12S391	19	158.59	2892	18360
47	R,5	033_H10_HEK293T.fsa	D12S391	20	162.8	1302	8721
48	R,6	033_H10_HEK293T.fsa	D12S391	21	166.9	3962	25529
49	R,7	033_H10_HEK293T.fsa	D12S391	22	170.99	2591	16756
50	R,8	033_H10_HEK293T.fsa	D19S433	16	236.45	1231	8561
51	R,9	033_H10_HEK293T.fsa	D19S433	17	240.46	964	6687
52	R,10	033_H10_HEK293T.fsa	D19S433	19	248.37	542	3785
53	R,11	033_H10_HEK293T.fsa	FGA	23	302.37	1696	12868
54	R,12	033_H10_HEK293T.fsa	FGA	24	306.16	714	5514
55	R,13	033_H10_HEK293T.fsa	D22S1045	16	459.37	1461	14092
56	R,14	033_H10_HEK293T.fsa	D22S1045	17	462.41	810	7986

The comparison between case result and STR profile database:

Cell Name	Locus name / STR Repeat Number									Match (%)	STR Profile Database*
	D5S818	D13S317	D7S820	D16S539	VWA	TH01	AM	TPOX	CSF1PO		
Test Sample	8,9	12,13	11,11	9,13	16,19, 20,21	7,9.3	X,X	11,11	10,11,12		
Phoenix-Eco	8,9	11,12, 13	11,11	9,13	16,19	7,9.3	X,X	11,11	11,12	87.5	ExPASy
HEK293T	8,9	12,12	11,11	9,13	16,19	7,9.3	X,X	11,11	11,12	86.67	ExPASy
OAT1 HEK 293T/17	8,9	12,13, 14	11,11	9,13	16,18, 19	7,9.3	X,X	11,11	11,12	84.85	ExPASy
293 GTP-AC-free	8,9	12,12	11,12	9,13	16,19	7,9.3	X,X	11,11	11,12	83.87	ExPASy
293T GNE	8,9	12,12	11,12	9,13	16,19	7,9.3	X,X	11,11	11,12	83.87	ExPASy
AAV-293	8,9	12,12	11,11	9,13	16,19	7,9.3	X,X	11,11	7,11,12	83.87	ExPASy
HEK293	8,9	12,12	11,12	9,13	16,19	7,9.3	X,X	11,11	11,12	83.87	ExPASy
HEK293T-GIGYF1/2-null	8,9	11,12	11,12	9,13	16,18, 19	7,9.3	X,X	11,11	11,12	83.87	ExPASy
Phoenix-gp	8,9	12,14	11,11	9,13	16,19	7,9.3	X,X	11,11	11,12	83.87	ExPASy
Tau RD P301S FRET Biosensor	8,9	12,14	11,11	9,13	16,19	7,9.3	X,X	11,11	11,12	83.87	ExPASy

* Online STR Analysis is performed on some bioresource websites as below:

https://web.expasy.org/cellosaurus-str-search/ (ExPASy)

https://www.atcc.org/STR_Database.aspx (ATCC)

https://www.dsmz.de/services/services-human-and-animal-cell-lines/online-str-analysis.html (DSMZ)

* This table just shows the top 10 cell lines within database those matches are ≥80%, and if there isn't any match ≥80%, it just shows the cell line which scores highest match with the test sample. If necessary, please utilize the online STR analysis on ATCC/DSMZ website for detailed comparison.

The interpretation of comparison:

Depending on the comparison, the highest 87.5% match is between the test sample and Phoenix-Eco cell line within database, and there are other nine cell lines those matches are ≥80%.

Referring to ASN-0002-2011 (Authentication of Human Cell Lines: Standardization of STR Profiling), Cell line samples matching at ≥80% of alleles across the eight (8) core loci are said to be related, allowing authentication of the sample being tested. STR profiles are said to be unrelated when they match at <55% of alleles. STR profiles matching at 55-80% of alleles may be related and warrant further investigation as there may be overlap with a small number of cell lines that show marked

genetic instability and slip below the 80% threshold.

- ***This STR analysis service is for research purposes only, and any medical usage is not allowed.***

勁因科技有限公司

Genelabs Life science Corporation

www.genelabs.com.tw

Phone:(02)26557678, (04)22633813, (06)2094380

Freecall:0800-231914, 0800-094380

REPORT DOWNLOAD

Cell Line DNA Typing Report

Case Number: CID20220086

Report Date: 10/05/2022

Genelabs Life Science Corp.
12F.-6, No.3, Yuanqu St.,
Nangang Dist.,
Taipei City 115, Taiwan
TEL: +886-2-26557678
FAX: +886-2-26557572
E-mail: cellid@genelabs.com.tw

Sample Information:

i. Applicant Name: 王齡玉 Ling-Yu Wang

ii. Institution: 長庚大學生化科 Department of Biochemistry and Molecular Biology, Chang Gung University, Taoyuan, Taiwan

iii. Sample Description: DU145

iv. Sample type: Cell Pellet

v. Sample Received Date: 09/23/2022

Test Description:

DNA of the sample is extracted by Roche MagNA Pure Compact System.

DNA conc. = 90.1 ng/µl; OD260/280 = 1.97 ; OD260/230 = 1.95

The STR loci are amplified by Promega GenePrint® 24 System.

The CE analysis is performed on ABI PRISM 3730 GENETIC ANALYZER.

The raw data is analyzed by GeneMapper® Software V3.7.

The STR analysis is operated and reported by Mission Biotech.

This report is issued by:

Laboratory Director　　General Manager

STR Analysis Result:

**ANSI/ATCC ASN-0002 STR Loci**	**Repeat Numbers**	Extended STR Loci	Repeat Numbers	Extended STR Loci	Repeat Numbers
**D5S818**	10,13	D3S1358	15,16	DYS391*	10
**D13S317**	12,13,14	D1S1656	15,16,17.3	D8S1179	13,14
**D7S820**	7,10,11	D2S441	9,14	D12S391	18,21
**D16S539**	11,13	D10S1248	12,13	D19S433	12,13
**vWA**	17,18	Penta E	12,14	FGA	22,22
**TH01**	7,7	D18S51	12,13	D22S1045	16,16
**Amelogenin**	X,Y	D2S1338	16,16	Case Number: CID20220086<br>Test Date: 10/04/2022	
**TPOX**	11,11	Penta D	9,13		
**CSF1PO**	10,11	D21S11	30,33		

Allele Report

Case Number: CID20220086

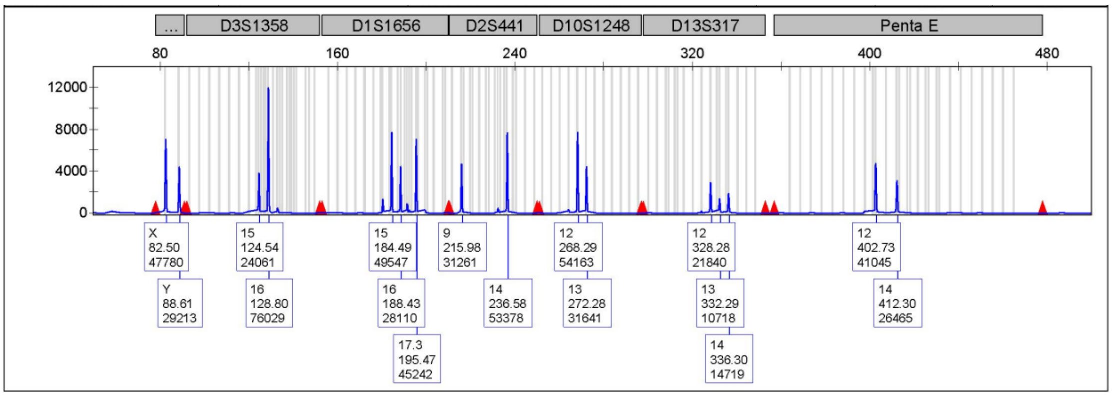

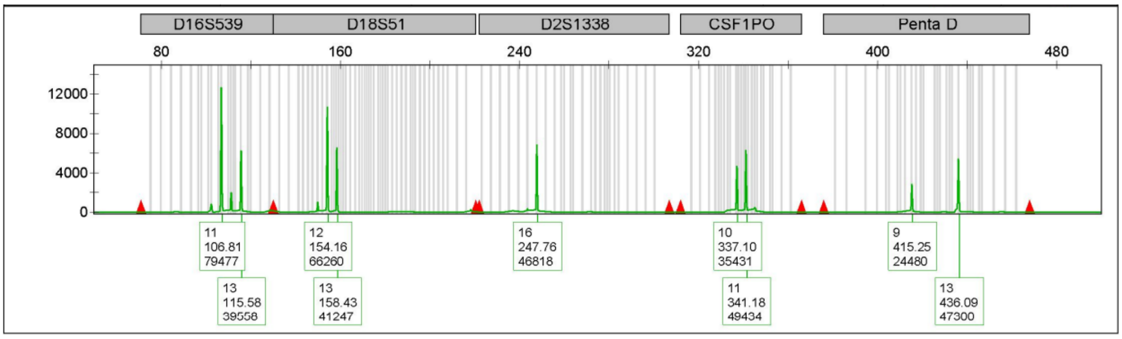

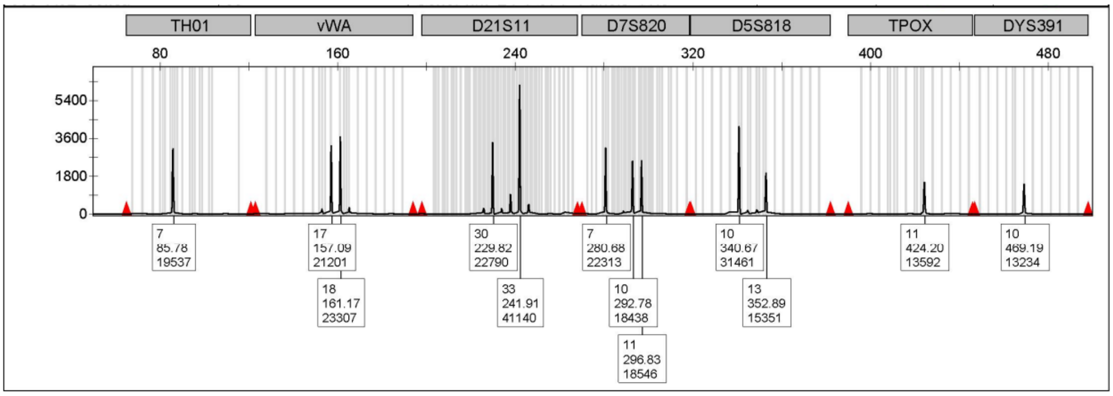

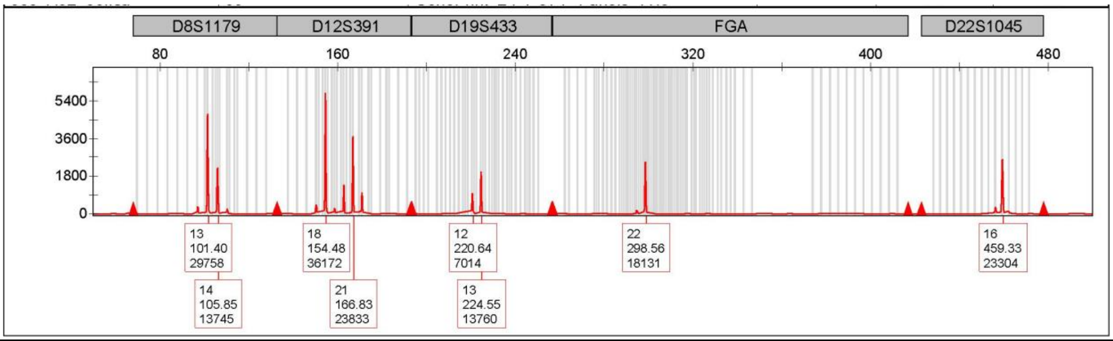

	Dye	Sample File Name	Marker	Allele	Size	Height	Area
1	B,1	008_A02_DU145.fsa	AMEL	X	82.5	7138	47780
2	B,2	008_A02_DU145.fsa	AMEL	Y	88.61	4412	29213
3	B,3	008_A02_DU145.fsa	D3S1358	15	124.54	3798	24061
4	B,4	008_A02_DU145.fsa	D3S1358	16	128.8	11978	76029
5	B,7	008_A02_DU145.fsa	D1S1656	15	184.49	7732	49547
6	B,8	008_A02_DU145.fsa	D1S1656	16	188.43	4444	28110
7	B,10	008_A02_DU145.fsa	D1S1656	17.3	195.47	7170	45242
8	B,11	008_A02_DU145.fsa	D2S441	9	215.98	4697	31261
9	B,13	008_A02_DU145.fsa	D2S441	14	236.58	7765	53378
10	B,15	008_A02_DU145.fsa	D10S1248	12	268.29	7825	54163
11	B,16	008_A02_DU145.fsa	D10S1248	13	272.28	4477	31641
12	B,17	008_A02_DU145.fsa	D13S317	12	328.28	2922	21840
13	B,18	008_A02_DU145.fsa	D13S317	13	332.29	1406	10718
14	B,19	008_A02_DU145.fsa	D13S317	14	336.3	1891	14719
15	B,20	008_A02_DU145.fsa	Penta E	12	402.73	4846	41045
16	B,21	008_A02_DU145.fsa	Penta E	14	412.3	3132	26465
17	G,2	008_A02_DU145.fsa	D16S539	11	106.81	12733	79477
18	G,4	008_A02_DU145.fsa	D16S539	13	115.58	6239	39558
19	G,6	008_A02_DU145.fsa	D18S51	12	154.16	10775	66260
20	G,7	008_A02_DU145.fsa	D18S51	13	158.43	6637	41247
21	G,9	008_A02_DU145.fsa	D2S1338	16	247.76	6930	46818
22	G,10	008_A02_DU145.fsa	CSF1PO	10	337.1	4728	35431
23	G,11	008_A02_DU145.fsa	CSF1PO	11	341.18	6387	49434
24	G,13	008_A02_DU145.fsa	Penta D	9	415.25	2882	24480
25	G,14	008_A02_DU145.fsa	Penta D	13	436.09	5440	47300
26	Y,1	008_A02_DU145.fsa	TH01	7	85.78	3150	19537
27	Y,2	008_A02_DU145.fsa	vWA	17	157.09	3295	21201
28	Y,3	008_A02_DU145.fsa	vWA	18	161.17	3707	23307
29	Y,4	008_A02_DU145.fsa	D21S11	30	229.82	3432	22790
30	Y,6	008_A02_DU145.fsa	D21S11	33	241.91	6171	41140
31	Y,8	008_A02_DU145.fsa	D7S820	7	280.68	3176	22313
32	Y,9	008_A02_DU145.fsa	D7S820	10	292.78	2561	18438
33	Y,10	008_A02_DU145.fsa	D7S820	11	296.83	2570	18546
34	Y,11	008_A02_DU145.fsa	D5S818	10	340.67	4186	31461
35	Y,12	008_A02_DU145.fsa	D5S818	13	352.89	1989	15351
36	Y,13	008_A02_DU145.fsa	TPOX	11	424.2	1550	13592

37	Y,14	008_A02_DU145.fsa	DYS391	10	469.19	1426	13234
38	R,1	008_A02_DU145.fsa	D8S1179	13	101.4	4821	29758
39	R,2	008_A02_DU145.fsa	D8S1179	14	105.85	2225	13745
40	R,3	008_A02_DU145.fsa	D12S391	18	154.48	5816	36172
41	R,5	008_A02_DU145.fsa	D12S391	21	166.83	3751	23833
42	R,7	008_A02_DU145.fsa	D19S433	12	220.64	1053	7014
43	R,8	008_A02_DU145.fsa	D19S433	13	224.55	2030	13760
44	R,9	008_A02_DU145.fsa	FGA	22	298.56	2509	18131
45	R,10	008_A02_DU145.fsa	D22S1045	16	459.33	2619	23304

The comparison between case result and STR profile database:

Cell Name	Locus name / STR Repeat Number									Match (%)	STR Profile Database*
	D5S818	D13S317	D7S820	D16S539	VWA	TH01	AM	TPOX	CSF1PO		
Test Sample	10,13	12,13,14	7,10,11	11,13	15,15	7,7	X,Y	11,11	10,11		
DU145	10,13	12,13,14	7,10,11	11,13	16,17,18	7,7	X,Y	11,11	10,11	87.5	ExPASy
DU145-Cas9-540	10,13	12,14	7,10,11	11,11	17,18	7,7	X,Y	11,11	10,11	82.76	ExPASy
DU145-Cas9-541	10,13	12,14	7,10,11	11,11	17,18	7,7	X,Y	11,11	10,11	82.76	ExPASy
DU-145-Luc	10,13	12,13	7,10,10.3,11	11,12,13	17,18	7,7	X,Y	11,11	10,11	81.25	ExPASy
DU145	10,13	12,14	7,10,11	11,12,13	16,17,18	7,7	X,Y	11,11	10,11	81.25	ExPASy

* Online STR Analysis is performed on some bioresource websites as below:

https://web.expasy.org/cellosaurus-str-search/ (ExPASy)

https://www.atcc.org/STR_Database.aspx (ATCC)

https://www.dsmz.de/services/services-human-and-animal-cell-lines/online-str-analysis.html (DSMZ)

* This table just shows the top 10 cell lines within database those matches are ≥80%, and if there isn't any match ≥80%, it just shows the cell line which scores highest match with the test sample. If necessary, please utilize the online STR analysis on ATCC/DSMZ website for detailed comparison.

The interpretation of comparison:

Depending on the comparison, the highest 87.5% match is between the test sample and DU145 cell line within database, and there are other four cell lines those matches are ≥80%.

Referring to ASN-0002-2011 (Authentication of Human Cell Lines: Standardization of STR Profiling), Cell line samples matching at ≥80% of alleles across the eight (8) core loci are said to be related, allowing authentication of the sample being tested. STR profiles are said to be unrelated when they match at <55% of alleles. STR profiles matching at 55-80% of alleles may be related and warrant further investigation as there may be overlap with a small number of cell lines that show marked genetic instability and slip below the 80% threshold.

- ***This STR analysis service is for research purposes only, and any medical usage is not allowed.***

GeneLabs Life Science Innovation
勁因科技有限公司

Genelabs Life science Corporation

www.genelabs.com.tw

Phone:(02)26557678, (04)22633813, (06)2094380

Freecall:0800-231914, 0800-094380

REPORT DOWNLOAD

Cell Line DNA Typing Report

Case Number: CID20220099

Report Date: 12/23/2022

Genelabs Life Science Corp.
12F.-6, No.3, Yuanqu St.,
Nangang Dist.,
Taipei City 115, Taiwan
TEL: +886-2-26557678
FAX: +886-2-26557572
E-mail: cellid@genelabs.com.tw

Sample Information:

i. Applicant Name: 王齡玉 Ling-Yu Wang

ii. Institution: 長庚大學生化科 Department of Biochemistry and Molecular Biology, Chang Gung University, Taoyuan, Taiwan

iii. Sample Description: PC3

iv. Sample type: Cell Pellet

v. Sample Received Date:12/16/2022

Test Description:

DNA of the sample is extracted by Roche MagNA Pure Compact System.

DNA conc. = 69.5 ng/µl; OD260/280 = 1.99 ; OD260/230 = 1.97

The STR loci are amplified by Promega GenePrint® 24 System.

The CE analysis is performed on ABI PRISM 3730 GENETIC ANALYZER.

The raw data is analyzed by GeneMapper® Software V3.7.

The STR analysis is operated and reported by Mission Biotech.

This report is issued by:

Liang Kuei Chang James Chung

Laboratory Director General Manager

STR Analysis Result:

ANSI/ATCC ASN-0002 STR Loci	Repeat Numbers	Extended STR Loci	Repeat Numbers	Extended STR Loci	Repeat Numbers
**D5S818**	13,13	D3S1358	16,16	DYS391*	N/A
**D13S317**	11,11	D1S1656	12,16	D8S1179	13,13
**D7S820**	8,11	D2S441	10,11	D12S391	21,21
**D16S539**	11,11	D10S1248	16,16	D19S433	14,14
**vWA**	17,17	Penta E	10,17	FGA	24,24
**TH01**	6,7	D18S51	14,15	D22S1045	15,15
**Amelogenin**	X,X	D2S1338	18,20	Case Number: CID20220099<br>Test Date: 12/19/2022	
**TPOX**	8,9	Penta D	9,9		
**CSF1PO**	11,11	D21S11	29,31.2		

Allele Report

Case Number: CID20220099

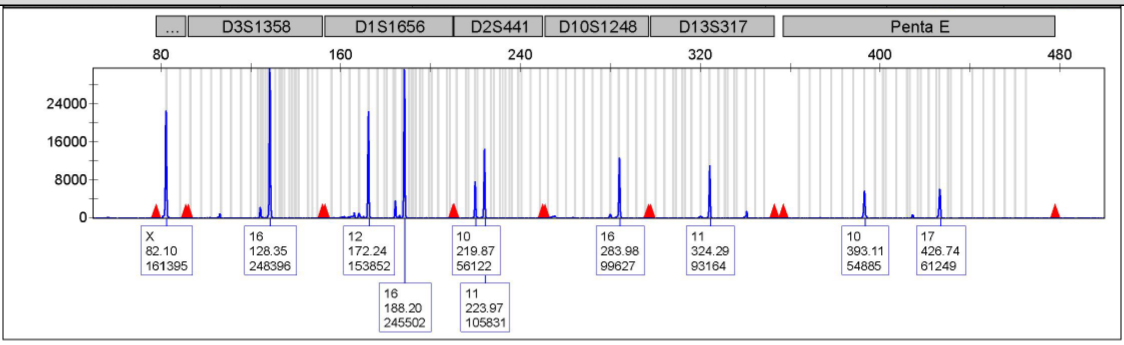

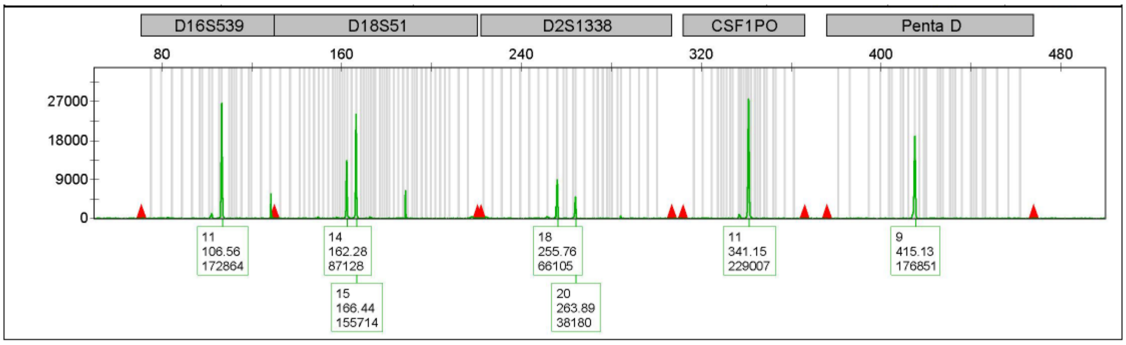

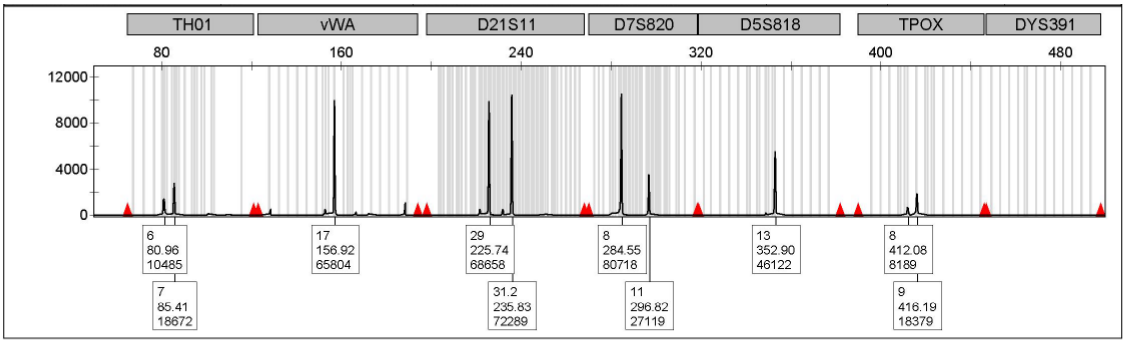

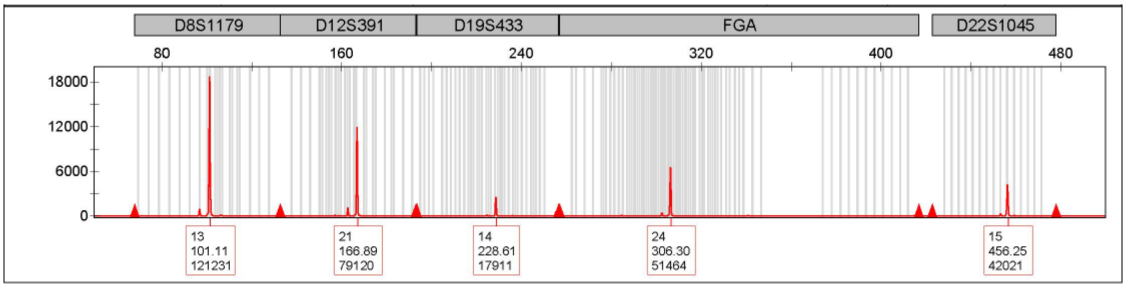

	Dye	Sample File Name	Marker	Allele	Size	Height	Area
1	B,1	024_A05_PC3.fsa	AMEL	X	82.1	22678	161395
2	B,2	024_A05_PC3.fsa	D3S1358	16	128.35	31633	248396
3	B,3	024_A05_PC3.fsa	D1S1656	12	172.24	22529	153852
4	B,4	024_A05_PC3.fsa	D1S1656	16	188.2	31441	245502
5	B,5	024_A05_PC3.fsa	D2S441	10	219.87	7773	56122
6	B,6	024_A05_PC3.fsa	D2S441	11	223.97	14612	105831
7	B,7	024_A05_PC3.fsa	D10S1248	16	283.98	12707	99627
8	B,8	024_A05_PC3.fsa	D13S317	11	324.29	11109	93164
9	B,9	024_A05_PC3.fsa	Penta E	10	393.11	5870	54885
10	B,10	024_A05_PC3.fsa	Penta E	17	426.74	6304	61249
11	G,1	024_A05_PC3.fsa	D16S539	11	106.56	26819	172864
12	G,2	024_A05_PC3.fsa	D18S51	14	162.28	13549	87128
13	G,3	024_A05_PC3.fsa	D18S51	15	166.44	24206	155714
14	G,4	024_A05_PC3.fsa	D2S1338	18	255.76	9110	66105
15	G,5	024_A05_PC3.fsa	D2S1338	20	263.89	5302	38180
16	G,6	024_A05_PC3.fsa	CSF1PO	11	341.15	27740	229007
17	G,7	024_A05_PC3.fsa	Penta D	9	415.13	19271	176851
18	Y,1	024_A05_PC3.fsa	TH01	6	80.96	1557	10485
19	Y,2	024_A05_PC3.fsa	TH01	7	85.41	2861	18672
20	Y,3	024_A05_PC3.fsa	vWA	17	156.92	10046	65804
21	Y,4	024_A05_PC3.fsa	D21S11	29	225.74	9955	68658
22	Y,5	024_A05_PC3.fsa	D21S11	31.2	235.83	10548	72289
23	Y,6	024_A05_PC3.fsa	D7S820	8	284.55	10622	80718
24	Y,7	024_A05_PC3.fsa	D7S820	11	296.82	3583	27119
25	Y,8	024_A05_PC3.fsa	D5S818	13	352.9	5585	46122
26	Y,9	024_A05_PC3.fsa	TPOX	8	412.08	891	8189
27	Y,10	024_A05_PC3.fsa	TPOX	9	416.19	1935	18379
28	R,1	024_A05_PC3.fsa	D8S1179	13	101.11	18777	121231
29	R,2	024_A05_PC3.fsa	D12S391	21	166.89	12013	79120
30	R,3	024_A05_PC3.fsa	D19S433	14	228.61	2584	17911
31	R,4	024_A05_PC3.fsa	FGA	24	306.3	6533	51464
32	R,5	024_A05_PC3.fsa	D22S1045	15	456.25	4299	42021

The comparison between case result and STR profile database:

Cell Name	Locus name / STR Repeat Number									Match (%)	STR Profile Database*
	D5S818	D13S317	D7S820	D16S539	VWA	TH01	AM	TPOX	CSF1PO		
Test Sample	13,13	11,11	8,11	11,11	17,17	6,7	X,X	8,9	11,11		
JHU-019	13,13	11,11	8,11	11,11	17,17	6,7	X,X	8,9	11,11	100	ExPASy
PC-3	13,13	11,11	8,11	11,11	17,17	6,7	X,X	8,9	11,11	100	ExPASy
PC-3-Cas9-576	13,13	11,11	8,11	11,11	17,17	6,7	X,X	8,9	11,11	100	ExPASy
PC-3-Cas9-577	13,13	11,11	8,11	11,11	17,17	6,7	X,X	8,9	11,11	100	ExPASy
PC-3-Cas9-578	13,13	11,11	8,11	11,11	17,17	6,7	X,X	8,9	11,11	100	ExPASy
PC-3-Cas9-579	13,13	11,11	8,11	11,11	17,17	6,7	X,X	8,9	11,11	100	ExPASy
PC-3-Luc	13,13	11,11	8,11	11,11	17,17	6,7	X,X	8,9	11,11	100	ExPASy
PC-3-Luc2	13,13	11,11	8,11	11,11	17,17	6,7	X,X	8,9	11,11	100	ExPASy
PPC-1	13,13	11,11	8,11	11,11	17,17	6,7	X,X	8,9	11,11	100	ExPASy
JHU-019	9,13	11,11	8,11	11,11	17,17	6,7	X,X	8,9	11,11	95.65	ExPASy

* Online STR Analysis is performed on some bioresource websites as below:

https://web.expasy.org/cellosaurus-str-search/ (ExPASy)

https://www.atcc.org/STR_Database.aspx (ATCC)

https://www.dsmz.de/services/services-human-and-animal-cell-lines/online-str-analysis.html (DSMZ)

* This table just shows the top 10 cell lines within database those matches are ≥80%, and if there isn't any match ≥80%, it just shows the cell line which scores highest match with the test sample. If necessary, please utilize the online STR analysis on ATCC/DSMZ website for detailed comparison.

The interpretation of comparison:

Depending on the comparison, the highest 100% match is between the test sample and nine cell lines (JHU-019, PC-3, PC-3-Cas9-576, PC-3-Cas9-577, PC-3-Cas9-578, PC-3-Cas9-579, PC-3-Luc, PC-3-Luc2, PPC-1) within database, and there is another cell line that match is ≥80%.

Referring to ASN-0002-2011 (Authentication of Human Cell Lines: Standardization of STR Profiling), Cell line samples matching at ≥80% of alleles across the eight (8) core loci are said to be related, allowing authentication of the sample being tested. STR profiles are said to be unrelated when they match at <55% of alleles. STR profiles matching at 55-80% of alleles may be related and warrant further investigation as there may be overlap with a small number of cell lines that show marked genetic instability and slip below the 80% threshold.

- ***This STR analysis service is for research purposes only, and any medical usage is not allowed.***

REPORT DOWNLOAD

勁因科技有限公司

Genelabs Life science Corporation

www.genelabs.com.tw

Phone:(02)26557678, (04)22633813, (06)2094380

Freecall:0800-231914, 0800-094380

Anti-Androgen Receptor

4°C

Polyclonal Antibody
Cat. # 06-680
Lot # 3324594

pack size: 100 µL
Store at 2-8°C

FOR RESEARCH USE ONLY
NOT FOR USE IN DIAGNOSTIC PROCEDURES
NOT FOR HUMAN OR ANIMAL CONSUMPTION

Certificate of Analysis

Applications	Species Cross-Reactivity	Antibody Isotype	Epitope/ Region	Host Species	Molecular Weight	Accession #
WB, IP, FC, ChIP, IHC, ChIP Seq	H, R, (M, B, Eq, Ca)	N/A	N-terminus	Rb	~110 kDa	NP_036634

Background

The androgen receptor (AR) is a type of nuclear receptor which is activated by binding of either of the androgenic hormones testosterone or dihydrotestosterone. The main function of the androgen receptor is as a DNA binding transcription factor which regulates gene expression. However the androgen receptor also has additional functions independent of DNA binding. The androgen receptor is most closely related to the progesterone receptor, and progestins in higher dosages can block the androgen receptor.

Presentation

Purified rabbit polyclonal in buffer containing 0.1 M Tris-Glycine (pH 7.4), 0.15M NaCl with 0.05% sodium azide.

Species Cross-reactivity

Demonstrated to react with Human and Rat. Predicted to react with Mouse, Bovine, Equine, and Canine based on 100% sequence homology.

Immunogen

KLH-conjugated linear peptide corresponding to the N-terminus of rat Androgen Receptor.

Molecular Weight

~110 kDa observed

Method of Purification

Affinity purified

Storage and Handling

Stable for 1 year at 2-8°C from date of receipt.

Quality Control Testing

Evaluated by Western Blotting in LNCaP cell lysate.

Western Blotting Analysis: A 1:1,000 dilution of this antibody detected Androgen Receptor in 10 µg of LNCaP cell lysate.

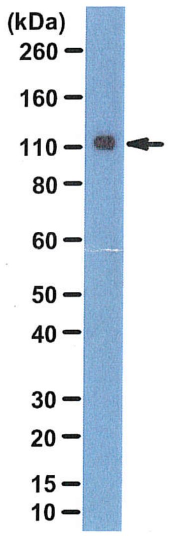

Western Blotting Analysis: Representative lot data. LNCaP cell lysate was probed with Cat. No. 06-680, Anti-Androgen Receptor (1:1,000 dilution). Proteins were visualized using a Goat Anti-Rabbit IgG secondary antibody conjugated to HRP and a chemiluminescence detection system.

Arrow indicates Androgen Receptor (~110 kDa).

Additional Research Applications

Chromatin Immunoprecipitation Analysis: A representative lot from an independent laboratory immunoprecipitated Androgen Receptor from DU145 cells stably transfected with a 7 kD-PSA promoter, different AR poly A lentiviral vectors, and a Flag-tagged hZMIZ1 vector (Li, X., *et al.* (2011). *PLoS One.* 6(9):e25040.).

References

Product Reference(s):
1. Li, X., *et al.* (2011). *PLoS One.* 6(9):e25040.
2. Nation, T., *et al.* (2010). *J Pediatr Surg.* 46(8): 1539-1543.
3. Ghatge, R. P., *et al.* (2005). *Breast Cancer Res.* 7(6):R1036-1050.
4. Zhao, J. C., *et al.* (2012). *Genome Res.* 22(2):322-331.

Immunohistochemistry Analysis: A representative lot from an independent laboratory detected Androgen Receptor in embryonic rat tissue, neonatal rat testis tissue, and adult rat testis tissue (Nation, T., *et al.* (2010). *J Pediatr Surg.* 46(8): 1539-1543.).

Immunocytochemistry Analysis: A representative lot from an independent laboratory detected Androgen Receptor in MCF-7 cells stably transfected with Androgen Receptor (Ghatge, R. P., *et al.* (2005). *Breast Cancer Res.* 7(6):R1036-1050.).

Chromatin Immunoprecipitaton-Sequence Analysis: A representative lot from an independent laboratory immunoprecipitated Androgen Receptor from LNCaP cell lysates under certain treated conditions (Zhao, J. C., *et al.* (2012). *Genome Res.* 22(2):322-331.).

APPLICATION LEGEND: WB Western Blotting **IP** Immunoprecipitation **ICC** Immunocytochemistry **FC** Flow Cytometry **IF** Immunofluorescence **IHC** Immunohistochemistry (Tissue) **IHC(P)** Immunohistochemistry (Paraffin) **ChIP SEQ** Chromatin Immunoiprecipitation-Sequence
SPECIES LEGEND: H Human **M** Mouse **R** Rat **Rb** Rabbit **B** Bovine **Eq** Equine **Ca** Canine **WR** Most Common Vertebrates **( )** Predicted Reactivity
Please visit www.millipore.com for additional product information, test data and references.

Submit your published journal article, and earn credit toward future Millipore purchases. Visit www.millipore.com/publicationrewards to learn more!

EMD Millipore Corporation, 28820 Single Oak Drive, Temecula, CA 92590, USA 1-800-437-7500
Technical Support: T: 1-800-MILLIPORE (1-800-645-5476) • F: 1-800-437-7502

FOR RESEARCH USE ONLY. Not for use in diagnostic procedures. Not for human or animal consumption. Purchase of this Product does not include any right to resell or transfer, either as a stand-alone product or as a component of another product. Any use of this Product for purposes other than research is strictly prohibited.Millipore, the M mark, Upstate, Chemicon, Linco and all other trademarks, unless specifically identified above in the text as belonging to a third party, are owned by Merck KGaA, Darmstadt. Copyright ©2008-2013 Merck KGaA, Darmstadt. All rights reserved.

Ver 8.0/2016-09-15/06-680CA/MS

Additional Research Applications

Immunoprecipitation Analysis: 34 µL from a representative lot immunoprecipitated Androgen Receptor from 1000 µg of LNCaP cell lysate.

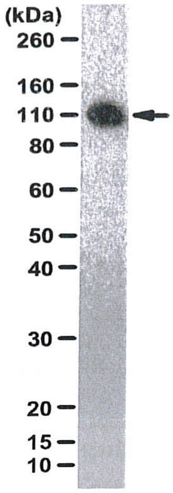

Immunoprecipitation Analysis:
Representative lot data.
34 µL of Cat. No. 06-680, Anti-Androgen Receptor immunoprecipitated Androgen Receptor from 1000 µg of LNCaP cell lysate. Immunoprecipitated sample was then probed using a 1:300 dilution of Cat. No. 06-680, Anti-Androgen Receptor . Proteins were visualized using an Anti-Rabbit secondary antibody conjugated to HRP and a chemiluminescence detection system.

Arrow indicates Androgen Receptor (~110 kDa).

Flow Cytometry Analysis: A 1:300 dilution from a representative lot detected Androgen Receptor in 0.2X10E6 MCF-7 cells.

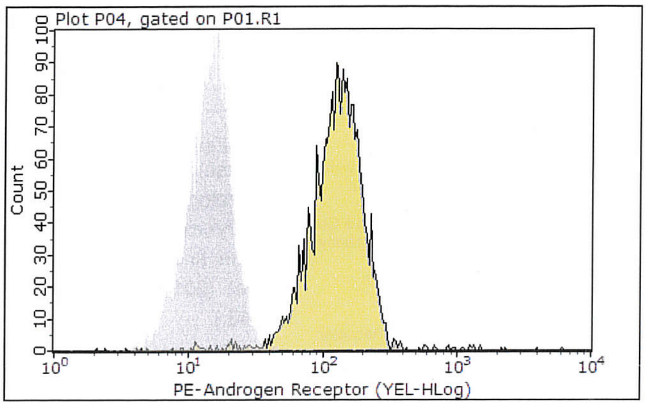

Flow Cytometry Analysis:
Representative lot data.
0.2X10E6 MCF-7 cells were stained with a 1:300 dilution of Cat. No. 06-680, Anti-Androgen Receptor (Yellow histogram), or with 1 µg/mL of Rabbit IgG isotype control (Grey histogram).

Green-e.org We Buy 100% Certified Renewable Energy

FOR RESEARCH USE ONLY. Not for use in diagnostic procedures. Not for human or animal consumption. Purchase of this Product does not include any right to resell or transfer, either as a stand-alone product or as a component of another product. Any use of this Product for purposes other than research is strictly prohibited.

Millipore, the M mark, Upstate, Chemicon, Linco and all other trademarks, unless specifically identified above in the text as belonging to a third party, are owned by Merck KGaA, Darmstadt. Copyright ©2008-2020 Merck KGaA, Darmstadt. All rights reserved.

RevMAb Biosciences
www.revmab.com

RevMAb Biosciences USA, Inc.
830 Dubuque Ave, South San Francisco, CA 94080, USA

Certificate of Analysis

**Product:**	Rabbit Monoclonal Antibody<br>Anti-Androgen Receptor (AR-V7 specific) Rabbit Monoclonal Antibody, Clone RM7
**Catalog No.:**	31-1109-00
**Lot No:**	
**Clone:**	RM7
**Specificity:**	This antibody reacts to androgen receptor splice variant 7 (AR-V7). No cross reactivity with wild type androgen receptor.
**Application:**	Immunohistochemistry, Immunocytochemistry, Immunoprecipitation, Western Blot, Chromatin IP, CTCs, Flow Cytometry
**Immunogen:**	A peptide corresponding to AR splice variant 7
**Purity:**	Protein A affinity purified from an animal origin–free culture supernatant.
**Size:**	100 µL
**Buffer:**	50% Glycerol/PBS with 1% BSA and 0.09% sodium azide
**Usage:**	IHC: 1:500 dilution;<br>ICC: 1:500 dilution;<br>WB: 1:1000 dilution.
**Storage and Stability:**	Stable for 1 Year at -20.0°C from date of receipt.
**Country of Origin:**	U.S.A.
**Intended Use:**	**For Research Use Only Not for Diagnostic or Therapeutic Use**

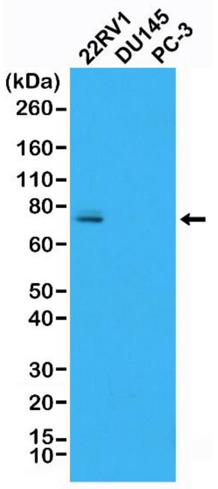

Western Blot analysis of 22RV1, DU145, and PC-3 whole cell lysates, using anti-AR-V7 rabbit monoclonal Antibody Clone RM7, showed endogenous AR-V7 expressed in 22RV1 cells.

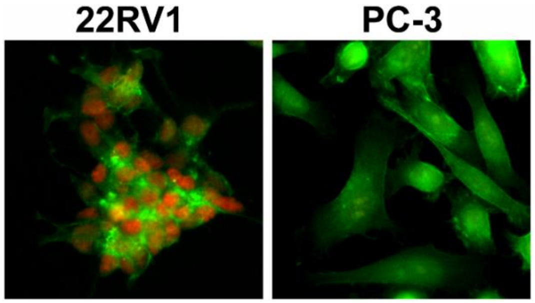

Immunocytochemical staining of 22RV1 and PC-3 cells, using anti-AR-V7 rabbit monoclonal Antibody Clone RM7 (red). Actin filaments have been labeled with fluorescein phalloidin (green).

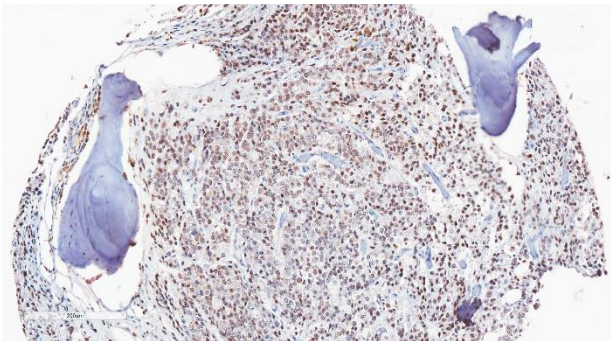

Immunohistochemical staining of formalin fixed and paraffin metastasis prostate cancer. Clinically validated to be positive for ARv7. (Image is provided by Dr. Stephen Plymate, University of Washington, USA)

#2118 Store at -20C

GAPDH (14C10) Rabbit mAb

Cell Signaling TECHNOLOGY®

Orders: 877-616-CELL (2355) orders@cellsignal.com

Support: 877-678-TECH (8324)

Web: info@cellsignal.com www.cellsignal.com

3 Trask Lane | Danvers | Massachusetts | 01923 | USA

For Research Use Only. Not For Use In Diagnostic Procedures.

Applications:	Reactivity:	Sensitivity:	MW (kDa):	Source/Isotype:	UniProt ID:	Entrez-Gene Id:
WB, IHC-P, IF-IC, FC-FP	H M R Mk B Pg	Endogenous	37	Rabbit	P04406	2597

Product Usage Information

Application	Dilution
Western Blotting	1:1000
Immunohistochemistry (Paraffin)	1:400 - 1:1600
Immunofluorescence (Immunocytochemistry)	1:50 - 1:200
Flow Cytometry (Fixed/Permeabilized)	1:100 - 1:400

Storage

Supplied in 10 mM sodium HEPES (pH 7.5), 150 mM NaCl, 100 µg/ml BSA, 50% glycerol and less than 0.02% sodium azide. Store at –20°C. Do not aliquot the antibody.

Specificity / Sensitivity

GAPDH (14C10) Rabbit mAb detects endogenous levels of total GAPDH protein.

Species Reactivity:
Human, Mouse, Rat, Monkey, Bovine, Pig

Species predicted to react based on 100% sequence homology:
Pig

Source / Purification

Monoclonal antibody is produced by immunizing animals with a synthetic peptide near the carboxy terminus of human GAPDH.

Background

Glyceraldehyde-3-phosphate dehydrogenase (GAPDH) catalyzes the phosphorylation of glyceraldehyde-3-phosphate during glycolysis. Though differentially expressed from tissue to tissue (1), GAPDH is thought to be a constitutively expressed housekeeping protein. For this reason, GAPDH mRNA and protein levels are often measured as controls in experiments quantifying specific changes in expression of other targets. Recent work has elucidated roles for GAPDH in apoptosis (2), gene expression (3), and nuclear transport (4). GAPDH may also play a role in neurodegenerative pathologies such as Huntington and Alzheimer's diseases (4,5).

1. Barber, R.D. et al. (2005) *Physiol. Genomics* 21, 389-95.
2. Hara, M.R. and Snyder, S.H. (2006) *Cell Mol. Neurobiol.* 26, 527-38.
3. Zheng, L. et al. (2003) *Cell* 114, 255-66.
4. Bae, B.I. et al. (2006) *Proc. Natl. Acad. Sci. USA* 103, 3405-9.
5. Wang, Q. et al. (2005) *FASEB J.* 19, 869-71.

Species reactivity is determined by testing in at least one approved application (e.g., western blot).

IMPORTANT: For western blots, incubate membrane with diluted primary antibody in 5% w/v BSA, 1X TBS, 0.1% Tween® 20 at 4°C with gentle shaking, overnight.

APPLICATIONS KEY **WB:** Western Blotting **IHC-P:** Immunohistochemistry (Paraffin) **IF-IC:** Immunofluorescence (Immunocytochemistry) **FC-FP:** Flow Cytometry (Fixed/Permeabilized)

CROSS-REACTIVITY KEY **H:** human **M:** mouse **R:** rat **Hm:** hamster **Mk:** monkey **Vir:** virus **Mi:** mink **C:** chicken **Dm:** D. melanogaster **X:** Xenopus **Z:** zebrafish **B:** bovine **Dg:** dog **Pg:** pig **Sc:** S. cerevisiae **Ce:** C. elegans **Hr:** horse **Rab:** rabbit **All:** all species expected

Cell Signaling Technology is a trademark of Cell Signaling Technology, Inc.
Alexa Fluor is a registered trademark of Life Technologies Corporation.
All other trademarks are the property of their respective owners. Visit cellsignal.com/trademarks for more information.

#2118

GAPDH (14C10) Rabbit mAb

Limited Uses

Except as otherwise expressly agreed in a writing signed by a legally authorized representative of CST, the following terms apply to Products provided by CST, its affiliates or its distributors. Any Customer's terms and conditions that are in addition to, or different from, those contained herein, unless separately accepted in writing by a legally authorized representative of CST, are rejected and are of no force or effect.

Products are labeled with For Research Use Only or a similar labeling statement and have not been approved, cleared, or licensed by the FDA or other regulatory foreign or domestic entity, for any purpose. Customer shall not use any Product for any diagnostic or therapeutic purpose, or otherwise in any manner that conflicts with its labeling statement. Products sold or licensed by CST are provided for Customer as the end-user and solely for research and development uses. Any use of Product for diagnostic, prophylactic or therapeutic purposes, or any purchase of Product for resale (alone or as a component) or other commercial purpose, requires a separate license from CST. Customer shall (a) not sell, license, loan, donate or otherwise transfer or make available any Product to any third party, whether alone or in combination with other materials, or use the Products to manufacture any commercial products, (b) not copy, modify, reverse engineer, decompile, disassemble or otherwise attempt to discover the underlying structure or technology of the Products, or use the Products for the purpose of developing any products or services that would compete with CST products or services, (c) not alter or remove from the Products any trademarks, trade names, logos, patent or copyright notices or markings, (d) use the Products solely in accordance with CST Product Terms of Sale and any applicable documentation, and (e) comply with any license, terms of service or similar agreement with respect to any third party products or services used by Customer in connection with the Products.

3050 Spruce Street, St. Louis, MO 63103 USA
Tel: (800) 521-8956 (314) 771-5765 Fax: (800) 325-5052 (314) 771-5757
email: techservice@sial.com sigma-aldrich.com

Product Information

Monoclonal Anti-β-Actin
Clone AC-15
produced in mouse, ascites fluid

Catalog Number **A5441**

Product Description
Monoclonal Anti-β-Actin (mouse IgG1 isotype) is derived from the AC-15 hybridoma produced by the fusion of mouse myeloma cells and splenocytes from an immunized mouse. A slightly modified synthetic β-cytoplasmic actin N-terminal peptide Ac-Asp-Asp-Asp-Ile-Ala-Ala-Leu-Val-Ile-Asp-Asn-Gly-Ser-Gly-Lys conjugated to KLH was used as the immunogen.[1] The isotype is determined by a double diffusion immunoassay using Mouse Monoclonal Antibody Isotyping Reagents, Catalog Number ISO2.

Monoclonal Anti-β-Actin recognizes an epitope located on the N-terminal end of the β-isoform of actin. The antibody specifically labels β-actin in a wide variety of tissues and species using immunoblotting (42 kDa), immunofluorescent staining of cultured cell lines, and immunohistochemistry. In immunofluorescent staining of chicken gizzard ultrathin tissue cryosections, the antibody labels the dense bodies and the longitudinal channels linking consecutive dense bodies that are also occupied by desmin and the membrane-associated dense plaque.[1] It does not stain adult cardiac and skeletal muscles. The antibody cross reacts with β-actin expressing cells in human, bovine, sheep, pig, rabbit, cat, dog, mouse, rat, guinea pig, chicken, carp, and leech tissues, but not in amoeba nor *Drosophila*. It can be used for staining of acetone-fixed, frozen sections and EM preparations. The epitope recognized by the antibody is resistant to formalin-fixation and paraffin-embedding. Ethanol, B5, methacarn, or Bouin's solutions may also be used as fixatives.

The two major cytoskeletal proteins implicated in cell motility are actin and myosin. Actin and myosin are constituents of many cell types and are involved in a myriad of cellular processes including locomotion, secretion, cytoplasmic streaming, phagocytosis, and cytokinesis. Although actin is one of the most conserved eukaryotic proteins, it is expressed in mammals and birds as at least six isoforms characterized by electrophoresis and amino acid sequence analysis.[2,3] Four of them represent the differentiation markers of muscle tissues and two are found practically in all cells.

There are three α-actins (α-skeletal, α-cardiac, and α-smooth muscle), one β-actin (β-nonmuscle), and two γ-actins (γ-smooth muscle and γ-non-muscle). Actin isoforms show >90% overall sequence homology, but only 50–60% homology in their 18 NH_2-terminal residues.[4] The NH_2-terminal region of actin appears to be a major antigenic region and may be involved in the interaction of actin with other proteins such as myosin. The actin in cells of various species and tissue origin is very similar in its immunological and physical properties. Therefore, a specific antibody to β-actin provides a useful tool in studying the intracellular distribution of
β-actin and the static and dynamic aspects of the cytoskeleton.

Reagent
Supplied as ascites fluid with 15 mM sodium azide as a preservative.

Precautions and Disclaimer
This product is for R&D use only, not for drug, household, or other uses. Please consult the Material Safety Data Sheet for information regarding hazards and safe handling practices.

Storage

For continuous use, store at 2–8 °C for up to one month. For extended storage, the solution may be frozen in working aliquots. Repeated freezing and thawing, or storage in "frost-free" freezers, is not recommended. If slight turbidity occurs upon prolonged storage, clarify the solution by centrifugation before use.

Product Profile

Immunoblotting: a minimum dilution of 1:5,000 was determined using cultured human or chicken fibroblast cell extracts.

Indirect immunofluorescence: a minimum dilution of 1:1,000 was determined using cultured human or chicken fibroblasts.

Note: In order to obtain the best results, it is recommended that each individual user determine working dilution by titration.

References

1. North, A.J., et al., *J. Cell Biol.*, **120**, 1159-67 (1993).
2. Vandekerchove, J., and Weber, K., *Eur. J. Biochem.*, **90**, 451 (1978).
3. Drew, J., et al., *Amer. J. Physiol.*, **260**, C1332 (1991).
4. Lessard, J., *Cell Motil. Cytoskel.*, **10**, 349 (1988).

MG,KAA,PHC,MAM 10/08-1

Sigma brand products are sold through Sigma-Aldrich, Inc.
Sigma-Aldrich, Inc. warrants that its products conform to the information contained in this and other Sigma-Aldrich publications. Purchaser must determine the suitability of the product(s) for their particular use. Additional terms and conditions may apply. Please see reverse side of the invoice or packing slip.

Product Information

Monoclonal ANTI-FLAG® M2, Clone M2

Produced in Mouse, Purified Immunoglobulin

F3165

Product Description

Monoclonal ANTI-FLAG® M2 is a purified immunoglobulin, IgG1, monoclonal antibody, isolated from murine ascites fluid, that binds to FLAG® fusion proteins.[1] Unlike ANTI-FLAG® M1 antibody, the M2 antibody will recognize the FLAG® sequence at the N-terminus, Met-N-terminus, C-terminus, or at an internal site of FLAG® fusion proteins. Monoclonal ANTI-FLAG® M2 is useful for identification and capture of FLAG® fusion proteins by common immunological procedures such as Western blots and immuno-precipitation. It is also useful for affinity purification of FLAG® fusion proteins when bound to a solid support.

Monoclonal ANTI-FLAG® M2 binding is not dependent on calcium.

Reagent

This product is supplied in 10 mM sodium phosphate, 150 mM NaCl, pH 7.4, containing 0.02% sodium azide.

Precautions and Disclaimer

For R&D use only. Not for drug, household, or other uses. Please consult the Safety Data Sheet for information regarding hazards and safe handling practices.

Preparation Instructions

Dilute the antibody solution from 0.5–10 μg/mL in Tris Buffered Saline, pH 8.0, with 3% nonfat milk (Cat. No. T8793). Adjust the antibody concentration to maximize detection sensitivity and to minimize background.

Storage/Stability

Store the undiluted antibody at −20 °C in working aliquots. Repeated freezing and thawing is not recommended.

Note: Over time, small amounts of purified antibodies can precipitate from solution due to intermolecular hydrophobic interactions. If a precipitate is observed in this product, briefly centrifuge the vial to pellet the precipitate. Withdraw the desired volume of antibody solution from the clear supernatant for use.
This should not alter the performance of the purified antibody in Western blot or immunoprecipitation applications.

Procedure

Improved Western Blot Method for Detecting FLAG® Fusion Proteins using Monoclonal ANTI-FLAG® M2.

1. Separate FLAG® fusion proteins from sample lysates using a standard sodium dodecyl sulfate polyacrylamide gel electrophoresis (SDS-PAGE) protocol. Load 2.5–10 μg of total lysate protein per lane.
2. Transfer proteins from the gel to an Immobilon®-P or other polyvinylidene difluoride (PVDF) membrane. Nitrocellulose membranes can be used, but typically result in less sensitivity.
3. Wash the blot in at least 0.5 mL/cm^2 of Milli-Q® water for 2–3 minutes with mild agitation.
4. Block the blot with at least 0.5 mL/cm^2 of Tris Buffered Saline, pH 8.0, with 3% nonfat milk, or 50 mM Tris, 0.138 M NaCl, 2.7 mM KCl, pH 8.0, containing 30 mg/mL nonfat dry milk, for 30 minutes at room temperature with agitation (about 50–60 rpm).
5. Remove the blocking agent and wash once with 0.5 mL/cm^2 of TBS (Cat. No. T6664).
6. Add Monoclonal ANTI-FLAG® M2 to a final concentration of 10 μg/mL to the blot in at least 0.5 mL/cm^2 of TBS with 3% nonfat dry milk and incubate at room temperature for 30 minutes.

   Note: Using less Monoclonal ANTI-FLAG® M2 antibody may help to reduce background and cross-reactivity. See the Troubleshooting Guide.

7. Remove the Monoclonal ANTI-FLAG® M2 solution, and wash once with at least 0.5 mL/cm^2 of TBS.
8. Add Anti-Mouse IgG–Peroxidase (Cat. No. A9044) or equivalent, to at least 0.5 mL/cm^2 of TBS with 3% nonfat dry milk. Use the concentrations listed in Table 1. These concentrations are recommended starting concentrations for the antibodies used in Western blotting. Incubate the blots with shaking at room temperature for 30 minutes.

Table 1.

Antibody Concentrations

ANTI-FLAG® M2 primary antibody	Substrate	Secondary antibody concentration
0.5-10 µg/mL	ECL+™	1:80,000
0.5-10 µg/mL	ECL™	1:10,000

9. Wash the blot eight times for a total of 20 minutes in Tris Buffered Saline with TWEEN® 20, pH 8.0 (50 mM Tris, 0.138 M NaCl, 2.7 mM KCl, pH 8.0, plus 0.05% TWEEN® 20, Cat. No. T9039).
10. Develop the blots with the appropriate substrate for 5 minutes.
11. Expose BioMax™ light film to the blot. Exposure times range from 30 seconds to 10 minutes. It is best to do a quick exposure of 10–30 seconds to determine what exposure time is needed. If the signal is too intense even at the short exposure times, let the signal decay from 1–8 hours or longer if necessary, and then re-expose the film.

Immunofluorescence

Monoclonal ANTI-FLAG® M2 may be used in immunofluorescent procedures. A typical concentration for use is 20 µg/mL.[2]

Product Profile

Protein concentration (E_{280}): 2.0 – 5.0 mg/mL

Antigenic binding site:

N-Asp-Tyr-Lys-Asp-Asp-Asp-Asp-Lys-C

Specificity: Monoclonal ANTI-FLAG® M2 detects a single band of protein on a Western blot from an *E. coli* crude cell lysate.

Sensitivity: Monoclonal ANTI-FLAG® M2 detects 2 ng of FLAG-BAP™ fusion protein on a dot blot using chemiluminescent detection.

Note: In order to obtain best results, it is recommended that each individual user determine working dilution by titration assay.

References

1. Brizzard, B.L. *et al.*, *BioTechniques*, **16(4)**: 730-735 (1994).
2. Ciaccia, A.V., and Price, E.M., *IBI FLAG Epitope*, **1**: 4-5 (1992).
3. Bjerrum, O.J., and Heegaard. N.H.H., CRC Handbook of Immunoblotting of Proteins, Volume I, Technical Descriptions, CRC Press, pp. 229-236 (1988).
4. Dunbar, B.S. (ed.), *Protein Blotting: A Practical Approach*. IRL Press at Oxford University Press (Oxford, UK / New York, NY), pp. 67-70 (1994).
5. Fortin, A. *et al*., *Biochem. Cell Biol*., **72(5-6)**: 239-243 (1994).
6. Harlow, E., and Lane, D., *Antibodies: A Laboratory Manual*, pp. 726 (1989).
7. Cold Spring Harbor Laboratory Press (Cold Spring Harbor, NY) (1988).
8. Pampori, N.A. *et al*., *BioTechniques*, **18(4)**: 589-590 (1995).

Problem	Possible Cause	Solution
Fusion protein not detected	Protein not expressed	Verify nucleic acid sequence of FLAG® in vector construct. If sequence is present, attempt to optimize expression.
	Target protein poorly represented in sample	Positive controls should always be included. If the positive control works, the sample may not contain the FLAG® fusion protein of interest or it may be present at concentrations too low to detect. Immunoprecipitation with Monoclonal ANTI-FLAG® M2 Affinity Gel (Cat. No. A2220) may be required for low FLAG® fusion protein concentrations.<br>Positive controls available from Sigma:<br>• Amino-terminal FLAG-BAP™ Fusion Protein (Cat. No. P7582)<br>• Carboxy-terminal FLAG-BAP™ Fusion Protein (Cat. No. P7457)<br>• Amino-terminal Met-FLAG-BAP™ Fusion Protein (Cat. No. P5975)
	Defective detection reagents	Run appropriate controls to ensure performance. Use 10 ng/lane of a control FLAG-BAP™-fusion protein as a positive control. If no signal is obtained with the control, repeat the procedure using a newer lot of antibody-HRP conjugate and freshly prepared reagents.
	Inadequate exposure time using chemiluminescent system	If no signal is seen, expose for longer times. We recommends trying 30-second to 10-minute exposure times.
	Inappropriate film	Switch to film designated for chemiluminescent detection such as Kodak BioMax™ Light.
	No target protein present on membrane	Verify transfer by visualizing proteins on the membrane using a Ponceau S solution (Cat. No. P7170). If possible, a positive control should always be run to ensure that the components are functioning. Prestained protein markers (such as Cat. No. C1992 and C4861) may also be used to verify complete transfer.
	Antigen is covered by blocking reagent due to overblocking	Masking of a signal can occur if the blocking reagent (such as casein or gelatin blocking buffers, Cat. No. C7594 or G7663, respectively) is used at too high a concentration. A dilution of 1:1 to 1:3 may be done to decrease the concentration. If the problem persists, use TBS with 3% non-fat dry milk (Cat. No. T8793).
	Antibody concentration not optimal	Determine optimal working dilution for ANTI-FLAG® antibody by titration. Consider using more antibody if no signal or weak signal is detected. Also, antibody used at too high a concentration can also cause inhibition of signal especially in chemiluminescent detection systems.

Problem	Possible Cause	Solution
Cross-reactivity	Cellular extract concentration is too high	2.5 to 10 µg per lane of total lysate protein is usually enough to obtain a good signal. Load less cellular extract or serially dilute the cell extract to obtain the optimal signal to noise ratio.
	ANTI-FLAG® M2 antibody concentration is too high	Dilute ANTI-FLAG® M2 antibody from 0.1 to 0.5 µg/mL. Use TBS with 3% non-fat dry milk as diluent.
	Secondary antibody concentration is too high	We recommend initial dilutions of 1:10,000 for ECL™ and 1:80,000 for ECL+™. Further dilutions may be necessary
	ANTI-FLAG® M2 antibody cross-reacts with naturally occurring FLAG-like epitopes	Increasing the temperature to 37 °C during the blocking, binding and wash steps may reduce cross-reactivity. Lysates from mock-transfected controls (transfected with plasmid without insert DNA) will help distinguish the FLAG-fusion proteins from other cross-reacting proteins.

Notice

We provide information and advice to our customers on application technologies and regulatory matters to the best of our knowledge and ability, but without obligation or liability. Existing laws and regulations are to be observed in all cases by our customers. This also applies in respect to any rights of third parties. Our information and advice do not relieve our customers of their own responsibility for checking the suitability of our products for the envisaged purpose.

The information in this document is subject to change without notice and should not be construed as a commitment by the manufacturing or selling entity, or an affiliate. We assume no responsibility for any errors that may appear in this document.

Technical Assistance

Visit the tech service page at SigmaAldrich.com/techservice.

Standard Warranty

The applicable warranty for the products listed in this publication may be found at SigmaAldrich.com/terms.

Contact Information

For the location of the office nearest you, go to SigmaAldrich.com/offices.

The life science business of Merck operates
as MilliporeSigma in the U.S. and Canada.

Merck, FLAG, ANTI-FLAG, and Sigma-Aldrich are trademarks of Merck KGaA, Darmstadt, Germany or its affiliates. All other trademarks are the property of their respective owners. Detailed information on trademarks is available via publicly accessible resources.

© 2021 Merck KGaA, Darmstadt, Germany and/or its affiliates. All Rights Reserved.

F3165dat Rev 07/21

3050 Spruce Street, St. Louis, MO 63103 USA
Tel: (800) 521-8956 (314) 771-5765 Fax: (800) 325-5052 (314) 771-5757
email: techservice@sial.com sigma-aldrich.com

Product Information

Monoclonal Anti-HA antibody produced in mouse
clone HA-7, purified from hybridoma cell culture

Catalog Number **H3663**

Product Description

Monoclonal Anti-HA, Clone HA-7 (mouse IgG1 isotype) is derived from the HA-7 hybridoma produced by the fusion of mouse myeloma cells and splenocytes from a BALB/c mouse immunized with a synthetic peptide corresponding to amino acid residues YPYDVPDYA (98-106) of the human influenza virus hemagglutinin (HA), conjugated to KLH. The isotype is determined by a double diffusion immunoassay using Mouse Monoclonal Antibody Isotyping Reagents, Catalog No. ISO2.

The antibody recognizes an epitope located within the sequence YPYDVPDYA (residues 98-106) of the human influenza virus hemagglutinin (HA), known as the HA tag. The product is reactive with HA-tagged fusion proteins expressed at either the amino or the carboxy terminus of the fusion protein. Applications include ELISA, immunoblotting, immunocytochemistry, and immunoprecipitation.

Recombinant DNA technology enables the attachment of specific sequences to genes of interest to provide “affinity handles” (tags) designed to enable the selective identification and purification of the protein of interest.[1-6] The addition of a tag to a given gene creates a stable fusion product that may not interfere with the bioactivity of the protein, or with the biodistribution of the tagged product.

HA peptide is a nonapeptide derived from the hemagglutinin protein of the human influenza virus, which is a major spike membrane glycoprotein The nucleic acid sequence encoding this peptide (amino acids 98-106 of influenza HA) has been incorporated into various expression plasmids adjacent to the cloning site, thus enabling the cloning and expression of HA-tagged fusion proteins. Such fusion proteins may be expressed in cells of various organisms: bacteria, yeast, insects and mammals. In the fusion protein, the HA sequence may serve as a recognition target for specific antibodies. This enables detection, subcellular localization, characterization, quantification, functional analysis and affinity purification of the HA-tagged protein and associated bound proteins.[4] Insertion of the HA epitope in different regions of a cellular protein followed by examination of the immunoreactivity of the epitope in intact and permeabilized cells is useful for studying the cellular expression levels, topology and functional activity of the tagged protein.[7]

Monoclonal antibodies reacting specifically with HA may be useful in various immunotechniques, to identify the expression of an HA fusion protein *in situ* and by immunoblotting, in bacteria, bacterial lysates of cells and tissue transfected with HA fusion protein expressing vectors. It may also be used for the immunoprecipitation of HA fusion protein.

Reagent

The product is provided as purified IgG in 0.01 M phosphate buffered saline, pH 7.4, containing 1% BSA and 15 mM sodium azide as a preservative.

Antibody concentration: ~1 mg/ml.

Precautions and Disclaimer

This product is for R&D use only, not for drug, household, or other uses. Please consult the Material Safety Data Sheet for information regarding hazards and safe handling practices.

Storage/Stability

For continuous use, store at 2-8 °C for up to one month. For extended storage freeze in working aliquots. Repeated freezing and thawing, or storage in "frost-free" freezers, is not recommended. If slight turbidity occurs upon prolonged storage, clarify the solution by centrifugation before use. Working dilution samples should be discarded if not used within 12 hours.

Product Profile
Immunoblotting: a working concentration of 0.25-1 μg/ml is determined using cell extracts expressing N-terminal HA fusion proteins.

Immunoprecipitation: 0.5-2.0 μg of the antibody can immunoprecipitate a C-terminal HA fusion protein from transfected mammalian cell lysates.

Indirect immunofluorescence: a working concentration of 1.0-2.0 μg/ml is determined using 293-T cells transfected with N-terminal HA-fusion proteins, fixed with methanol/acetone.

Note: In order to obtain best results in different techniques and preparations we recommend determining optimal working dilution by titration test.

Procedures

Immunoblotting
All incubation steps should be performed at room temperature

1. Separate HA tagged proteins from sample lysates using a standard SDS-PAGE protocol. Load 2.5-20 μg total lysate protein per lane.
   Note: The amount of lysate to be loaded depends on the level of protein expression and may vary between experiments.
2. Transfer proteins from the gel to a nitrocellulose membrane.
3. Block the membrane using a solution of PBS containing 5% non-fat dry milk (PBS, Catalog No. D8537; non-fat dry milk, Catalog No. M7409) for at least 60 min.
4. Wash the membrane three times for 10 minutes each in PBS containing 0.05% TWEEN® 20, Catalog No. P3563.
   Note: blocking with PBS containing 1% BSA for 10 minutes at room temperature followed by draining prior to step 5 may minimize non-specific adsorption of the antibody.
5. Incubate the membrane with anti-HA antibody as the primary antibody in PBS containing 0.05% TWEEN 20, with agitation for 120 minutes.
6. Wash the membrane three times for 5 minutes each in PBS containing 0.05% TWEEN 20.
7. Incubate the membrane with Anti-Mouse IgG, peroxidase conjugate (Catalog Nos. A9917, A3682 or A2304) or with Anti-Mouse IgG, alkaline phosphatase conjugate (e.g. Catalog Nos. A1293, A2179 or A1682) as the secondary antibody, at the recommended concentration in PBS containing 0.05% TWEEN 20. Incubate for 60 minutes. Adjust the antibody concentration to maximize detection sensitivity and to minimize background.
8. Wash the membrane three times for 10 minutes each in PBS containing 0.05% TWEEN 20.
9. Treat the membrane with a peroxidase substrate.

Indirect Immunofluorescent staining of cultured cells
All incubation steps should be performed at room temperature (except step 3).

1. Grow transfected cultured cells expressing HA-tagged protein of choice on sterile coverslips at 37 °C.
2. Wash the cells briefly in PBS (Catalog No.D8537).
3. Fix the cells with –20 °C methanol (10 minutes) and then with –20 °C acetone (1 minute), **or** fix with 3% or 4% paraformaldehyde (10 minutes), rinse briefly with PBS and permeabilize with 0.5% Triton™ X-100 (2 minutes).
4. Wash coverslips twice in PBS (5 minutes each wash).
5. Incubate coverslips cell-side-up with Anti-HA in PBS containing 1% BSA, Catalog No. A9647, for 60 minutes.
6. Wash three times in PBS (5 minutes each wash).
7. Incubate coverslips cell-side-up with Anti-Mouse IgG, FITC conjugate (e.g. Catalog No. F4018 or F8771) as the secondary antibody, at the recommended dilution, in PBS containing 1% BSA, for 30 minutes.
8. Wash three times in PBS (5 minutes each wash).
9. Add one drop of aqueous mounting medium on the coverslip and invert carefully on a glass slide. Avoid air bubbles.
10. Examine using a fluorescence microscope with appropriate filters.

Immunoprecipitation

1. Centrifuge 20 μl of a 1:1 suspension of protein G-agarose beads, Catalog No.P3296, for 1 minute at 2000 x g, and then wash twice with 1 ml RIPA buffer (50 mM Tris Base, 0.25% w/v deoxycholate, 1% NP40, 150 mM NaCl, 1 mM EDTA, pH 7.4) at 4 °C.
2. Add anti-HA antibody diluted in PBS, and incubate by swinging head-over-tail for 1 hour at room temperature.
3. Centrifuge for 1 minute at 12,000 x g, and wash twice with 1 ml RIPA buffer at 4 °C by spinning.
4. Add 0.1-1.0 ml of cell extract containing HA tagged protein to the antibody-coupled beads (see Note), and incubate from 2 hours to overnight at 4 °C, while swinging head-over tail.

Note: The amount of cell extract depends on the level of expression of the tagged protein and the specific application.

5. Spin down beads; remove supernatant.
6. Wash beads four times with 1ml RIPA buffer and once with PBS by vortex and short spin.
7. Resuspend the pellet in 25 μl of 2X SDS-PAGE sample buffer. Boil sample for 5 minutes and spin down. The sample is ready to be loaded on an SDS-PAGE gel.

References

1. Narayanan, S.R., *J. Chromatogr.*, **658**, 237-258 (1994).
2. Olins, P.O., and Lee, S.C., *Curr. Opin. Biotechnol.*, **4**, 520-525 (1993).
3. Uhlen, M., and Moks, T., *Methods Enzymol.*, **185**, 129-143 (1990).
4. Kolodziej, P.A., and Young, R.A., *Methods Enzymol.*, **194**, 508-519 (1991).
5. Pines, J., and Hunter, T., *J. Cell Biol.*, **115**, 1-17 (1991).
6. Antebi, A., and Fink, G.R.L, *Mol. Biol. Cell*, **3**, 633-654 (1992).
7. Canfield, V.A., and Levenson, R., *Biochemistry*, **32**, 13782-13786 (1993).

Triton is a trademark of The Dow Chemical Company or an affiliated company of Dow

TWEEN is a registered trademark of Croda International PLC

DS,PHC 03/15-1

©2015 Sigma-Aldrich Co. LLC. All rights reserved. SIGMA-ALDRICH is a trademark of Sigma-Aldrich Co. LLC, registered in the US and other countries. Sigma brand products are sold through Sigma-Aldrich, Inc. Purchaser must determine the suitability of the product(s) for their particular use. Additional terms and conditions may apply. Please see product information on the Sigma-Aldrich website at www.sigmaaldrich.com and/or on the reverse side of the invoice or packing slip.

abcam

Product datasheet

Recombinant Human Androgen Receptor protein ab157902

1 Image

Description

**Product name**	Recombinant Human Androgen Receptor protein
**Expression system**	Wheat germ
**Protein length**	Protein fragment
**Animal free**	No
**Nature**	Recombinant
**Species**	Human
**Sequence**	SKDNYLGGTSTISDNAKELCKAVSVSMGLGVEALEHLSP<br>GEQLRGDCMYA<br>PLLGVPPAVRPTPCAPLAECKGSLLDDSAGKSTEDTAEY<br>SPFKGGYTKGL
**Amino acids**	221 to 320
**Tags**	GST tag N-Terminus

Specifications

Our Abpromise guarantee covers the use of **ab157902** in the following tested applications.

The application notes include recommended starting dilutions; optimal dilutions/concentrations should be determined by the end user.

**Applications**	ELISA<br>Western blot
**Form**	Liquid
**Additional notes**	Protein concentration is above or equal to 0.05 mg/ml.

Preparation and Storage

**Stability and Storage**	Shipped on dry ice. Upon delivery aliquot and store at -80ºC. Avoid freeze / thaw cycles.<br>pH: 8.00<br>Constituents: 0.31% Glutathione, 0.79% Tris HCl

[illegible]

General Info

**Function**	Steroid hormone receptors are ligand-activated transcription factors that regulate eukaryotic gene expression and affect cellular proliferation and differentiation in target tissues. Transcription factor activity is modulated by bound coactivator and corepressor proteins. Transcription activation is down-regulated by NR0B2. Activated, but not phosphorylated, by HIPK3 and ZIPK/DAPK3. Isoform 3 and isoform 4 lack the C-terminal ligand-binding domain and may therefore constitutively activate the transcription of a specific set of genes independently of steroid hormones.
**Tissue specificity**	Isoform 2 is mainly expressed in heart and skeletal muscle (PubMed:15634333). Isoform 3 is expressed by basal and stromal cells of prostate (at protein level) (PubMed:19244107).
**Involvement in disease**	Androgen insensitivity syndrome<br>Spinal and bulbar muscular atrophy X-linked 1<br>Defects in AR may play a role in metastatic prostate cancer. The mutated receptor stimulates prostate growth and metastases development despite of androgen ablation. This treatment can reduce primary and metastatic lesions probably by inducing apoptosis of tumor cells when they express the wild-type receptor.<br>Androgen insensitivity, partial
**Sequence similarities**	Belongs to the nuclear hormone receptor family. NR3 subfamily.<br>Contains 1 nuclear receptor DNA-binding domain.
**Domain**	Composed of three domains: a modulating N-terminal domain, a DNA-binding domain and a C-terminal ligand-binding domain. In the presence of bound steroid the ligand-binding domain interacts with the N-terminal modulating domain, and thereby activates AR transcription factor activity. Agonist binding is required for dimerization and binding to target DNA. The transcription factor activity of the complex formed by ligand-activated AR and DNA is modulated by interactions with coactivator and corepressor proteins. Interaction with RANBP9 is mediated by both the N-terminal domain and the DNA-binding domain. Interaction with EFCAB6/DJBP is mediated by the DNA-binding domain.
**Post-translational modifications**	Sumoylated on Lys-388 (major) and Lys-521. Ubiquitinated. Deubiquitinated by USP26. 'Lys-6' and 'Lys-27'-linked polyubiquitination by RNF6 modulates AR transcriptional activity and specificity.<br>Phosphorylated in prostate cancer cells in response to several growth factors including EGF. Phosphorylation is induced by c-Src kinase (CSK). Tyr-535 is one of the major phosphorylation sites and an increase in phosphorylation and Src kinase activity is associated with prostate cancer progression. Phosphorylation by TNK2 enhances the DNA-binding and transcriptional activity and may be responsible for androgen-independent progression of prostate cancer. Phosphorylation at Ser-83 by CDK9 regulates AR promoter selectivity and cell growth. Phosphorylation by PAK6 leads to AR-mediated transcription inhibition.<br>Palmitoylated by ZDHHC7 and ZDHHC21. Palmitoylation is required for plasma membrane targeting and for rapid intracellular signaling via ERK and AKT kinases and cAMP generation.
**Cellular localization**	Nucleus. Cytoplasm. Predominantly cytoplasmic in unligated form but translocates to the nucleus upon ligand-binding. Can also translocate to the nucleus in unligated form in the presence of RACK1.

Images

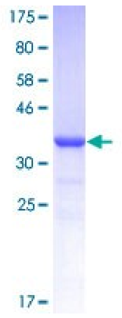

SDS-PAGE - Recombinant Human Androgen Receptor protein (ab157902)

ab157902 on a 12.5% SDS-PAGE stained with Coomassie Blue.

Please note: All products are "FOR RESEARCH USE ONLY. NOT FOR USE IN DIAGNOSTIC PROCEDURES"

Our Abpromise to you: Quality guaranteed and expert technical support

- Replacement or refund for products not performing as stated on the datasheet
- Valid for 12 months from date of delivery
- Response to your inquiry within 24 hours

- We provide support in Chinese, English, French, German, Japanese and Spanish
- Extensive multi-media technical resources to help you
- We investigate all quality concerns to ensure our products perform to the highest standards

If the product does not perform as described on this datasheet, we will offer a refund or replacement. For full details of the Abpromise, please visit https://www.abcam.com/abpromise or contact our technical team.

Terms and conditions

- Guarantee only valid for products bought direct from Abcam or one of our authorized distributors
